# Human embryonic stem cell-derived dopaminergic cells for Parkinson’s disease: a phase 1/2 open-label trial

**DOI:** 10.1038/s41591-026-04525-0

**Published:** 2026-07-09

**Authors:** G. Paul, H. Bjartmarz, A. Kirkeby, J. Nelander, R. Smith, S. Kayhanian, A. Evans, B. Harry, E. Cutting, S. Fazal, N. P. Lao-Kaim, T. van Vliet, S. Ullén, I. Grubor, O. Hansson, P. Piccini, O. Lindvall, A. Björklund, H. Widner, M. Parmar, R. A. Barker

**Affiliations:** 1 Department of Neurology, Skåne University Hospital, Lund, Sweden; 2 Department of Clinical Sciences and Wallenberg Center for Molecular Medicine, Lund University, Lund, Sweden; 3 Department of Neurosurgery, Skåne University Hospital, Lund, Sweden; 4 Novo Nordisk Foundation Center for Stem Cell Medicine (reNEW), Department of Biomedical Sciences, Faculty of Health and Medical Sciences, University of Copenhagen, Copenhagen, Denmark; 5 Department of Experimental Medical Science, Wallenberg Neuroscience Center and Lund Stem Cell Center, Lund University, Lund, Sweden; 6 Clinical Memory Research Unit, Department of Clinical Sciences in Malmö, Lund University, Lund, Sweden; 7 Department of Clinical Neurosciences, University of Cambridge, Cambridge, UK; 8 Department of Brain Sciences, Imperial College London, London, UK; 9 Clinical Studies Sweden - Forum South, Skåne University Hospital, Lund, Sweden; 10 Lund Stem Cell Center and Division of Neurology, Department of Clinical Sciences Lund, Lund University, Lund, Sweden; 11 Cambridge Stem Cell Institute, Jeffrey Cheah Biomedical Centre, Cambridge Biomedical Campus, University of Cambridge, Cambridge, UK

**Keywords:** Stem-cell research, Parkinson's disease

## Abstract

Parkinson’s disease (PD) is characterized by progressive loss of nigral dopaminergic neurons, resulting in disabling motor symptoms. Intracerebral transplantation of stem cell-derived dopaminergic progenitors to replace lost endogenous dopaminergic neurons offers a new potentially restorative therapeutic approach for PD. Here we report the 12-month primary safety end point and interim efficacy outcomes from a phase 1/2, open-label, multicenter trial evaluating STEM-PD, a cryopreserved, off-the-shelf dopaminergic progenitor product derived from human pluripotent stem cells. Eight individuals with moderate PD underwent bilateral intraputaminal transplantation at two escalating doses (*n* = 4 per cohort), followed by 12 months of immunosuppression. Seven participants completed 12-month follow-up; one participant died from a pulmonary infection. No serious adverse events were attributed to the cell product, no graft-induced dyskinesias were observed and serial magnetic resonance imaging showed no evidence of tumor formation. These findings support the feasibility and favorable safety profile of human pluripotent stem cell-derived dopaminergic progenitor transplantation in this early-phase study, with risks primarily associated with the immunosuppression regimen. Ongoing follow-up to 36 months will further evaluate durability, clinical outcomes and graft function. ClinicalTrials.gov identifier: NCT05635409.

## Abstract (web summary)

A phase 1/2 trial of an off-the-shelf stem cell-derived dopaminergic progenitor therapy demonstrated a favorable 12-month safety profile, with no graft-related adverse events, dyskinesias or tumor formation, although risks were associated with the immunosuppression regimen.

## Main

Parkinson’s disease (PD) is the second most common neurodegenerative disorder after Alzheimer’s disease. Neurological conditions are currently the leading global cause of disability, with PD being the fastest growing among them in terms of prevalence, disability-adjusted life years and mortality rates^1^. As of 2019, more than 8.5 million individuals worldwide were estimated to be living with PD, a number projected to exceed 12 million by 2040^2^.

The hallmark pathology of PD involves progressive degeneration of dopaminergic neurons in the substantia nigra pars compacta (SNpc) and the subsequent loss of their projections to the striatum. This results in striatal dopamine deficiency, which underlies many of the cardinal motor features of the disease: rigidity, bradykinesia and in some cases tremor^3,4^. At the time of clinical diagnosis, more than 50% of dopaminergic neurons in the SNpc and over 70% of striatal innervation have typically already been lost^5^.

Current therapies for PD are primarily symptomatic and do not halt disease progression. Pharmacological treatments aim to restore dopaminergic tone using levodopa (L-dopa), dopamine agonists and enzyme inhibitors or to modulate other neurotransmitter systems (for example, amantadine) to counteract some of the motor complications of long-term L-dopa treatment such as L-dopa-induced dyskinesias (LIDs). These treatments, although initially highly effective, become less reliable with disease progression, as patients often develop motor complications, including fluctuations and dyskinesias, as well as nonmotor side effects such as hallucinations^6^. Approximately 10% of PD cases present before the age of 50 years, with many of these individuals expected to live with the disease and the progressively worsening medication side effects for more than two decades.

Device-assisted therapies such as deep brain stimulation (DBS) and magnetic resonance imaging (MRI)-guided focused ultrasound are used to alleviate some of these complications, along with dopaminergic pump therapies^6,7^. However, none of these interventions restore the underlying neuronal loss or the dopaminergic transmission back to normal^8^.

Cell-based replacement strategies aim to address this unmet need by reintroducing functional dopaminergic neurons into the striatum. Early clinical trials using human fetal ventral mesencephalic (VM) tissue demonstrated proof of principle: transplanted dopaminergic neurons can survive long term in the host brain^9^ and, in some patients, provide durable symptomatic benefit over decades^10^. In these patients, the storage and release of dopamine had been restored to normal levels in grafted striatal areas. However, outcomes have been inconsistent across studies^11–13^ and the use of fetal tissue is limited by ethical, logistical and standardization challenges^14,15^.

These limitations have catalyzed efforts to develop standardized and scalable dopamine cell therapy products derived from pluripotent stem (PS) cells, including human embryonic stem (ES) cells and induced PS (iPS) cells. Several such products are now in clinical development (see refs. ^16,17^ for review), with early safety and feasibility data emerging^18–20^.

The STEM-PD trial was initiated to evaluate the safety, tolerability and feasibility of intraputaminal transplantation of STEM-PD, a cryopreserved dopaminergic progenitor cell product derived from the clinical-grade human ES cell line RC17^21^, in patients with moderately advanced PD. This 3-year phase 1/2, open-label, multicenter, single-arm, dose-escalation trial was conducted under an advanced therapy investigational medicinal product (ATIMP) framework.

The primary end point of this trial is defined as the number and nature of adverse events (AEs) and serious AEs (SAEs) occurring within the first 12 months following transplantation and the absence of space-occupying lesions on cranial MRI during the same period. Secondary and exploratory outcomes will evaluate the course and efficacy of clinical features, the survival of grafted dopaminergic cells at 36 months as well as additional safety signals occurring between 12 and 36 months and any dose–response effects.

Here, we report the 12-month primary end point safety data and interim efficacy data of the trial.

## Results

### Demographics

**Fig. 1:**
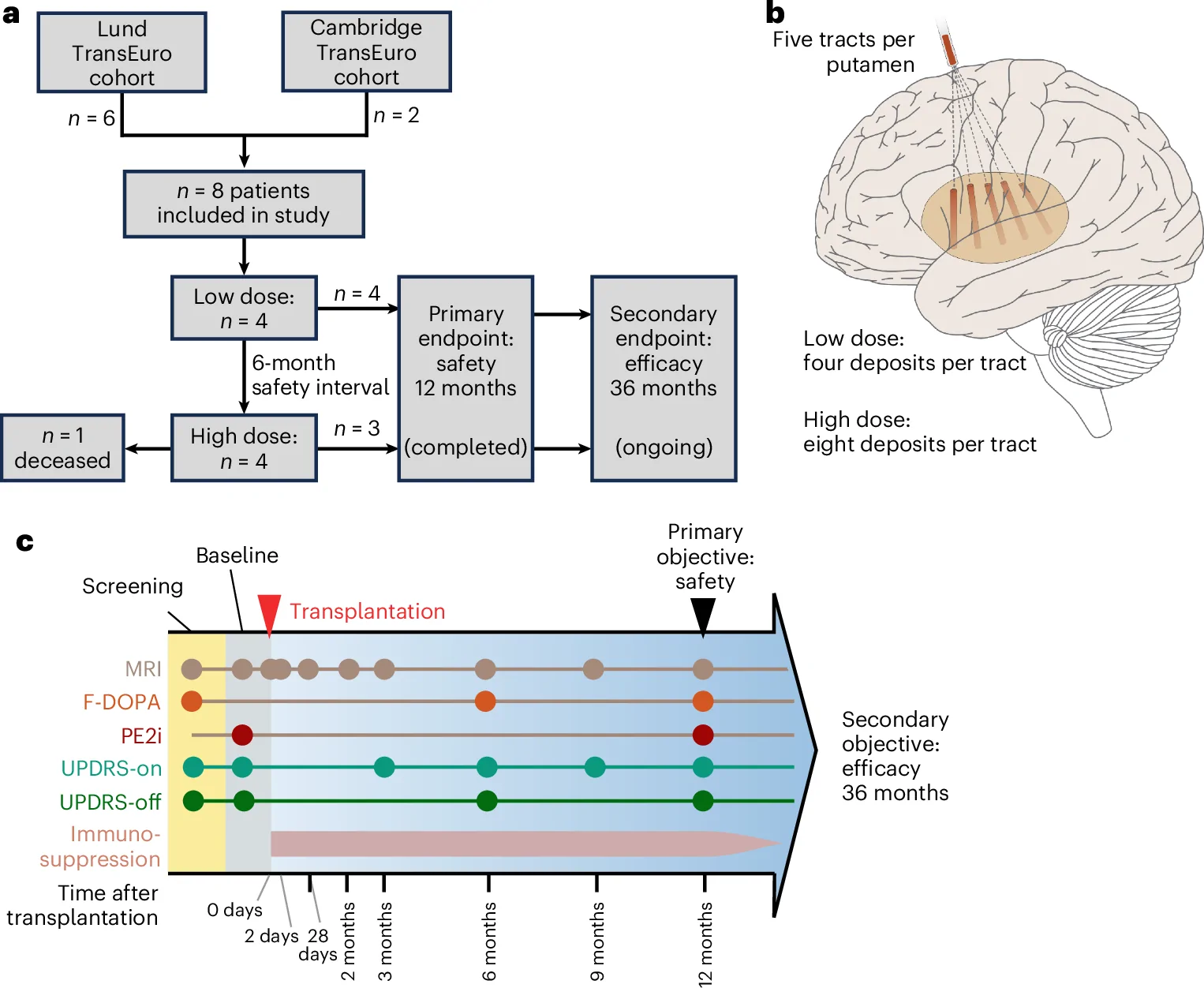
Study design, participant allocation and assessment schedule. **a**, Participants were enrolled at Skåne University Hospital (*n* = 6) and Addenbrooke’s Hospital (*n* = 2) between 7 December 2022 and 13 September 2024. The first four participants were allocated to the low dose and the subsequent four to the high dose. One participant in the high-dose group died 10 weeks after transplantation; safety data are reported up to the time of death. Follow-up of the remaining seven participants is ongoing. Transplantations were conducted in a staggered manner with predefined safety intervals. Safety data were reviewed by the Trial Management Group (TMG) and the Data Safety Monitoring Board (DSMB) before each subsequent transplantation and before dose escalation, following review of safety data up to 6 months after transplantation of participant 4. **b**, Cells were administered along five tracts per hemisphere into the putamen. **c**, Schedule of safety MRI, [^18^F]F-DOPA PET and [^18^F]FE-PE2i PET imaging, immunosuppression and clinical assessments up to the 12-month primary end point.

A total of eight participants were screened (all recruited from the TransEuro observational cohort^15^) and sequentially enrolled into either the low-dose (*n* = 4) or high-dose (*n* = 4) cohort. The first participant was enrolled on 3 January 2023 and the last on 13 September 2024. One participant in the high-dose cohort died 10 weeks after transplantation from a fungal pulmonary infection. Safety data from this individual are included through to the time of death. All remaining participants completed the 12-month follow-up and continue to be assessed as part of the trial (Fig. 1a). Across both dose groups, the median age at baseline was 63 years (range 58–70 years), the median disease duration 14.5 years (range 12–18 years) and six participants (75%) were male (Table 1). All patients were graded Hoehn–Yahr stage 2 in the OFF state and none of the participants had LIDs exceeding a score of 2 points in any body part using the Abnormal Involuntary Movement Scale (AIMS) for dyskinesia assessment. The adjusted OFF time was 2 h (range 1–12 h) in the low-dose group and 3 h (range 0–4 h) in the high-dose group (Table 1).

**Table 1:** Demographics and baseline characteristics

Characteristic	Low dose (*n* = 4)	High dose (*n* = 4)
Age^a^ (years)	63 (59, 65)	62 (58, 70)
Male gender, *n* (%)	4 (100%)	2 (50%)
Disease duration^a^ (years)	13 (12, 17)	15 (14, 18)
Hoehn–Yahr stage 2 in OFF	4 (100%)	4 (100%)
MDS-UPDRS-III (motor) score OFF^a^	27 (17, 33)	27 (16, 39)
MDS-UPDRS-III (motor) score ON^a^	12 (4, 24)	10 (4, 33)
AIMS score in ON >2 points per body part	0 (100%)	0 (100%)
LEDD^a^	1,071 (674, 1,539)	1,318 (750, 1,811)
Adjusted ON time without dyskinesia	14 (4, 15)	13 (13, 16)
Adjusted OFF time	2 (1, 12)	3 (0, 4)
MoCA^a^	27 (26, 28)	28 (25, 30)
MADRS^a^	4 (3, 4)	2 (0, 7)

^a^Values are median ± minimum/maximum.

^b^Hoehn–Yahr stages range from 1 to 5.

AIMS, Abnormal Involuntary Movement Scale; LEDD, levodopa equivalent daily dose; MADRS, Montgomery–Åsberg Depression Rating Scale; MDS-UPDRS, Movement Disorder Society-Unified Parkinson’s Disease Rating Scale; MoCA, Montreal Cognitive Assessment.

### Primary outcome

Three SAE’s occurred in three participants during the 12-month follow-up period and are presented in Table 2. One patient (low dose) required prolonged hospitalization owing to transient worsening of parkinsonian symptoms and anxiety after transplantation following temporary discontinuation of dopaminergic medication during surgery and in response to anesthesia medications. The symptoms resolved fully over time with reintroduction of the regular dopaminergic therapy. Another participant (high dose) experienced visual hallucinations 5 months after the transplant. This episode followed an adjustment of medications, specifically an increased dose of a dopamine agonist, and occurred in association with a urinary tract infection, necessitating brief hospitalization. The symptoms resolved with medical management and did not return. The third SAE occurred in the patient (high dose) who developed a disseminated pulmonary aspergillosis infection with subsequent central nervous system involvement while on active immunosuppression (azathioprine, tacrolimus and corticosteroids) and died 10 weeks after transplantation despite antifungal medication and withdrawal of immunosuppression. This patient was the second-to-last to undergo transplantation and, at that time, all participants had received a transplant. Only one patient remained on a high-dose steroid taper, and the tapering regimen for this patient was subsequently modified.

**Table 2:** Summary of SAEs

SAE no.	Status	AE term	Seriousness category	Severity^a^	Causality^b^				Expectedness
					Cell product	IS	Surgery	Device	
1	Resolved with no residual effects	Worsening of participant’s PD	Prolonged hospitalization	3	4	5	2	5	Expected reaction to surgical procedures
2	Resolved with no residual effects	Hallucinations, visual	Required hospitalization	3	5	3	5	5	Not an expected reaction
3	Death	Acute kidney injury, pneumonia fungal	Resulted in death	5	4	2	5	5	Expected reaction to tacrolimus, azathioprine and prednisolone

^a^Severity: 1 = mild, 2 = moderate, 3 = severe, 4 = life threatening, 5 = resulting in death.

^b^Causality grading: 1 = definitely related, 2 = probably related, 3 = possibly related, 4 = unlikely to be related, 5 = definitely not related, 6 = not applicable.

AE, adverse event; IS, immunosuppression; PD, Parkinson’s disease; SAE, serious adverse event.

None of the SAEs were attributed to the cell product itself or the surgical device. Importantly, no MRI evidence of tumor formation or abnormal tissue growth was observed in any patient during the 12-month follow-up period.

A total of 373 non-serious AEs were reported up to the 12-month end point (194 events in the low-dose and 179 events in the high-dose cohort; Table 3). The most frequently reported AEs were laboratory abnormalities (65%), defined in this trial as any deviation from the reference range, irrespective of clinical relevance (Extended Data Table 1). AEs occurring after study intervention were predominantly mild (45% low dose; 44% high dose) or moderate in severity and are presented according to their assessed possible relationship to immunosuppression, surgery or the cell product in Table 3. Importantly, no graft-induced dyskinesias (GIDs) were observed in the OFF state in any patient. One minor, asymptomatic hemorrhage occurred during surgery in one patient along one of the needle tracts. This resolved without sequelae.

**Table 3:** Summary of (non-serious) AEs

	All^a^ AEs		Immunosuppression		Surgery		Cell product	
	Low dose	High dose	Low dose	High dose	Low dose	High dose	Low dose	High dose
AE	194 (52%)	179 (48%)	64 (33%)	100 (56%)	29 (15%)	17 (9%)	2 (1%)	0 (0%)
Severity^b^								
Grade 1	169 (45%)	163 (44%)	55 (86%)	87 (87%)	27 (93%)	15 (88%)	2 (100%)	0 (0%)
Grade 2	25 (7%)	13 (3%)	9 (14%)	10 (10%)	2 (7%)	2 (12%)	0 (0%)	0 (0%)
Grade 3	0 (0%)	3 (1%)	0 (0%)	3 (3%)	0 (0%)	0 (0%)	0 (0%)	0 (0%)
Grade 4	0 (0%)	0 (0%)	0 (0%)	0 (0%)	0 (0%)	0 (0%)	0 (0%)	0 (0%)
Grade 5	0 (0%)	0 (0%)	0 (0%)	0 (0%)	0 (0%)	0 (0%)	0 (0%)	0 (0%)
Causality^c^								
Definitely related	NA	NA	10 (15%)	7 (7%)	2 (7%)	4 (24%)	0 (0%)	0 (0%)
Probably related	NA	NA	7 (11%)	7 (7%)	2 (7%)	4 (24%)	0 (0%)	0 (0%)
Possibly related	NA	NA	48 (74%)	86 (86%)	25 (86%)	9 (53%)	2 (100%)	0 (0%)
Unlikely to be related	NA	NA	0 (0%)	0 (0%)	0 (0%)	0 (0%)	0 (0%)	0 (NA%)
Definitely not related	NA	NA	0 (0%)	0 (0%)	0 (0%)	0 (0%)	0 (0%)	0 (NA%)

^a^Non-serious.

^b^Severity: 1 = mild, 2 = moderate, 3 = severe, 4 = life threatening, 5 = resulting in death.

^c^Causality grading: 1 = definitely related, 2 = probably related, 3 = possibly related, 4 = unlikely to be related, 5 = definitely not related, 6 = not applicable.

AE, adverse event; NA, not applicable.

Two AEs (both low dose) were deemed possibly related to the cell product. One AE involved the re-emergence of ON dyskinesias in one participant that were present from 5 to 10 months after transplantation, despite a reduction in dopaminergic medication, and causality to the ATIMP could not be ruled out. The participant has since stabilized, indicating that this AE was not related to the mature, functional graft. In another participant, a transient increase in parkinsonian symptoms was noted in the first 3 weeks after the operation. This patient did not respond well to medication during a period of approximately 1 week, and a possible contribution of surgery, concomitant medications and/or the ATIMP (graded ‘possibly related’) could not be ruled out.

A higher proportion of AEs was attributed to immunosuppression (33% in the low-dose group, 56% in the high-dose group; Table 3), including abnormal blood tests as expected. These events were manageable and consistent with the known profiles of immunosuppressive therapies. At the time of this interim analysis, all surviving participants have either completed or are tapering off their immunosuppressive therapy, as per the protocol.

### Assessment of grafting procedure

**Fig. 2:**
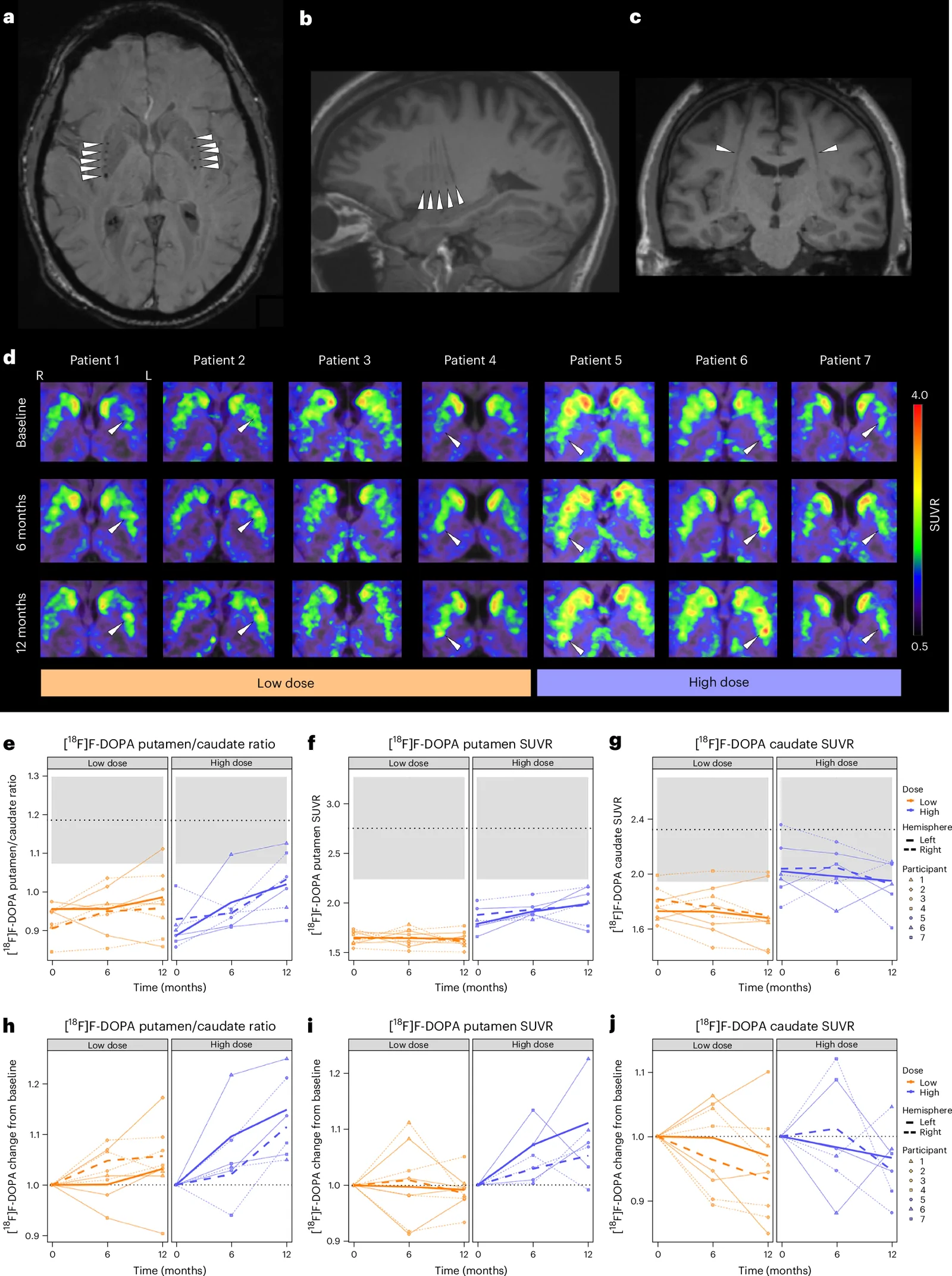
MRI and PET. **a**–**c**, Representative MRI image showing the placement of the five injection tracts in a representative participant in the transversal plane (**a**), sagittal plane (**b**) and coronal plane (**c**). Injection tracts are indicated with white arrowheads. **d**, [^18^F]F-DOPA PET transversal images of the basal ganglia in the seven participants. Images show corresponding slices from baseline (top), 6-month (middle) and 12-month (bottom) scans, overlaid on the baseline MRI. Images are shown as SUVR images with an occipital reference region. Participants are presented in random order within the dose groups (with numbers corresponding to lines in graphs in **e**–**j**). Areas with focal increase are indicated with white arrowheads. Images are shown in radiological orientation. **e**, [^18^F]F-DOPA data as putamen/caudate ratios. **f**, [^18^F]F-DOPA data as SUVR in the putamen. **g**, [^18^F]F-DOPA data as caudate SUVRs. The dotted black horizontal line in **e**–**g** corresponds to the mean in healthy controls and the gray shaded area to mean ± 2 s.d. in healthy controls. **h**, Putamen/caudate ratios normalized to baseline values. **i**, Putamen SUVR relative to baseline values. **j**, Caudate SUVRs relative to the baseline value. In **e**–**j**, the data are presented separately for the two dose groups. Thick orange/blue lines indicate the mean value for all participants (left hemisphere, solid line; right hemisphere, dashed line). The dotted black lines in **h**, **i** and **j** indicate the normalized baseline level. Source data

All five tracts per hemisphere were confirmed to be accurately placed within the putamen in each participant, as assessed by postoperative cranial MRI (Fig. 2a–c). Notably, two participants exhibited T2-weighted MRI hyperintensities outside the graft site at 1 and 6 months after transplantation, respectively, consistent with gliosis along the needle tract, similar to findings reported in a comparable trial^19^. One minor, asymptomatic hemorrhage occurred in a single trajectory (1 out of 80 trajectories), which resolved without sequelae.

### Secondary outcomes

#### Assessment of graft survival

[^18^F]F-DOPA imaging was performed at screening, 6 and 12 months to assess graft survival and dopaminergic function. Interim imaging analysis demonstrated focal increases in [^18^F]F-DOPA uptake in the grafted regions compared with baseline in several participants, interpreted as evidence of cell survival (Fig. 2d–j). In the low-dose group, the putamen/caudate [^18^F]F-DOPA uptake ratio at 12 months increased by 3.2% (mean ± s.d., 1.032 ± 0.11) on the left side and by 5.7% (1.057 ± 0.03) on the right side compared with baseline (Fig. 2e,h). In the high-dose group, the ratio increased by 14.9% (1.149 ± 0.095) on the left side and by 11.5% (1.115 ± 0.085) on the right. For example, participant 6 in the high-dose group showed a 22.5% increase in F-DOPA uptake in the left putamen, corresponding to an increase of 24.9% in the putamen/caudate ratio (Fig. 2d,f,i). As expected, the caudate uptake decreased by 3–7% in all participants over 12 months. Results for putamen and caudate standardized uptake value ratios (SUVRs) at 12 months relative to baseline are presented in Extended Data Table 2 and shown in Fig. 2f,g,i,j. Owing to the low number of participants, statistical analysis was only performed for the whole dataset (both doses combined). Using paired Wilcoxons signed-rank tests, we found a significant difference in the putamen/caudate ratio on the right side (*P* = 0.016) but no significant effect with respect to the left putamen/caudate ratio (*P* = 0.109). No other comparisons were significant at the 6- or 12-month time point of graft assessment.

**Extended Data Fig. 1:**
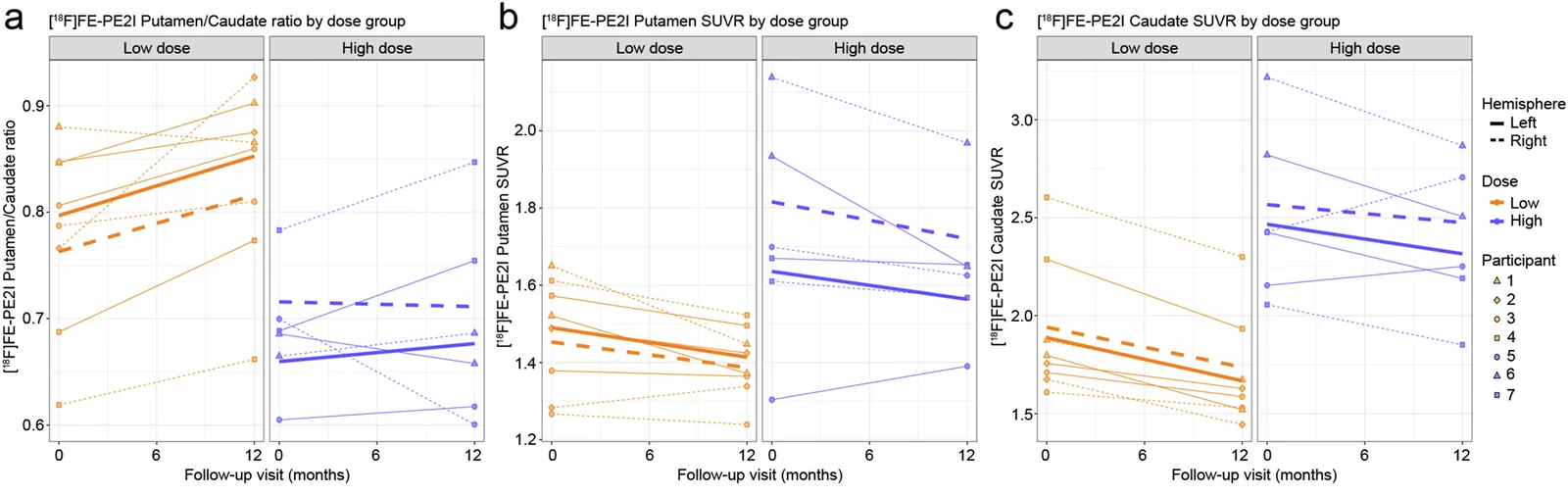
[^18^F]FE-PE2I. **a**, [^18^F]FE-PE2I data as putamen/caudate ratios. **b**, [^18^F]FE-PE2I data as standardized uptake value ratios (SUVR) in the putamen. **c**, [^18^F]FE-PE2I data as caudate SUVRs. Thick lines in a-c indicate the mean value for all participants (left - solid line, right -dashed line. The data is presented separately for the two dose groups, as indicated above. Source data

Dopamine transporter positron emission tomography (PET) imaging with [^18^F]FE-PE2i was performed at baseline and at 12 months, as per the study protocol. We did not observe significant differences at 12 months (Extended Data Fig. 1).

#### Development of clinical outcomes up to 12 months

**Fig. 3:**
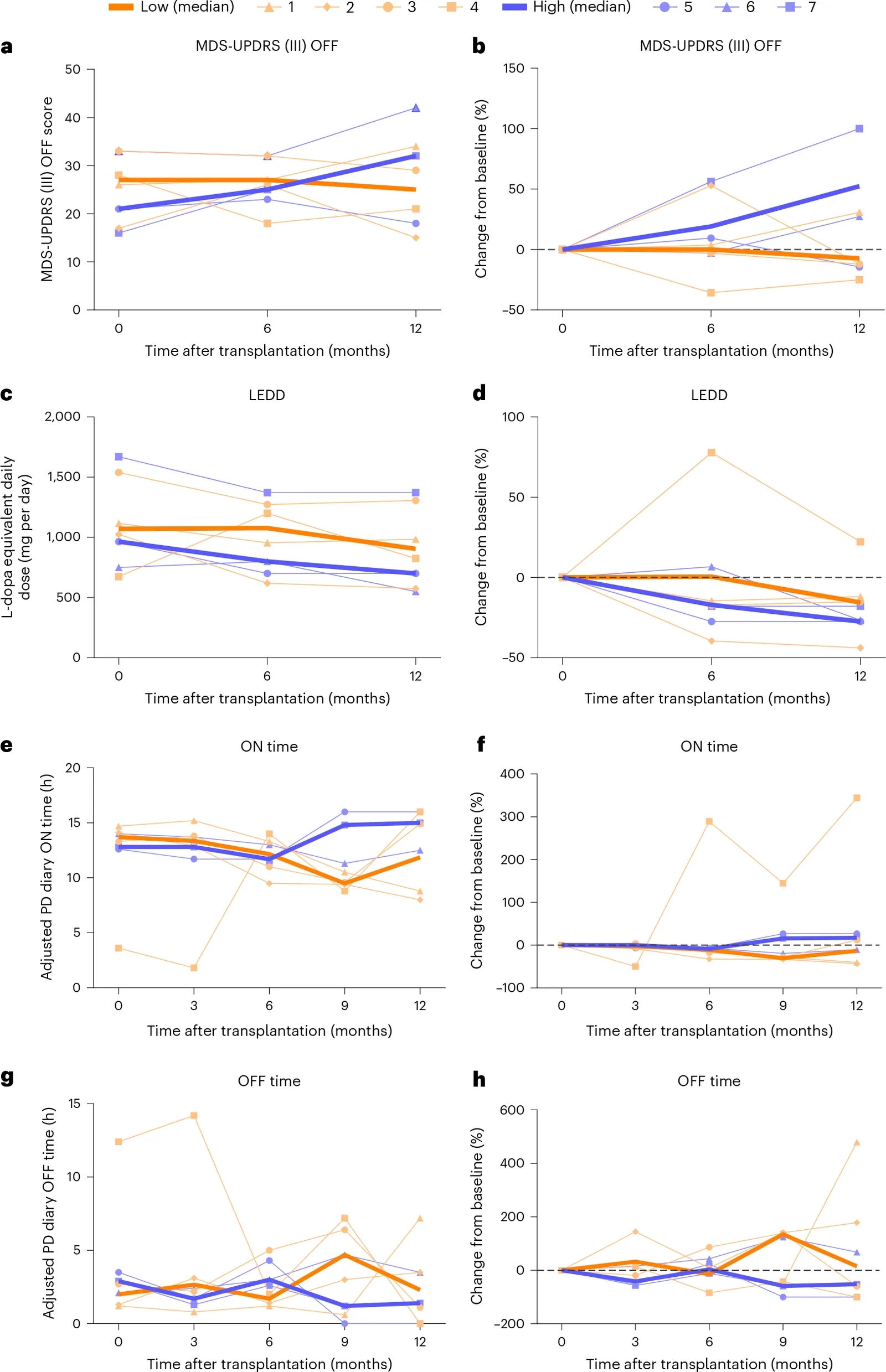
Preliminary efficacy outcomes. **a**, MDS-UPDRS part III OFF scores for all participants at baseline and at 6 and 12 months. **b**, Change in MDS-UPDRS part III OFF scores from baseline, expressed as percentage change. **c**, LEDD at baseline and at 6 and 12 months in both dose cohorts. **d**, Change in LEDD from baseline, expressed as percentage change. **e**, Adjusted patient-reported ON time without troublesome dyskinesias, assessed using the Hauser diary. **f**, Change in adjusted patient-reported ON time without troublesome dyskinesias from baseline, assessed using the Hauser diary. **g**, Patient-reported adjusted OFF time, assessed using the Hauser diary. **h**, Change in adjusted patient-reported OFF time from baseline, assessed using the Hauser diary. Participants are presented in random order within dose groups. Low dose (orange) and high dose (blue) are shown, and the median for each dose cohort is indicated by a thicker line. Source data

Motor function as measured by the Movement Disorder Society-Unified Parkinson’s Disease Rating Scale (MDS-UPDRS) part III in the OFF-medication state (OFF being defined as receiving no dopamine therapy for 12 hours or no long-acting dopamine agonists for 24 h before being tested) improved in three of four participants in the low-dose group and in one of three participants in the high-dose group between baseline and 12 months. In the low-dose group, MDS-UPDRS (III) OFF scores (median (range)) changed by −3.0 points (−7.0 to 8.0) at 12 months compared with baseline and in the high-dose group by +9.0 points (−3.0 to 16.0) (Fig. 3a,b).

Six out of seven patients substantially reduced their medication over this 12-month period. Overall, L-dopa equivalent daily dose (LEDD^22^) was reduced compared with baseline by 15.6% in the low-dose cohort and 27.5% in the high-dose group (Fig. 3c,d).

Adjusted patient-reported ON-time without troublesome dyskinesia, as captured by Hauser diary entries, changed from (median (range)) 13.7 (3.6 to 14.7) h per day at baseline to 11.8 (8 to 16) h per day at 12 months in the low-dose group and from 12.8 (12.6 to 14) h to 15 (12.5 to 16) h in the high-dose group (Fig. 3e,f).

Adjusted patient-reported OFF time was unchanged in the low-dose group consisting of (median (range)) 2 (1.2 to 12.4) h at baseline and 2.3 (0 to −7.2) h at 12 months, while it decreased in the high-dose group from 2.9 (2 to 2.9) h at baseline to 1.4 (1.4 to 3.5) h at 12 months (Fig. 3g,h).

**Extended Data Fig. 2:**
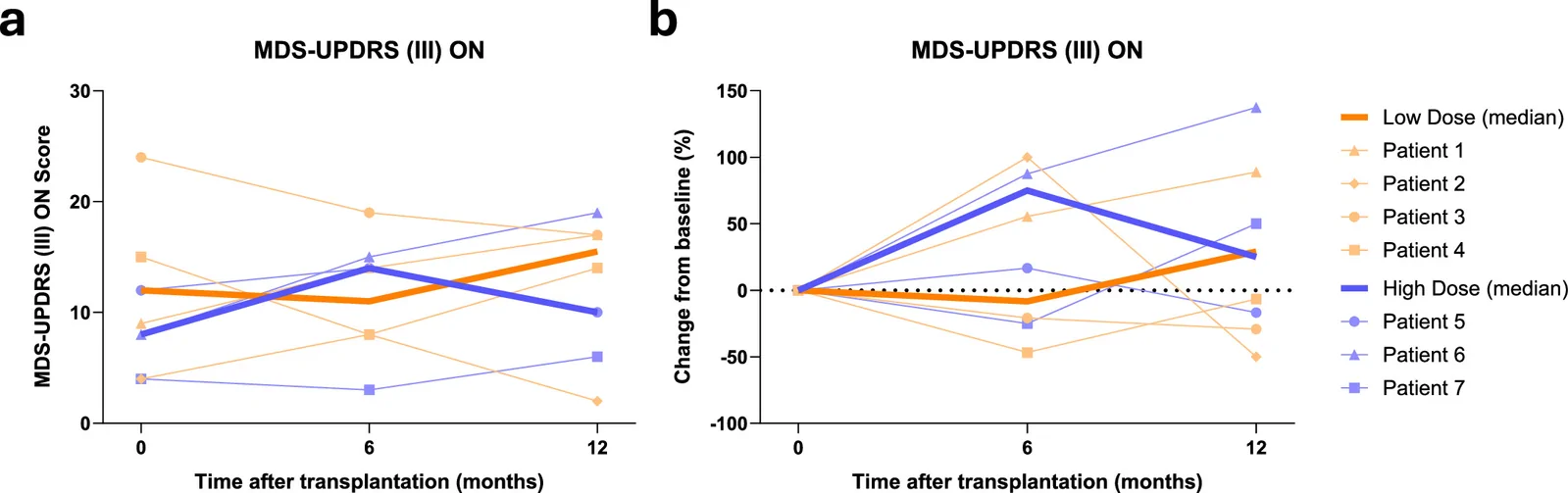
MDS-UPDRS Part III ON. **a**, MDS-UPDRS Part III ON scores at baseline and at 6 and 12 months for all participants in the low dose (orange lines) and the high dose (blue lines). The median for each dose group is shown as a darker line. **b**, Change in MDS-UPDRS Part III ON scores from baseline, expressed as percent change. Source data

MDS-UPDRS part III scores in the ON-medication state at baseline were fairly stable over the 12-month period in both dose groups (median (range)) (low dose, 12 (4 to 24) at baseline and 15.5 (2 to 17) at 12 months; high dose, 8 (4 to 12) at baseline and 10 (6 to 19) at 12 months) (Extended Data Fig. 2).

Nonmotor symptoms did not show major changes over the 12-month follow-up period in either dose group. MDS-UPDRS part I scores remained stable, with a median (median (range)) of 6 points at baseline (2 to 10) and at 12 months (2 to 13). MDS-UPDRS part II scores decreased from 10 points (5 to 12) at baseline to 8 points (5 to 11) at 12 months, whereas MDS-UPDRS part IV scores increased from 4 (3 to 7) at baseline to 5 (0 to 10) at 12 months. Cognitive performance, assessed using the Montreal Cognitive Assessment (MoCA), remained stable over follow-up, with median scores (median (range)) of 27 (25 to 30) at baseline and 28 (24 to 30) at 12 months. Depressive symptoms, assessed using the Montgomery–Åsberg Depression Rating Scale (MADRS), also remained stable, with median scores of 4 (1 to 7) at baseline and 5 (2 to 11) at 12 months. Health-related quality of life, measured using the EuroQol – 5 Dimensions – 5 Levels (EQ-5D-5L) total score, showed no relevant change, with median scores of 9 (6 to 11) at baseline and 8 (5 to 9) at the 12-month follow-up. Overall nonmotor symptom burden, assessed using the Parkinson’s Disease Non-Motor Symptoms Scale (PD-NMSS), likewise remained unchanged, with median scores of 30 (8 to 83) at baseline and 28 (12 to 92) at 12 months. Scores stratified by dose group are presented in Extended Data Table 3.

## Discussion

This phase 1/2 clinical trial involving the bilateral intraputaminal transplantation of the STEM-PD dopaminergic progenitor cell product demonstrates its feasibility with no unexpected safety concerns from the cell product. While the surgery and cell product were generally well tolerated, it should be acknowledged that the adjunctive immunosuppressive treatment used in conjunction with any allogeneic transplantation procedure can come with a risk of opportunistic infections.

A case of disseminated pulmonary aspergillosis infection followed by brain involvement occurred in a participant on active immunosuppression (azathioprine, tacrolimus and corticosteroids) at 10 weeks after transplantation and resulted in a fatal outcome. Invasive aspergillosis is a recognized, albeit rare, complication in immunosuppressed individuals, particularly organ transplant recipients^23^. As the fatality occurred after the last transplantation procedure had been completed, it did not affect the progression of the trial. The study team and Data Safety Monitoring Board (DSMB) performed a critical review of the case, and the steroid taper for the remaining participant was slightly modified.

The immunosuppressive regimen used in the STEM-PD trial was informed by protocols previously used in human fetal VM transplantation studies, including the most recent TransEuro study^14^. These earlier studies demonstrated that such regimens were generally well tolerated and were associated with evidence of robust graft survival, as assessed both at post-mortem^9^ and through in vivo imaging^10,14,24^. In the present study, the immunosuppressive strategy was therefore intentionally designed to prioritize graft survival in a clinical context where the risk of immune-mediated rejection was considered significant, especially given that the STEM-PD product seems similar to human fetal VM tissue immunologically when tested in vitro^25^. Accordingly, a combination of tacrolimus, azathioprine and prednisolone including the addition of basiliximab for induction therapy to reflect contemporary clinical practice in transplantation was used to decrease the risk of graft rejection.

This event underscores the importance of systematically assessing immunosuppression regimes in future allogeneic cell therapy trials. Follow-up PET data from this and similar PD transplantation trials using alternative immunosuppressive regimens^18–20^ will be crucial for identifying the safest and most effective immunosuppression protocol supporting long-term graft survival.

MRI data confirmed safe and accurate delivery of the cell deposits covering the putamen. Device quality may impact not only on target accuracy^14^ but also cell sedimentation rate, cell survival and the reflux rate along the tract. In contrast with previous transplantations using fetal tissue^14,26,27^, the STEM-PD product was delivered bilaterally in one surgical session, thereby avoiding the previous two-staged unilateral operations that require prolonged immunosuppression and come with a potentially increased risk of graft rejection. To reduce confounding effects related to the implantation device, this trial used the Rehncrona–Legradi device previously used in fetal neural tissue transplantation trials in Lund, Sweden^14,26,27^. The choice of delivery device differs among published clinical stem cell trials^18–20^, and it remains unknown whether this represents an important determinant of cell survival and subsequent clinical effects.

Postoperative imaging showed some gliotic changes in a few patients, which is not unexpected and consistent with that seen in DBS surgery^28^ and other cell implantation trials^19^. The rate of occurrence of one minor asymptomatic hemorrhage in a single trajectory (1/80) compares favorably with intracerebral hemorrhage rates reported in DBS literature (estimated at 1.6% per electrode, with ~50% symptomatic)^29^. No other complications occurred with respect to the surgical procedure itself, confirming that the device used in our trial is accurate and safe.

In terms of graft survival, [^18^F]F-DOPA PET imaging indicated areas of focal signal increase in the putamen where the grafts had been placed. We interpret this increase as an indication of dopamine cell survival in at least some of the grafted cell deposits. The areas with increases in PET signal were relatively small, and nongrafted areas did not show any increased [^18^F]F-DOPA retention (for example, caudate nucleus). Reflecting this, the overall putaminal SUVR signal decreased slightly in the low-dose group and increased by 5–10% in the high-dose group. In the nongrafted caudate nucleus, there was, as one would expect, a gradual loss of signal in the range of 3–7%, reflecting the ongoing degenerative nature of the disease. To account for the ongoing neurodegeneration and assuming that the rate of loss of dopamine at this stage of the disease is the same in the caudate and putamen, results are also presented as a putamen-to-caudate ratio, where an increase in the range of 3–5% was found in the low-dose group and 11–15% in the high-dose group.

The putamen-to-caudate ratio has limitations in the transplantation setting because it may overestimate graft-related effects. Increases in putaminal tracer uptake after transplantation may reflect both graft-derived dopaminergic activity and residual host dopaminergic function, while endogenous dopaminergic degeneration may continue during follow-up. Moreover, ratio-based normalization infers similar rates of underlying disease progression in the grafted putamen and the nongrafted caudate, which is suboptimal because caudate and putaminal degeneration may not occur in this way. We therefore also present regional SUVR values for the putamen and caudate separately.

Notably, the clearly observed increase in putaminal signal in the high-dose group supports the presence of a treatment-related signal in this cohort.

It should be noted that the magnitude of the increase of [^18^F]F-DOPA retention at 12 months with STEM-PD cells is lower than that observed in several patients receiving implantation of fetal tissue^24,30,31^. This may suggest that the doses of STEM-PD in this trial were suboptimal and further dose-finding studies may be relevant. Dopamine transporter PET imaging with [^18^F]FE-PE2i did not show significant differences at 12 months, consistent with the dopamine transporter being expressed only late in dopamine neuron maturation.

Further evaluation of the grafts up to 36 months will determine whether the implanted cells continue to grow, mature and reinnervate the putamen after 12 months.

Overall, none of the participants got significantly worse after transplant. Motor scores (MDS-UPDRS part III in OFF) remained relatively stable or showed some improvement (in 60% of participants), and ON-time without troublesome dyskinesias increased especially in the high-dose cohort. Reduction in motor complications was also reflected in reduced OFF time in high-dose participants. Importantly, in contrast to other similar stem cell-derived dopaminergic cell transplantation trials^19^, 85% of participants were able to substantially reduce their dopaminergic medication over the 12-month period compared with baseline, with reductions in the range of 11.9–43.9% in the low-dose group and 17.9–27.4% in the high-dose group.

Reductions in LEDD levels should be interpreted with caution. Medication adjustments are often driven by multiple factors, and reductions in LEDD were not consistently paralleled by improvements in OFF-state motor scores, which showed high variability at the 6–12-month time points. Continued assessment of patients through to the 3-year end point of the trial protocol will be important to determine symptomatic trajectories.

Three phase 1/2 trials in PD investigating human PS cell-derived dopaminergic progenitors have recently been published^18–20^. Those trials, while superficially similar, do critically differ in terms of the manufacturing and dosing of the cell product, the device used to deliver the transplant and the surgical approach taken along with the type of patient recruited and the immunosuppressive regime adopted. In all three trials, safety was shown, with no product-related SAE’s observed. Evidence of dopamine cell survival at the graft site using dopaminergic PET imaging has also been seen in these trials^18–20^, although to a varying extent.

For maximum clinical efficacy, the aim of cell transplantation in PD should probably be to restore putaminal dopaminergic function to normal levels, reducing the need for exogenous dopaminergic therapy. However, the dopaminergic PET signal in putamen, that is, at the graft site indicating graft survival, does not always correlate tightly with clinical benefit. While some studies have demonstrated concordance between [^18^F]F-DOPA-PET signal, graft survival and post-mortem evidence of dopaminergic innervation at the transplant site^9,24^ others have reported normalization of signal without clear clinical benefit^32^. Importantly, evidence from clinical trials with fetal grafts indicates that dopaminergic denervation also in areas not reached by the intraputaminal grafts, such as the ventral striatum, may negatively influence the efficacy of transplantation^33,34^.

In none of the current transplantation trials was the putaminal dopamine PET signal restored to normal at the time points examined, suggesting that further optimization of cell dosing may be required also in these trials^18–20^.

In conclusion, at the 12-month primary end point, no SAEs were attributed to the cell product itself and no signs of tumor formation, abnormal growth or graft-induced OFF dyskinesias were detected. This is in line with safety data reported from other recent trials of transplanted pluripotent-derived dopaminergic cell products^18–20^. The surgical procedure in the STEM-PD trial was largely well tolerated, and accurate cell placement was confirmed in all patients. There were some early indicators of clinical improvement and of cell survival on dopamine PET imaging. No patient had restoration of dopamine levels to normal on PET imaging nor could any of the patients stop their dopaminergic medication, although the large majority of participants required less anti-PD medication at 12 months compared with baseline. The extent to which these transplants confer functional benefit can be reliably assessed only at longer follow-up intervals; accordingly, our trial includes a secondary outcome at 3 years after transplantation.

The STEM-PD trial adds further important confirmatory and complementary evidence to the recently reported feasibility and short-term safety of PS cell-derived dopaminergic progenitor transplantation in PD^18–20^. This is important for this rapidly developing field of PS cell-derived transplantations as a potentially new therapeutic approach for PD. Using a different cell line, manufacturing procedure, dose and implantation method, this European academically led STEM-PD trial provides independent validation of early dopamine graft survival as seen by focal increases in putaminal [^18^F]F-DOPA uptake.

In conclusion, particularly in the context of other recently published trials using human PS cell-derived dopaminergic cell therapies for PD, these findings further support the continued development of this therapeutic approach, including evaluation of the STEM-PD product in larger patient cohorts.

## Methods

### Trial design

This is an open-label, multicenter, first-in-human, single-arm, dose-escalation trial that investigates the safety, tolerability and feasibility of bilateral intraputaminal transplantation of STEM-PD, a dopaminergic neural progenitor cell product derived from the clinical-grade human ES cell line RC17.

The primary end point was the number and nature of AEs and SAEs occurring within the first 12 months following transplantation, along with the absence of space-occupying lesions on cranial MRI during the same period. All AEs were graded according to the Common Terminology Criteria for Adverse Events (CTCAE) and coded using the Medical Dictionary for Regulatory Activities (MedDRA).

AEs of special interest were prospectively defined as:

- Stroke
- Major intracranial hemorrhage
- Central nervous system infection
- Major or rapid worsening of PD symptoms or existing dyskinesias
- Clinically significant events potentially related to immunosuppression (for example, opportunistic infections; malignancies including squamous cell carcinoma, Kaposi sarcoma, and lymphomas; or metabolic complications such as osteoporosis or diabetes)
- Any unexpected event occurring within 24 h after transplantation (excluding common perioperative symptoms such as nausea or malaise)

Premature termination of the trial could be initiated by the investigators or the DSMB owing to safety or ethical concerns. A temporary pause in transplantation was mandated if one participant experienced an SAE that was considered possibly, probably or definitely related to the cell product or if two participants experienced severe adverse reactions related to the product.

Secondary end points, to be assessed at 36 months, include changes in motor features in the OFF-medication state and adjustments in antiparkinsonian therapy. In addition, cell survival and functional integration will be evaluated using [^18^F]F-Dopa and dopamine transporter PET imaging (FE-PE2i), alongside ongoing monitoring of AEs and SAEs from 12 to 36 months.

Exploratory outcomes include additional PET imaging (6, 12 and 24 months), wearable motor assessments, anti-HLA class I antibody development, cerebrospinal fluid inflammatory biomarkers and responses to an L-dopa challenge.

The relevant 12-month data are reported in this paper. Full end point descriptions are presented in Extended Data Table 4.

### Trial oversight

The study received ethical approval from the Swedish Ethical Review Authority (EPM Dnr 2021-06945-01) and the South Central – Oxford A Research Ethics Committee (ref no. 23/SC/0243). Regulatory approval was granted by the Swedish Medical Products Agency (Dnr 5.1-2022-57953) and the UK Medicines and Healthcare products Regulatory Agency (CTA 40773/0001/001-0001). The study transitioned to EU Clinical Trial Regulation 536/2014 with updated approval from the Swedish MPA (Dnr 5.1.1-2024-100773). Oversight is provided by an independent DSMB and a TMG. Trial registration: EudraCT 2021-001366-38; EU trial no. 2024-511237-35-00; ClinicalTrials.gov: NCT05635409.

### Participants

Participants were recruited from two sites: Skåne University Hospital (Lund, Sweden) and Cambridge University Hospital (UK), with all surgical procedures performed at Skåne University Hospital. Recruitment occurred through a long-term observational cohort study (Transeuro observational study) with harmonized clinical assessments^14,15^ allowing for potential individual baseline trajectory estimation and minimizing test–retest variability.

Eight patients with moderately advanced PD were enrolled on the basis of predefined inclusion and exclusion criteria independent of sex or gender (Fig. 1a). Sex or gender was self-reported and not considered in the study design. Written informed consent was obtained from all participants before screening. Participants received compensation for travel expenses only. In brief, participants aged between 50 and 75 years, diagnosed with PD and Hoehn–Yahr stage ≤3 in the OFF state and a disease duration of >5 years were deemed eligible for enrollment. Significant drug-induced dyskinesias as defined by a score of >2 in any body part in the ON state (AIMS), significant cognitive impairment consistent with MoCA score of ≤24 and ongoing major medical or psychiatric disorders, including depression (MADRS >20) were considered exclusion criteria (see Extended Data Table 5 for detailed inclusion and exclusion criteria).

Recruitment occurred over 18 months in a predetermined staggered fashion with mandated safety intervals. A 1-month interval followed transplantation of participant 1; a 3-month interval followed participant 2; and after participant 4, a 6-month pause preceded dose escalation. Safety intervals for the high dose were followed as for the low dose. All participants will be followed for 36 months after transplantation, with plans for lifelong follow-up under a separate protocol.

### Intervention

#### Cell product

STEM-PD consists of human ES cell-derived dopaminergic progenitor cells (human ES cell line RC17), previously validated in preclinical studies for safety, potency and efficacy^21^. Clinical-grade cell batches were manufactured under good manufacturing practice (GMP) at the Royal Free Hospital (London, UK), banked and shipped to Skåne University Hospital under controlled conditions in vapor-phase liquid nitrogen. On the day of transplantation, cryopreserved vials were thawed aseptically, washed twice and resuspended into transplantation vehicle (Hank’s Balanced Salt Solution (HBSS) with 20 U ml^−1^ Pulmozyme) to achieve a target cell concentration of around 70,000 cells μl^−1^. On resuspension, the cell suspension was assessed for viability and cell number. Acceptance criteria for release to transplantation were ≥80% live cells and a concentration of live cells between 50,000 and 90,000 cells μl^−1^. All cell suspensions passed the acceptance criteria, with an average yield of 76,843 live cells μl^−1^ across the eight transplanted cell suspensions. Samples of wash medium were tested for sterility under GMP through 14-day inoculation in aerobic and anaerobic conditions (performed at ALS Sollentuna Mikrolab). All samples were confirmed to be sterile. The final cell suspension was stored in a temperature-controlled container (TRU iBox R11, Sofigram) and transferred to the surgery room where it was kept at 2–8 °C during the duration of the surgery.

#### Dosing

Two sequential doses were evaluated. The low dose (3.54 × 10^6^ cells per putamen) was intended to yield approximately 100,000 surviving dopaminergic neurons, while the high dose (7.08 × 10^6^ cells) aimed at yielding 200,000 dopaminergic neurons per putamen. Participants were sequentially assigned in two cohorts (*n* = 4 per dose). For both doses, the concentration of cells in the cell suspension was 50,000–90,000 cells μl^−1^. Dose escalation was approved following DSMB and TMG review of 6-month safety and evaluation of the [^18^F]F-DOPA- PET data in cohort 1.

#### Surgical procedure

A preoperative high-resolution planning cranial 3-T MRI scan (MAGNETOM Vida, Siemens Healthineers) and a high-resolution photon-counting detector computed tomography angiography (PCD–CTA; Siemens Naeotom Alpha) were performed preoperatively to optimize planning of targets and to visualize localization of vessels. All grafts were delivered using a frame-based stereotactic technique (Elekta stereotactic Leksell G-frame) under general anesthesia. To minimize brain shift, participants were operated in the neutral, supine position as placed in the MRI scanner. This position minimizes cell sedimentation in the needle (outer diameter of transplantation cannula was 1,0 mm and the inner diameter 0,8 mm). Cells were administered using the non-CE-marked Rehncrona/Legradi microinjection device that has been used in previous clinical fetal neural tissue transplantation trials^14^ along five tracts for each putamen (Fig. 1b), (one frontal to the anterior commissure, one at the anterior commissural plane and three posterior to the anterior commissure). Bilateral trajectories into the putamen were established through one or more burr holes on each side, avoiding the ventricles and blood vessels. Trajectories and deposits were placed to achieve maximal distribution of the cell suspension within the putamen. Loading of the device took place immediately before the implantation to each of the five tracts per hemisphere. Each time, the cell product was carefully pipetted to ensure even distribution of the cells during loading. Either four (low dose) or eight deposits (high dose) were placed in each tract containing 2.5 µl cell suspension/deposit administered with an approximate infusion rate of 5.0 µl min^−1^. The device was rotated during deposition of the cells to reduce sedimentation of cells in the needle. There was a defined waiting time of 1 min after each deposit to reduce cell reflux before the needle was moved to the next position for the following deposit. After completion of each trajectory, the needle was left in place for another 5 min before being slowly withdrawn. The total duration of surgery, including fitting of the stereotaxic frame, was up to 12 h, of which approximately 9 h were spent under general anesthesia.

#### Immunosuppression

Given the allogeneic nature of STEM-PD, all participants received triple immunosuppressive therapy for 12 months, modeled after standard organ transplantation protocols^35,36^. Immunosuppression began 1 day before surgery and was tapered off over months 12–15. The regimen included methylprednisolone (1 g, intravenously, i.v.) at surgery; basilixumab (20 mg i.v. on days 0 and 4); tacrolimus (0.05 mg kg^−1^ twice per day orally, targeting 7 ng ml^−1^ during month 1 and 5 ng ml^−1^ thereafter); azathioprine (1 mg kg^−1^ per day orally) and oral prednisolone (starting at 40 mg, tapered to 10 mg by week 12, maintained at 10 mg until week 52 and discontinued by week 62).

Routine monitoring included plasma tacrolimus levels, liver, renal and hematological blood chemistry measurements and surveillance for adverse effects. Prophylactic antibiotics, osteoporosis prevention (alendronic acid and calcium/vitamin D) and gastric protection were administered throughout the period of immunosuppression.

### Assessment schedule

Participants underwent detailed screening, including imaging (MRI, [^18^F]F-DOPA-PET), blood tests, MDS-UPDRS III (ON and OFF states), AIMS tests and cognitive/psychiatric evaluations. Baseline assessments included [^18^F]FE-PE2i-PET, an L-dopa challenge, Hauser diary and extended cognitive testing^37^, psychiatric scales (MADRS), and quality of life instruments (Parkinson’s Disease Questionaire-39, EQ-5D-5L, PD-NMSS). Dyskinesias were assessed before and after grafting in the ON and the OFF state applying AIMS and Rush dyskinesia rating scales.

Safety was assessed via AE reporting, clinical examinations, laboratory tests and serial cranial MRI. Participants were admitted 1 day preoperatively and hospitalized for 4 days. Postoperative follow-up occurred on days 7, 14, 21, 28 and 42 and then monthly until month 6 and every 3 months thereafter (Fig. 1c). Clinical evaluations were repeated at 6, 9 and 12 months and will be continued at 18, 24, 30 and 36 months including both ON and OFF-state evaluations at 6, 12, 24 and 36 months. The schedule is shown in Fig. 1c and detailed in the published study protocol^37^.

There were no specific requirements in the trial protocol to keep dopaminergic medication unchanged. All non-trial-related medication was managed at the discretion of the respective trial investigator in response to clinical need.

### Imaging

Cranial MRI scans were acquired on a 3-T MAGNETOM Prisma scanner (Siemens Healthineers) using the following sequences: T1-weighted magnetization-prepared rapid acquisition with gradient echo (MPRAGE, axial), T2-weighted fluid-attenuated inversion recovery (FLAIR, axial), T2-weighted turbo spin echo (coronal), diffusion-weighted imaging (axial) and susceptibility-weighted imaging (axial). Safety MRIs were performed 2–3 days after transplantation and at months 1, 2, 3, 6, 9 and 12 and will also be performed at months 24 and 36 (Fig. 1c).

[^18^F]F-DOPA- PET was performed at screening, 6 and 12 months and will be performed at 24 and 36 months, whereas [^18^F]FE-PE2i-PET is scheduled at baseline, 12, 24 and 36 months. Imaging was performed off L-dopa treatment. At 1 h before the start of the [^18^F]F-DOPA scan, 150 mg carbidopa was administered. PET scanning was performed on two identical GE Discovery MI cameras and started with the injection of ~185 MBq [^18^F]F-DOPA. Data were collected in LIST mode for 90 min and binned into 48 time frames (12 × 10 s, 6 × 20 s, 6 × 30 s, 3 × 60 s, 5 × 120 s and 14 × 300 s) and reconstructed using VPFX-S (ordered subset expectation maximization (OSEM) with 6 iterations using 17 subsets, time-of-flight correction and point-spread-function/Sharp IR). Images were smoothed in plane using a 3-mm gaussian filter and with a standard *Z* filter. Reconstructed images were further processed using an in-house-developed pipeline described previously^38^. In brief, frames were motion corrected and summed and the average image was coregistered to structural t1-MPRAGE images. The MRIs were brain extracted using data from T1-MPRAGE and T2-FLAIR sequences and segmented into gray and white matter. The images were further normalized to Montreal Neurological Institute (MNI) 152 standard space. Regions of interest, defined using the Desikan–Killiany atlas in FreeSurfer version 6.0, were warped back from MNI space to individual space using the reverse transform from the normalization and applied to the PET images. Occipital cortex, used as a reference region, was defined as a volume weighted average of bilateral lateral occipital, lingual, cuneus and pericalcarine cortices. SUVRs were calculated using the 70–90-min time span using the occipital cortex as a reference region.

### Statistical analysis

This interim analysis includes descriptive safety data through 12 months for all participants receiving either dose of STEM-PD, as of 14 October 2025. Owingto the small sample size, no formal statistical comparisons were performed and, in this case, data are generally presented as median (range).

PET images were analyzed over all patients using a paired Wilcoxons signed-rank test implemented in R version 4.2.3, using packages ‘broom’, ‘purr’, ‘tidyr’ and ‘dplyr’, and PET quantification graphs were produced using ‘ggplot2’ and ‘patchwork’.

### Data management and monitoring

All trial data were collected and managed using a validated electronic case report form. Monitoring was conducted per Good Clinical Practice standards, and source data verification was performed on site and remotely.

### Reporting summary

Further information on research design is available in the Nature Portfolio Reporting Summary linked to this article.

## Online content

Any methods, additional references, Nature Portfolio reporting summaries, source data, extended data, supplementary information, acknowledgements, peer review information; details of author contributions and competing interests; and statements of data and code availability are available at https://doi.org/10.1038/s41591-026-04525-0.

## Supplementary information

Supplementary Information Study protocol and statistical analysis plan.

Reporting Summary

## Source data

Source Data Fig. 2 Source data for Fig. 2 (^18^F-Dopa-PET).

Source Data Fig. 3 Source data for Fig. 3 (Preliminary efficacy outcomes).

Source data Extended Data Fig. 1 Source data for Extended Data Fig. 1 (^18^F)FE-PE2I-PET.

Source Data Extended Data Fig. 2 Source Data Extended Data Fig. 2.

## Figures and Tables

**Extended Data Table 1:**
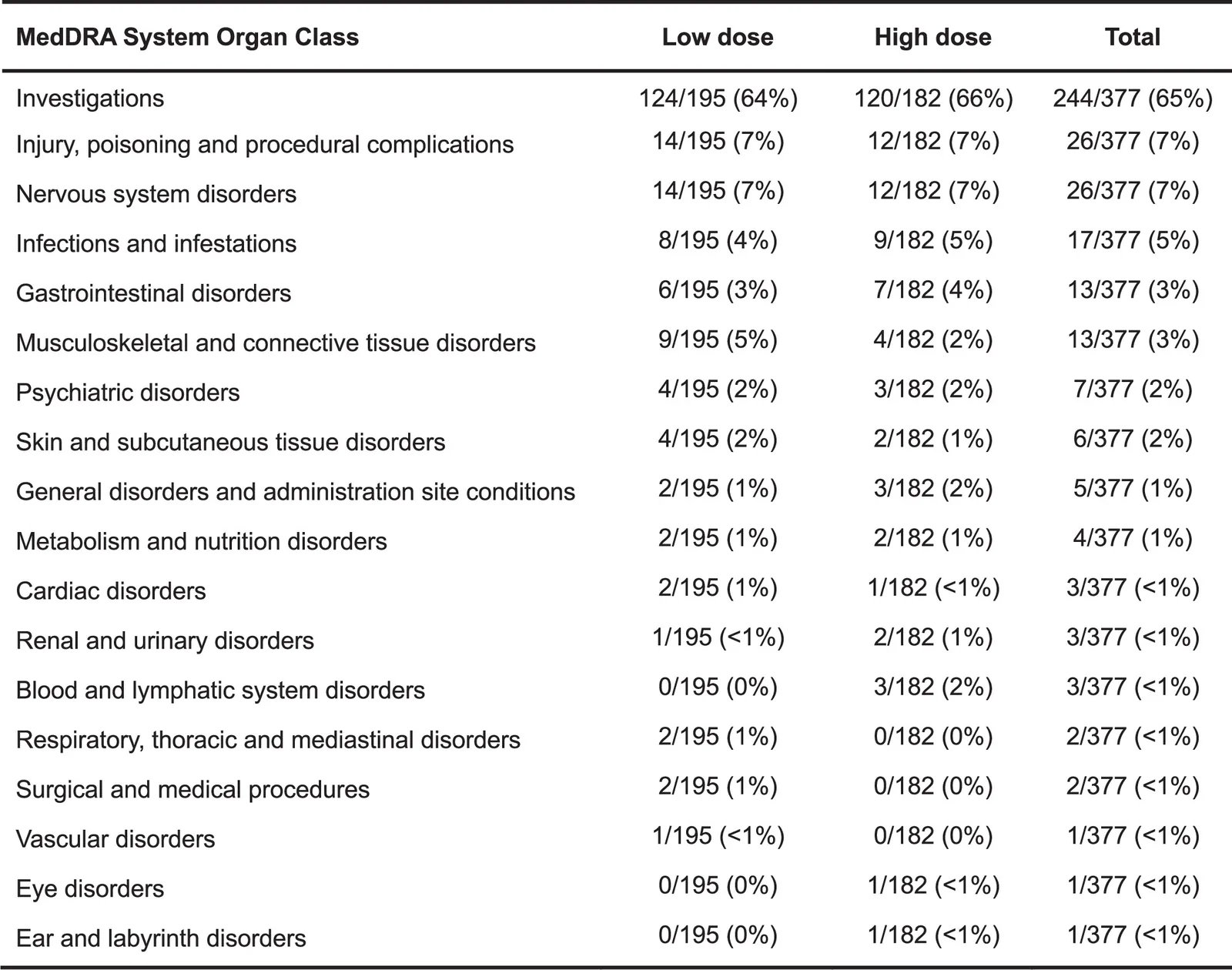
Adverse Events according to MedDRA System Organ Class

**Extended Data Table 2:**
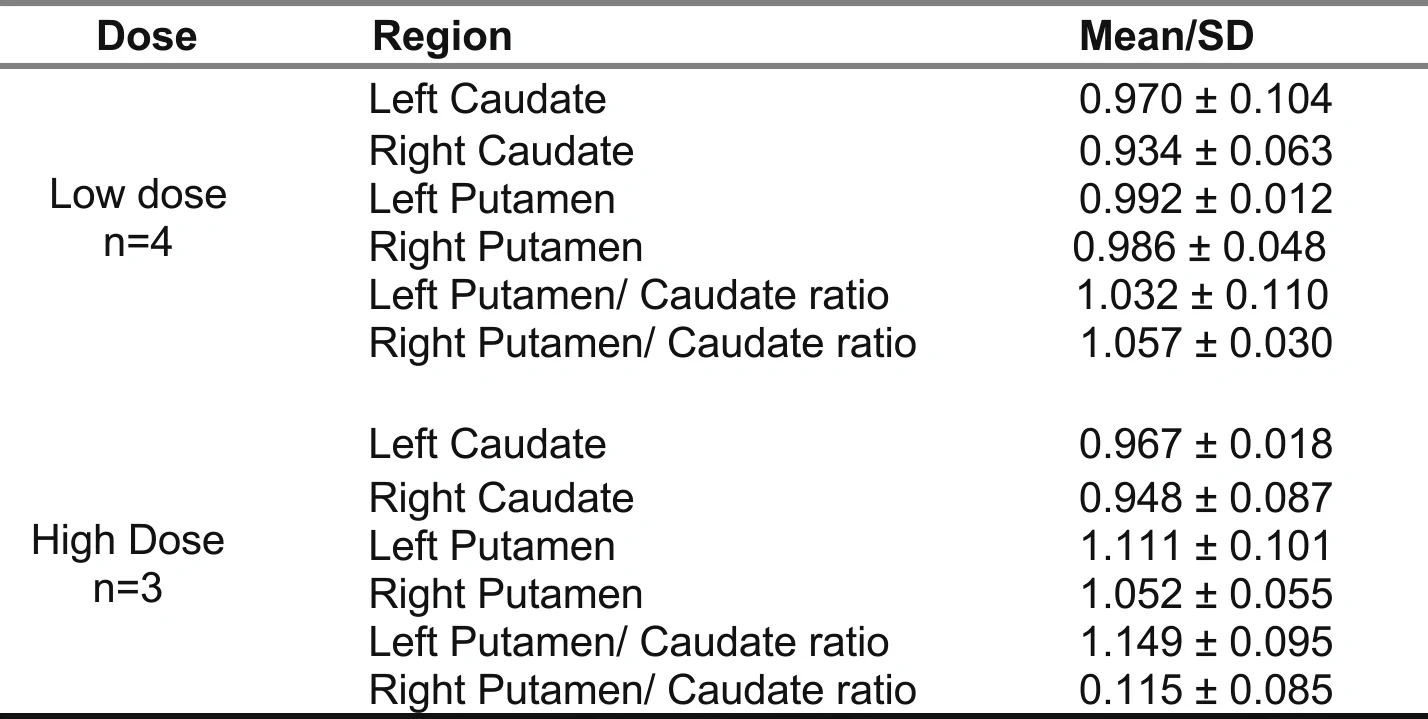
Putamen and caudate [18 F]F-Dopa standardized uptake value ratios (SUVRs) at 12 months relative to baseline levels

**Extended Data Table 3:**
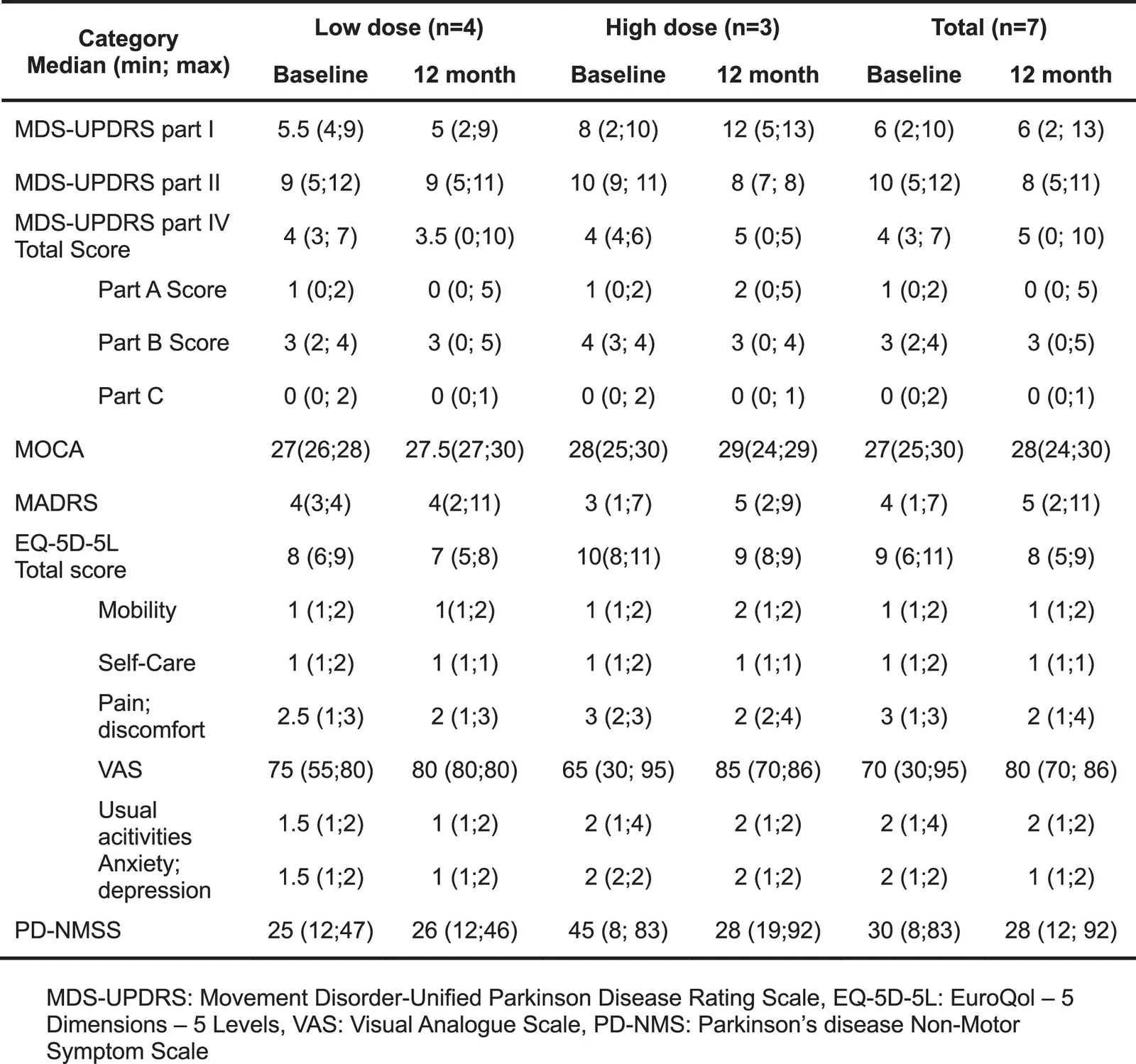
Clinical non-motor outcomes

**Extended Data Table 4:**
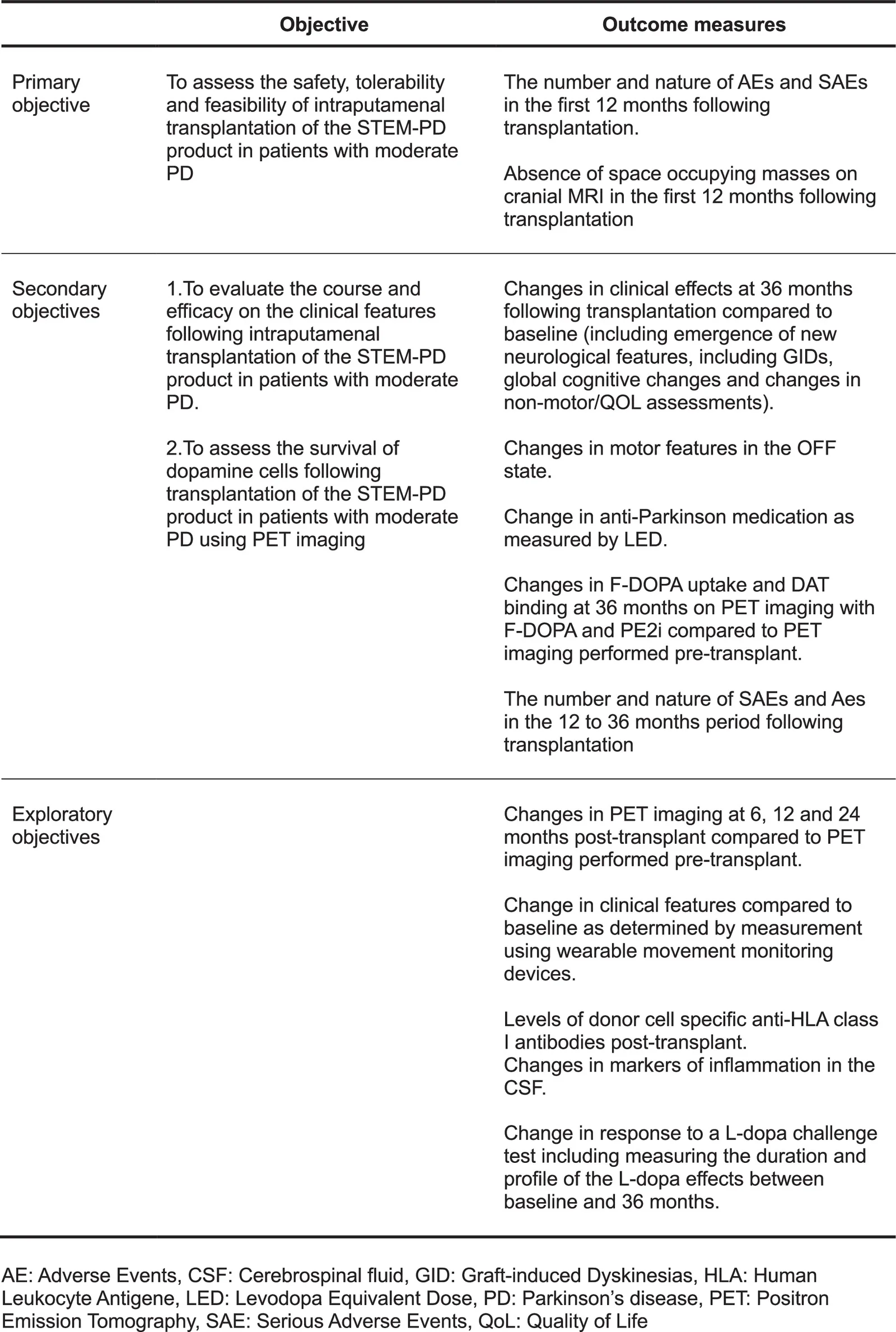
Study objectives and outcome measure

**Extended Data Table 5:**
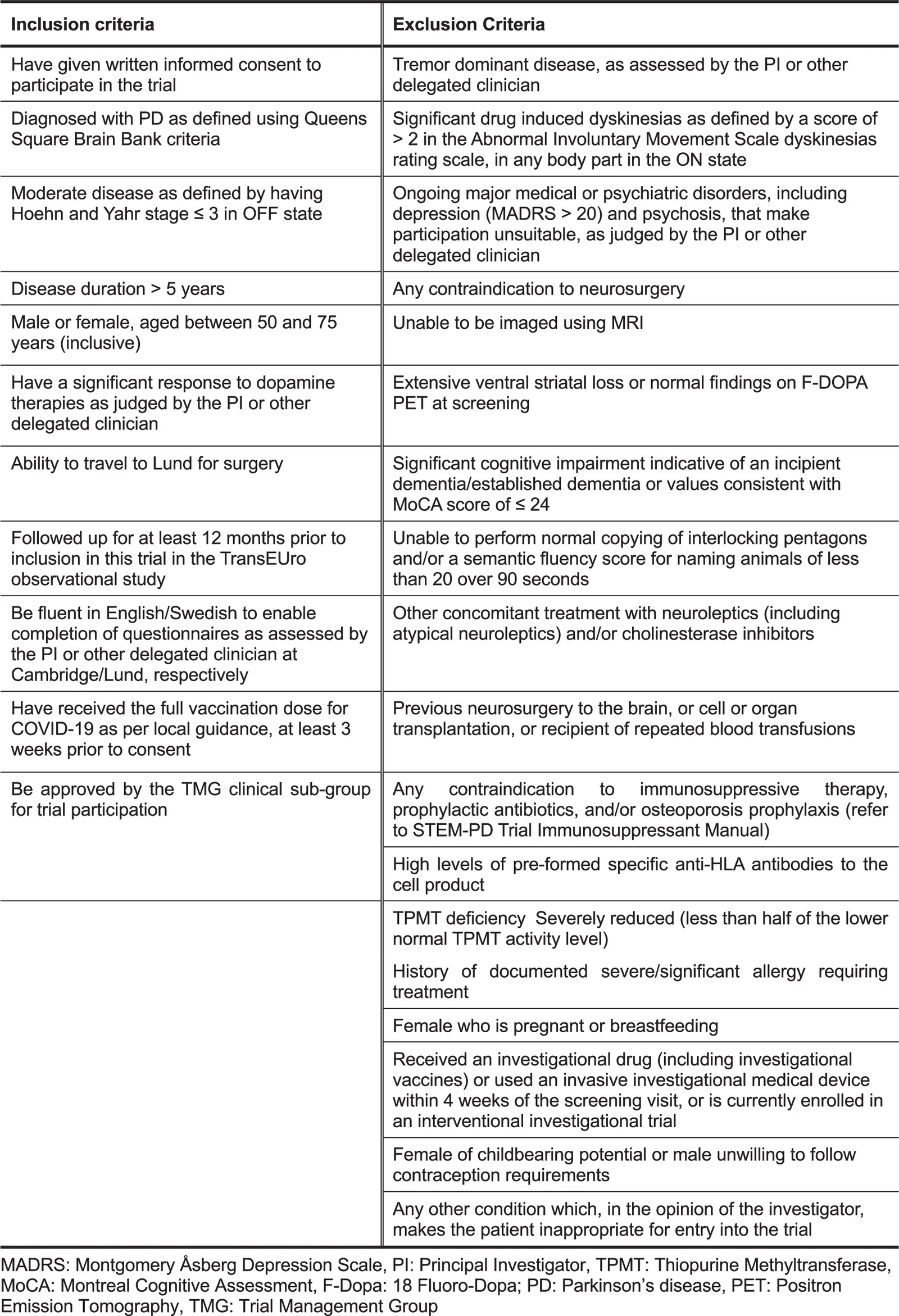
Eligibility criteria

## Data Availability

The data that support the findings of this study are available within the Article, Extended Data Tables 1–5 and Extended Data Figs. 1 and 2, including Source Data. Additional de-identified participant-level data underlying the results reported in this Article may be made available to qualified researchers on reasonable request, subject to applicable ethical approvals, data protection regulations, participant consent and approval by the study sponsor and relevant institutional data governance bodies. As this is a small first-in-human clinical trial of an advanced therapy medicinal product, individual-level data will not be made publicly available owing to the potential risk of participant re-identification. Requests should include a scientifically sound proposal, a statistical analysis plan and evidence of appropriate ethical and data protection approvals where applicable. Requests will be evaluated by the corresponding author, the study sponsor and relevant institutional representatives. Approved requests will require a data transfer or data use agreement specifying permitted analyses, data security requirements, confidentiality obligations and restrictions on onward sharing. Requests should be directed to Gesine Paul, gesine.paul-visse@med.lu.se. The anticipated response time is within 8–12 weeks of receipt of a complete request. De-identified participant-level data will be available 6 months after publication and for 5 years after publication. The study protocol is available in ref. ^37^ and as Supplementary Note 1. The clinical trial is registered at ClinicalTrials.gov, NCT05635409, and EudraCT 2021-001366-38. Source data are provided with this paper.

## Code availability

No custom code or algorithm central to the conclusions of this study was developed. Statistical analyses were performed using standard software packages as described in the Methods. Any analysis scripts required to reproduce the reported aggregate analyses are available from the corresponding author on reasonable request, subject to institutional approval.

## References

[1] Collaborators, G. B. D. N. Global, regional, and national burden of neurological disorders, 1990–2016: a systematic analysis for the Global Burden of Disease Study 2016. *Lancet Neurol.* **18**, 459–480 (2019). 30879893 10.1016/S1474-4422(18)30499-X PMC6459001

[2] Dorsey, E. R., Sherer, T., Okun, M. S. & Bloem, B. R. The emerging evidence of the Parkinson pandemic. *J. Parkinsons Dis.* **8**, S3–S8 (2018). 30584159 10.3233/JPD-181474 PMC6311367

[3] Bloem, B. R., Okun, M. S. & Klein, C. Parkinson’s disease. *Lancet* **397**, 2284–2303 (2021). 33848468 10.1016/S0140-6736(21)00218-X

[4] Morris, H. R., Spillantini, M. G., Sue, C. M. & Williams-Gray, C. H. The pathogenesis of Parkinson’s disease. *Lancet* **403**, 293–304 (2024). 38245249 10.1016/S0140-6736(23)01478-2

[5] Dauer, W. & Przedborski, S. Parkinson’s disease: mechanisms and models. *Neuron* **39**, 889–909 (2003). 12971891 10.1016/s0896-6273(03)00568-3

[6] Foltynie, T. et al. Medical, surgical, and physical treatments for Parkinson’s disease. *Lancet* **403**, 305–324 (2024). 38245250 10.1016/S0140-6736(23)01429-0

[7] Verhagen Metman, L., Monje, M. H. G., Obeso, J. A. & Martinez-Fernandez, R. Focused ultrasound therapy: back to the future. *Parkinsonism Relat. Disord.* **121**, 106023 (2024). 38320923 10.1016/j.parkreldis.2024.106023

[8] Armstrong, M. J. & Okun, M. S. Choosing a Parkinson disease treatment. *J. Am. Med. Assoc.* **323**, 1420 (2020). 10.1001/jama.2020.1224 32286645

[9] Li, W. et al. Extensive graft-derived dopaminergic innervation is maintained 24 years after transplantation in the degenerating parkinsonian brain. *Proc. Natl Acad. Sci. USA* **113**, 6544–6549 (2016). 27140603 10.1073/pnas.1605245113 PMC4988567

[10] Kefalopoulou, Z. et al. Long-term clinical outcome of fetal cell transplantation for Parkinson disease: two case reports. *JAMA Neurol.* **71**, 83–87 (2014). 24217017 10.1001/jamaneurol.2013.4749 PMC4235249

[11] Barker, R. A., Barrett, J., Mason, S. L. & Bjorklund, A. Fetal dopaminergic transplantation trials and the future of neural grafting in Parkinson’s disease. *Lancet Neurol.* **12**, 84–91 (2013). 23237903 10.1016/S1474-4422(12)70295-8

[12] Freed, C. R. et al. Transplantation of embryonic dopamine neurons for severe Parkinson’s disease. *N. Engl. J. Med.* **344**, 710–719 (2001). 11236774 10.1056/NEJM200103083441002

[13] Olanow, C. W. et al. A double-blind controlled trial of bilateral fetal nigral transplantation in Parkinson’s disease. *Ann. Neurol.* **54**, 403–414 (2003). 12953276 10.1002/ana.10720

[14] Barker, R. A. et al. The TransEuro open-label trial of human fetal ventral mesencephalic transplantation in patients with moderate Parkinson’s disease. *Nat. Biotechnol.* **44**, 70–78 (2025). 10.1038/s41587-025-02567-2 PMC12807853 40316701

[15] Barker, R. A. Designing stem-cell-based dopamine cell replacement trials for Parkinson’s disease. *Nat. Med.* **25**, 1045–1053 (2019). 31263283 10.1038/s41591-019-0507-2

[16] Paul, G., Morizane, A., Kirkeby, A., Takahashi, J. & Henchcliffe, C. in *International Review of Movement Disorders: Device-Aided Therapies in Parkinson’s Diseas**e* (eds Odin, P. et al.) Vol. 7, 191–220 (Elsevier, 2024).

[17] Kirkeby, A., Main, H. & Carpenter, M. Pluripotent stem-cell-derived therapies in clinical trial: a 2025 update. *Cell Stem Cell* **32**, 10–37 (2025). 39753110 10.1016/j.stem.2024.12.005

[18] Sawamoto, N. et al. Phase I/II trial of iPS-cell-derived dopaminergic cells for Parkinson’s disease. *Nature* **641**, 971–977 (2025). 40240591 10.1038/s41586-025-08700-0 PMC12095070

[19] Tabar, V. et al. Phase I trial of hES cell-derived dopaminergic neurons for Parkinson’s disease. *Nature* **641**, 978–983 (2025). 40240592 10.1038/s41586-025-08845-y PMC12095069

[20] Chang, J. W.et al. Phase 1/2a clinical trial of hESC-derived dopamine progenitors in Parkinson’s disease. *Cell* **188**, 7036–7048(2025). 41086804 10.1016/j.cell.2025.09.010

[21] Kirkeby, A. et al. Preclinical quality, safety, and efficacy of a human embryonic stem cell-derived product for the treatment of Parkinson’s disease, STEM-PD. *Cell Stem Cell* **30**, 1299–1314(2023). 37802036 10.1016/j.stem.2023.08.014

[22] Tomlinson, C. L. et al. Systematic review of levodopa dose equivalency reporting in Parkinson’s disease. *Mov. Disord.* **25**, 2649–2653 (2010). 21069833 10.1002/mds.23429

[23] Kasper, E. M. et al. Post-transplant aspergillosis and the role of combined neurosurgical and antifungal therapies under belatacept immunosuppression. *Surg. Neurol. Int.* **2**, 75 (2011). 21748028 10.4103/2152-7806.81969 PMC3130373

[24] Piccini, P. et al. Dopamine release from nigral transplants visualized in vivo in a Parkinson’s patient. *Nat. Neurosci.* **2**, 1137–1140 (1999). 10570493 10.1038/16060

[25] Curle, A. J. et al. The immunological profile of RC17 hESC-derived dopaminergic neural progenitor cells in vitro: implications for the STEM-PD clinical trial. *Stem Cell Rep.* **21**, 102818 (2026). 10.1016/j.stemcr.2026.102818 PMC12985363 41687623

[26] Lindvall, O. et al. Human fetal dopamine neurons grafted into the striatum in two patients with severe Parkinson’s disease. A detailed account of methodology and a 6-month follow-up. *Arch. Neurol.* **46**, 615–631 (1989). 2786405 10.1001/archneur.1989.00520420033021

[27] Lindvall, O. et al. Grafts of fetal dopamine neurons survive and improve motor function in Parkinson’s disease. *Science* **247**, 574–577 (1990). 2105529 10.1126/science.2105529

[28] Erasmi, R. et al. White matter changes along the electrode lead in patients treated with deep brain stimulation. *Front. Neurol.* **9**, 983 (2018). 30519212 10.3389/fneur.2018.00983 PMC6259286

[29] Cheyuo, C. et al. Comprehensive characterization of intracranial hemorrhage in deep brain stimulation: a systematic review of literature from 1987 to 2023. *J. Neurosurg.* **141**, 381–393 (2024). 38518284 10.3171/2024.1.JNS232385

[30] Brundin, P. et al. Bilateral caudate and putamen grafts of embryonic mesencephalic tissue treated with lazaroids in Parkinson’s disease. *Brain* **123**, 1380–1390 (2000). 10869050 10.1093/brain/123.7.1380

[31] Hagell, P. et al. Sequential bilateral transplantation in Parkinson’s disease: effects of the second graft. *Brain* **122**, 1121–1132 (1999). 10356064 10.1093/brain/122.6.1121

[32] Kordower, J. H. et al. Robust graft survival and normalized dopaminergic innervation do not obligate recovery in a Parkinson disease patient. *Ann. Neurol.* **81**, 46–57 (2017). 27900791 10.1002/ana.24820 PMC5890810

[33] Piccini, P. et al. Factors affecting the clinical outcome after neural transplantation in Parkinson’s disease. *Brain* **128**, 2977–2986 (2005). 16246865 10.1093/brain/awh649

[34] Ma, Y. et al. Dopamine cell implantation in Parkinson’s disease: long-term clinical and ^18^F-FDOPA PET outcomes. *J. Nucl. Med.* **51**, 7–15 (2010). 20008998 10.2967/jnumed.109.066811 PMC2946843

[35] Nelson, J. et al. Consensus recommendations for use of maintenance immunosuppression in solid organ transplantation: endorsed by the American College of Clinical Pharmacy, American Society of Transplantation, and the International Society for Heart and Lung Transplantation. *Pharmacotherapy* **42**, 599–633 (2022). 36032031 10.1002/phar.2716

[36] Nelson, J. et al. Consensus recommendations for use of maintenance immunosuppression in solid organ transplantation: endorsed by the American College of Clinical Pharmacy, American Society of Transplantation, and International Society for Heart and Lung Transplantation: an executive summary. *Pharmacotherapy* **42**, 594–598 (2022). 35810342 10.1002/phar.2718

[37] Paul, G. et al. STEM-PD trial protocol: a multi-centre, single-arm, first-in-human, dose-escalation trial, investigating the safety and tolerability of intraputamenal transplantation of human embryonic stem cell-derived dopaminergic cells for Parkinson’s disease. *BMJ Open* **15**, e107597 (2025). 41469040 10.1136/bmjopen-2025-107597 PMC12766818

[38] Leuzy, A. et al. Validation of a spatial normalization method using a principal component derived adaptive template for [^18^F]florbetaben PET. *Am. J. Nucl. Med. Mol. Imaging.* **10**, 161–167 (2020). 32929394 PMC7486549

