# Supplementary material for: Human embryonic stem cell-derived dopaminergic cells for Parkinson’s disease: a phase 1/2 open-label trial
Source: Nat Med. 2026 Jul 9;32(9):3449–57. doi: 10.1038/s41591-026-04525-0 (PMC13577911; doi:10.1038/s41591-026-04525-0)
Supplement: Supplementary file 1 [file 41591_2026_4525_MOESM1_ESM.pdf]

nature medicine

Supplementary information https://doi.org/10.1038/s41591-026-04525-0

Human embryonic stem cell-derived dopaminergic cells for Parkinson's disease: a phase 1/2 open-label trial

In the format provided by the authors and unedited

Clinical Trial Protocol

Trial Title	STEM-PD trial: A multicentre, single arm, first in human, dose-escalation trial, investigating the safety and tolerability of intraputamenal transplantation of human embryonic stem cell derived dopaminergic cells for Parkinson's disease (STEM-PD product)
Trial Acronym	STEM-PD trial
Protocol Number:	CCTU0263
EudraCT Number:	2021-001366-38
ClinicalTrials.gov number:	NCT05635409
Investigational Medicinal Product	Human embryonic stem cell (hESC) derived dopaminergic cells (STEM-PD product)
Protocol Version/Date:	4.2 (dated 25 Jul 2024)

Protocol Author/Clinical Lead:	Professor Roger Barker
Protocol Author/Clinical Lead Address:	John van Geest Centre for Brain Repair, Forvie Site, Robinson Way, Cambridge, CB2 0PY, England, UK
Telephone:	+44 (0) 1223 331184
Trial Sponsor:	Region Skåne – Skåne University Hospital
Safety Reporting (including AESI, SAE and SUSAR)	STEM-PD trial coordinator<br>Bronwen Harry<br>Email: cuh.stempd_trial@nhs.net

1 Protocol Signature Pages

1.1 Sponsor signature

On behalf of the Sponsor organisation, I confirm that this protocol includes all essential information to be able to conduct this trial. On behalf of the Sponsor organisation, I will submit the protocol and all other important trial-related information to the responsible investigator(s) so that they can conduct the trial correctly. I am aware that it is the Sponsor responsibility to ensure the staff members who work with this trial are informed and trained.

Name: Håkan Widner (on behalf of Region Skåne – Skåne University Hospital)

Signature:

Date: 2024 August [illegible]

1.2 Investigator signatures

1.2.1 Protocol Author, Clinical Lead and Principal Investigator - Cambridge University Hospitals NHS Foundation Trust (Cambridge, UK)

I have read this protocol and agree that it includes all essential information to be able to conduct the trial. By signing my name below, I agree to conduct the trial in compliance with this protocol, the Declaration of Helsinki, the principles of ICH GCP (Good Clinical Practice) and the current national and international regulations governing the conduct of this clinical trial.

I will submit this protocol and all other important trial-related information to the staff members and investigators who participate in this trial, so that they can conduct the trial correctly. I am aware of my responsibility to continuously keep the staff members and investigators who work with this trial informed and trained.

I am aware that quality control of this trial will be performed in the form of monitoring, audit, and possibly inspection.

Name: Roger Barker

Signature: ______________________

Date: 26. July 2024

1.2.2 Principal Investigator - Region Skåne – Skåne University Hospital (Lund, Sweden)

I have read this protocol and agree that it includes all essential information to be able to conduct the trial. By signing my name below, I agree to conduct the trial in compliance with this protocol, the Declaration of Helsinki, the principles of ICH GCP (Good Clinical Practice) and the current national and international regulations governing the conduct of this clinical trial.

I will submit this protocol and all other important trial-related information to the staff members and investigators who participate in this trial, so that they can conduct the trial correctly. I am aware of my responsibility to continuously keep the staff members and investigators who work with this trial informed and trained.

I am aware that quality control of this trial will be performed in the form of monitoring, audit, and possibly inspection.

Name: Gesine Paul-Visse

Signature: ________________________________

Date: ______6 august 2024______________________

2 Trial Management Committee(s) and Protocol Contributors

Agnete Kirkeby<br>(*TMG*)	Associate Senior Lecturer, Lund University<br>agnete.kirkey@med.lu.se
Anders Björklund	Professor of Histology, Lund University<br>anders.bjorklund@med.lu.se
Bronwen Harry<br>(*TMG*)	STEM-PD Trial Coordinator, University of Cambridge<br>beh38@cam.ac.uk
Emma Cutting<br>(*TMG*)	Senior Clinical Trials Coordinator, University of Cambridge<br>evc26@cam.ac.uk
Gesine Paul-Visse<br>Principal Investigator – RS-SUS Lund<br>(*TMG, clinical sub-group*)	Professor of Neurology, Lund University and Consultant Neurologist, Region Skåne – Skåne University Hospital<br>gesine.paul-visse@med.lu.se
Håkan Widner<br>Sponsor/Investigator – RS-SUS Lund<br>(*TMG, clinical sub-group*)	Professor of Neurology, Lund University and Consultant Neurologist, Region Skåne – Skåne University Hospital<br>hakan.widner@med.lu.se
Hjálmar Bjartmarz<br>Co-Investigator – RS-SUS Lund<br>(*TMG, clinical sub-group*)	Consultant Neurosurgeon, Region Skåne – Skåne University Hospital<br>hjalmar.bjartmarz@med.lu.se
Jenny Nelander<br>(*TMG*)	Assistant Researcher, Lund University<br>jenny.nelander@med.lu.se
Julie Warner	Vice President, Regulatory Affairs, Boyd Consultants<br>julie.warner@boydconsultants.com
Kim Champion	Manager, Regulatory Affairs, Boyd Consultants<br>kim.champion@boydconsultants.com
Malin Parmar<br>(*TMG, clinical sub-group*)	Professor of Cellular Neuroscience, Lund University<br>malin.parmar@med.lu.se
Nicholas Lao-Kaim<br>(*TMG, clinical sub-group*)	Postdoctoral Research Associate, Imperial College London<br>n.kaim@imperial.ac.uk
Olle Lindvall	Professor of Neurology, Lund University<br>olle.lindvall@med.lu.se
Oskar Hansson<br>(*TMG, clinical sub-group*)	Professor of Clinical Memory Research, Lund University

	oskar.hansson@med.lu.se
Paola Piccini<br>(*TMG, clinical sub-group*)	Professor of Neurology, Imperial College London<br>paola.piccini@imperial.ac.uk
Robert Morris<br>(*TMG, clinical sub-group*)	Consultant Neurosurgeon, Cambridge University Hospitals NHS Foundation Trust<br>robert.morris8@nhs.net
Roger Barker<br>Protocol Author, Clinical Lead & Principal Investigator – Cambridge, UK<br>(*TMG, clinical sub-group*)	Professor of Clinical Neurosciences, University of Cambridge and Honorary Consultant in Neurology, Cambridge University Hospitals NHS Foundation Trust<br>rab46@cam.ac.uk
Ruben Smith<br>(*TMG, clinical sub-group*)	Specialist in Neurology, Region Skåne – Skåne University Hospital and Lund University<br>ruben.smith@med.lu.se
Trinette Van Vliet<br>(*TMG*)	Research Coordinator, Region Skåne – Skåne University Hospital<br>Trinette.VanVliet@skane.se

Data and Safety Monitoring Board

Professor Alexander Storch<br>*DSMB Chairperson*	University Medical Center Rostock
Professor Camille Carroll	University of Plymouth
Professor Emerita Elna-Marie Larsson	Uppsala University
Professor Neil Robertson	Cardiff University
Professor Per Svenningsson	Karolinska Institute Stockholm
Professor Simon Skene	University of Surrey
Professor Stéphane Palfi	Henri Mondor Medical Center, Paris University (UPEC)

Table of Contents

1 Protocol Signature Pages 2
1.1 Sponsor signature 2
1.2 Investigator signatures 3
2 Trial Management Committee(s) and Protocol Contributors 5
Table of Contents 8
3 Abbreviations 11
4 Trial Synopsis 14
5 Trial Flow Chart 24
6 Introduction 25
6.1 Background 25
6.2 Data from non-clinical studies 27
6.3 Clinical data 27
6.4 Risk-benefit evaluation 27
7 Rationale for Trial 27
8 Trial Design 28
8.1 Statement of design 28
8.2 Justification of design 28
8.3 Number of centres 32
8.4 Number of participants 32
8.5 Participants trial duration 32
8.6 Trial objectives 33
8.7 Trial outcome measures 33
9 Selection and Withdrawal of Participants 34
9.1 Inclusion criteria 34
9.2 Exclusion criteria 34
9.3 Participant withdrawal criteria 35
10 Trial Treatments and Transplantation 35
10.1 Treatment summary 35
10.2 Non-investigational medicinal products (nIMPs) 41
10.3 Concomitant therapy 43
10.4 Accountability and dispensing 44
11 Administration Device 45
11.1 Device details 45
11.2 Legal status 45
11.3 Device manufacturer 45
11.4 Device supply 45
11.5 Storage 45
11.6 Device accountability 45
12 Procedures and Assessments 45
12.1 Location for assessments 45
12.2 Potential effect of coronavirus 2019 (COVID-19) 46
12.3 Participant identification 46
12.4 Consent 46
12.5 Screening evaluation 47
12.6 TMG clinical sub-group review 48
12.7 Baseline assessments (day -60 to day -1) 48
12.8 UK participants travelling to Lund for surgery 49
12.9 Day before surgery (day -1) 49
12.10 Day of surgery (day 0) 49
12.11 Post-surgery assessments 49
12.12 Schedule of assessments 57

12.13 Summary of assessments 61
12.14 Long-term follow-up assessments 68
12.15 End of trial participation 68
12.16 Trial restrictions 68
13 Assessment of Safety 69
13.1 Definitions 69
13.2 Expected adverse reactions/serious adverse reactions (AR/SARs) 70
13.3 Evaluation of adverse events 71
13.4 Adverse events of special interest (AESIs) 72
13.5 Reporting of AESIs 72
13.6 Reporting of SAEs 72
13.7 Reporting of SUSARs 73
13.8 Pregnancy reporting 74
14 Toxicity – Emergency Procedures 75
15 Evaluation of Results 75
15.1 Primary response criteria 75
15.2 Secondary response criteria 75
15.3 Evaluation of ON measurements 76
16 Storage and Analysis of Samples 76
16.1 Safety blood samples 76
16.2 Optional research blood samples 76
16.3 Optional CSF samples 76
17 Statistics 77
17.1 Statistical methods 77
17.2 Interim analyses 77
17.3 Number of participants to be enrolled 77
17.4 Criteria for the premature termination of the trial 78
17.5 Criteria for temporary halt of transplantations 78
17.6 Procedure to account for missing or spurious data 78
17.7 Definition of the end of the trial 78
18 Data Handling and Record Keeping 78
18.1 Case report form (CRF) 78
18.2 Source data 79
18.3 Data protection & participant confidentiality 79
18.4 Archiving of trial documentation 80
19 Trial Committees 81
19.1 Trial Management Group (TMG) 81
19.2 Data and Safety Monitoring Board (DSMB) 82
20 Ethical & Regulatory Considerations 83
20.1 Ethical committee review 83
20.2 Regulatory compliance 83
20.3 Protocol amendments 83
20.4 Peer review 83
20.5 Declaration of Helsinki and GCP 83
20.6 GCP training 84
21 Sponsorship, Financial and Insurance 84
21.1 Sponsorship 84
21.2 Funding 84
21.3 Insurance 84
22 Monitoring, Audit & Inspection 84
23 Protocol Compliance and Breaches of GCP 84
24 Publications Policy 85
25 Protocol Amendments 86
26 References 92

27 Appendices 96
27.1 Appendix I: Risk-benefit evaluation 96
27.2 Appendix II: Safety assessment flow chart 103
27.3 Appendix III: Planned staggered dosing schedule 104
27.4 Appendix IV: Diagram of Rehncrona-Legradi device 105

3 Abbreviations

ADL	activities of daily living
AE	adverse event
AESI	adverse event of special interest
AIMS	Abnormal Involuntary Movement Scale
ALP	alkaline phosphatase
ALT	alanine transaminase
APTT	activated partial prothromboplastin time
AR	adverse reaction
ARSAC	Administration of Radioactive Substances Advisory Committee (UK)
ATIMP	advanced therapy investigational medicinal product
CAB	Clifford Allbutt Building
CCGTT	Centre for Cell, Gene & Tissue Therapeutics
CE	Conformité Européene
CMV	cytomegalovirus
CNS	central nervous system
CoA	certificate of analysis
COMT	catechol-o-methyl transferase
CRF	case report form
CRP	c-reactive protein
CSF	cerebrospinal fluid
CT	computerised tomography
CTA	clinical trial authorisation
CUH	Cambridge University Hospitals NHS Foundation Trust
DA	dopamine
DAT	dopamine transporter
DBS	deep brain stimulation
DSMB	Data and Safety Monitoring Board
DSUR	Development Safety Update Report
EBV	Epstein-Barr virus
ECG	electrocardiogram
eCRF	electronic case report form
EPM	Swedish Ethical Review Authority (Etikprövningsmyndigheten)
EU	European Union
FBC	full blood count
F-DOPA	[^{18}F]-fluorodopa
FSH	follicle stimulating hormone
GCP	Good Clinical Practice
GDPR	General Data Protection Regulation
GID	graft-induced dyskinesia
GLP	Good Laboratory Practice
GMP	Good Manufacturing Practice
HBV	hepatitis B
HCV	hepatitis C
HDL	high density lipoprotein
hESC	human embryonic stem cell
HIV	human immunodeficiency virus
HLA	human leukocyte antigen
HRA	Health Research Authority (UK)
HSV	herpes simplex virus

HTLV	human T-lymphotropic virus
HVLT-R	Hopkins Verbal Learning Test-Revised
IB	investigator's brochure
ICF	informed consent form
ID	identification number
IgG	immunoglobulin G
IgM	immunoglobulin M
IMP	investigational medicinal product
iPS	induced pluripotent stem cells
ISF	investigator site file
IUD	intrauterine device
IVO	Health and Social Care Inspectorate (Sweden)
JC	John Cunningham
LCIG	levodopa carbidopa intestinal gel
LDL	low density lipoprotein
L-dopa	levodopa
LED	levodopa equivalent dose
LFT	liver function test
LID	levodopa-induced dyskinesia
LU	Lund University
MADRS	Montgomery–Åsberg Depression Rating Scale
MAO-B	monoamine oxidase B
MDS-UPDRS	Movement Disorder Society Unified Parkinson’s Disease Rating Scale
MedDRA	Medical Dictionary for Regulatory Activities
MHC	major histocompatibility complex
MHRA	Medicines and Healthcare products Regulatory Agency (UK)
MoCA	Montreal Cognitive Assessment
MPA	Swedish Medical Products Agency (Läkemedelsverket) (Sweden)
MRI	magnetic resonance imaging
MRSA	methicillin-resistant Staphylococcus aureus
nIMP	non-investigational medicinal product
NMSS	Non-Motor Symptom Scale
OD	once daily
PD	Parkinson’s disease
PDQ-39	Parkinson’s Disease Questionnaire 39
PE2i	[^{18}F]FE-PE2i
PET	positron emission tomography
PI	Principal Investigator
PIC	patient identification centre
PIS	participant information sheet
PKG®	Parkinson KinetiGraph®
PSF	pharmacy site file
PT	prothrombin time
QOL	quality of life
REC	Research Ethics Committee (UK)
RFH	Royal Free Hospital
RIS	Radiology Information System
R-L	Rehncrona-Legradi
RSI	reference safety information
RS-SUS	Region Skåne – Skåne University Hospital
SAE	serious adverse event

SAP	statistical analysis plan
SAR	serious adverse reaction
SNc	substantia nigra pars compacta
SOP	standard operating procedure
SUSAR	suspected unexpected serious adverse reaction
TMF	trial master file
TMG	Trial Management Group
TOF	time of flight
TPMT	thiopurine methyltransferase
U&E	urea and electrolytes
UCAM	University of Cambridge
UCLH	University College London Hospitals NHS Foundation Trust
VGB	John van Geest Centre for Brain Repair
VM	ventral mesencephalic
VZV	varicella-zoster virus
WAIS	Wechsler Adult Intelligence Scale
WBIC	Wolfson Brain Imaging Centre
YOPD	young onset Parkinson’s disease

4 Trial Synopsis

Title of clinical trial	STEM-PD trial: A multicentre, single arm, first in human, dose-escalation trial, investigating the safety and tolerability of intraputamenal transplantation of human embryonic stem cell derived dopaminergic cells for Parkinson's disease (STEM-PD product)
Sponsor	Region Skåne – Skåne University Hospital
EudraCT number	2021-001366-38
Medical condition or disease under investigation	Parkinson's disease
Purpose of clinical trial	To assess the safety, tolerability and feasibility of grafting human embryonic stem cell derived dopaminergic cells into the putamen of patients with moderate PD.
Primary objective	To assess the safety, tolerability and feasibility of intraputamenal transplantation of the STEM-PD product in patients with moderate PD.
Secondary objectives	• To evaluate the course and efficacy on the clinical features following intraputamenal transplantation of the STEM-PD product in patients with moderate PD.<br>• To assess the survival of dopamine cells following transplantation of the STEM-PD product in patients with moderate PD using PET imaging.<br>• To determine the safety and clinical efficacy between doses (if dose escalation is undertaken) of the STEM-PD product including assessment of whether there is a dose response effect.
Trial design	Multicentre, single arm, dose escalation, first in human, ATIMP trial.
Primary trial outcome measures	• The number and nature of AEs and SAEs in the first 12 months following transplantation.<br>• Absence of space occupying masses on cranial MRI in the first 12 months following transplantation.
Secondary trial outcome measures	• Changes in clinical effects at 36 months following transplantation compared to baseline (including emergence of new neurological features, including GIDs, global cognitive changes and changes in non-motor/QOL assessments).<br>• Changes in motor features in the OFF state.<br>• Change in anti-Parkinson medication as measured by LED.<br>• Changes in F-DOPA uptake and DAT binding at 36 months on PET imaging with F-DOPA and PE2i compared to PET imaging performed pre-transplant.<br>• The number and nature of SAEs and AEs in the 12 to 36 months period following transplantation.
Exploratory trial outcome measures	• Changes in PET imaging at 6, 12 and 24 months post-transplant compared to PET imaging performed pre-transplant.<br>• Change in clinical features compared to baseline as determined by measurement using wearable movement monitoring devices.

	• Levels of donor cell specific anti-HLA class I antibodies post-transplant.<br>• Changes in markers of inflammation in the CSF.<br>• Change in response to a L-dopa challenge test including measuring the duration and profile of the L-dopa effects between baseline and 36 months.
Sample size	8 participants will undergo the transplantation
Summary of eligibility criteria	Inclusion criteria:<br>• Have given written informed consent to participate in the trial<br>• Diagnosed with PD as defined using Queens Square Brain Bank criteria<br>• Moderate disease as defined by having Hoehn and Yahr stage 2-3 in OFF state<br>• Disease duration > 10 years<br>• Male or female, aged between 50 and 75 years (inclusive)<br>• Have a significant response to dopamine therapies as judged by the PI or other delegated clinician<br>• Have symptoms that are not appropriately controlled by existing oral anti-PD medications, as judged by the PI or other delegated clinician<br>• Ability to travel to Lund for surgery<br>• Followed up for at least 12 months prior to inclusion in this trial in the TransEUro observational study<br>• Be fluent in English/Swedish to enable completion of questionnaires as assessed by the PI or other delegated clinician at Cambridge/Lund, respectively<br>• Be approved by the TMG clinical sub-group for trial participation<br><br>Exclusion criteria<br>• Tremor dominant disease, as assessed by the PI or other delegated clinician<br>• Significant drug induced dyskinesias as defined by a score of > 2 in the Abnormal Involuntary Movement Scale (AIMS) dyskinesias rating scale, in any body part in the ON state<br>• Ongoing major medical or psychiatric disorders, including depression (MADRS > 20) and psychosis, that make participation unsuitable, as judged by the PI or other delegated clinician<br>• Any contraindication to neurosurgery<br>• Unable to be imaged using MRI<br>• Extensive ventral striatal loss or normal findings on F-DOPA PET at screening<br>• Significant cognitive impairment indicative of an incipient dementia/established dementia or values consistent with MoCA score of ≤ 24<br>• Unable to perform normal copying of interlocking pentagons and/or a semantic fluency score for naming animals of less than 20 over 90 seconds

	• Other concomitant treatment with neuroleptics (including atypical neuroleptics) and/or cholinesterase inhibitors<br>• Previous neurosurgery to the brain, or cell or organ transplantation, or recipient of repeated blood transfusions<br>• Any contraindication to immunosuppressive therapy, prophylactic antibiotics, and/or osteoporosis prophylaxis (refer to STEM-PD Trial Immunosuppressant Manual)<br>• High levels of pre-formed specific anti-HLA antibodies to the cell product<br>• Severely reduced TPMT activity (less than half of the lower normal TPMT activity level)<br>• History of documented severe/significant allergy requiring treatment<br>• Female who is pregnant or breastfeeding<br>• Received an investigational drug (including investigational vaccines) or used an invasive investigational medical device within 4 weeks of the screening visit, or is currently enrolled in an interventional investigational trial<br>• Female of childbearing potential or male unwilling to follow contraception requirements (see protocol section 12.15)<br>• Any other condition which, in the opinion of the investigator, makes the patient inappropriate for entry into the trial
Investigational medicinal product and dosage	STEM-PD product<br><br>Doses are expressed as the expected number of surviving dopamine neurons per putamen, which from non-clinical efficacy testing is estimated to be 2,825 surviving dopamine neurons obtained per 100,000 transplanted STEM-PD cells after graft maturation.<br><br>Two doses are proposed for the trial:<br>• Dose 1: aim for 100,000 surviving dopamine neurons per putamen = 3.54×10^6 STEM-PD cells grafted per putamen<br>• Dose 2: aim for 200,000 surviving dopamine neurons per putamen = 7.08×10^6 STEM-PD cells grafted per putamen
Active comparator product(s)	None
Route(s) of administration	Intraputamenal
Maximum duration of treatment of a participant	Single dose
nIMPs	Participants will receive the following nIMPs:<br>• Antibiotics<br>○ Prophylactic antibiotics at the time of surgery (as per local practices)<br>○ Trimethoprim-sulfamethoxazole<br>• Immunosuppressants

	○ IV methyl prednisolone sodium succinate<br>○ Tacrolimus (if tolerated, otherwise ciclosporin)<br>○ Azathioprine<br>○ Prednisolone<br>○ Basiliximab<br>• Osteoporosis prophylaxis<br>○ Alendronic acid (or locally approved equivalent)<br>○ Calcium /vitamin D<br>• PET ligands<br>○ [^{18}F]-fluorodopa (F-DOPA)<br>○ [^{18}F]FE-PE2i (PE2i)<br>• L-dopa challenge dose (soluble L-dopa with a dopa decarboxylase inhibitor)
Administration device	The Rehncrona-Legradi (R-L) device: a non-CE marked class III neurosurgical medical device. The R-L device system is comprised of:<br>• A R-L injection device<br>• A guide cannula and blunt stylet<br>• A loading station
Procedures:<br>Screening (day -120 to day -7)	• Informed consent<br>• Assessment of eligibility<br>• Concomitant medication (including PD medication)<br>• Height and weight<br>• Physical examination<br>• Demographics<br>• Medical history<br>• Years of education<br>• Smoking status<br>• AE assessment<br>• Serum pregnancy test (for female participants of childbearing potential)<br>• Blood samples: FBC, U&E, LFTs, coagulation tests, CRP, microbiology/serology, HLA antibody titres, glucose, HbA1c, blood lipids, TPMT<br>• MRSA (for Cambridge participants only)<br>• Imaging: MRI (screening), PET (F-DOPA)/CT<br>• Motor assessments<br>○ ON and OFF state: MDS-UPDRS part III, AIMS<br>○ MDS-UPDRS parts I, II and IV<br>• Cognitive assessments (ON state): MoCA, pentagon copying, semantic (animal naming) fluency<br>• Psychiatric assessments: MADRS
Procedures:<br>Baseline (day -60 to day -1)	• Concomitant medication (including PD medication)<br>• Weight<br>• ECG<br>• Vital signs<br>• Physical examination<br>• AE assessment<br>• Serum or urine pregnancy test (applicable only for women of childbearing potential)<br>• **Optional** research blood sample

	• **Optional** lumbar puncture & accompanying blood samples<br>• Imaging: MRI (surgical planning), CT angiogram, MRI (extended battery), PET (PE2i)/CT<br>• Motor assessments:<br>○ ON and OFF state: MDS-UPDRS part III, 9 hole peg test, timed sit-stand-walk test, RUSH dyskinesia scale, AIMS, 30 second tap test<br>○ MDS-UPDRS part I, II, and IV<br>○ L-dopa challenge test<br>○ Collection of Hauser patient diary card and PKG®<br>• Cognitive assessments:<br>○ ON state: HVLT-R, Stroop test, digit span test, WAIS similarities test, Boston naming task<br>• Psychiatric assessments: MADRS<br>• Non-motor/QOL assessments: PDQ-39, EQ-5D-5L, NMSS<br>• Provision of participant contact/alert card
Procedures:<br>Day before surgery (day -1)	• Concomitant medication (including PD medication)<br>• Weight<br>• Immunosuppressant commencement<br>• Serum or urine pregnancy test (for female participants of childbearing potential)<br>• Blood samples: FBC, U&E, LFTs, coagulation tests, CRP, cross match testing, glucose, HbA1c, blood lipids
Procedures:<br>Surgery (day 0)	• Concomitant medication (including PD medication)<br>• Vital signs<br>• AE assessment<br>• Imaging: MRI (with frame)<br>• Surgery (transplantation of STEM-PD product)
Procedures:<br>Days 1 and 2 post-transplantation	• Concomitant medication (including PD medication)<br>• ECG (*day 2 only*)<br>• Vital signs<br>• AE assessment<br>• Blood samples:<br>○ FBC, U&E, tacrolimus/ciclosporin level, LFTs, CRP, glucose<br>○ *day 1 only*: plus blood lipids<br>• Imaging (*day 2 only*): MRI (safety, ± 24 hours)
Procedures:<br>Days 7, 14, 21, 28, 42 post-transplantation (± 2 days)	• Concomitant medication (including PD medication)<br>• Weight<br>• Vital signs<br>• Physical examination<br>• AE assessment<br>• *Day 28 only*: Serum or urine pregnancy test (for female participants of childbearing potential)<br>• Review nIMP compliance<br>• Blood samples:<br>○ *All visits*: FBC, U&E, tacrolimus/ciclosporin level, LFTs, CRP, glucose

	○ *Days 7, 14 and 28 only*: plus blood lipids<br>• Imaging (*day 28 only*): MRI (safety)<br>• Psychiatric assessments (*day 28 only*): MADRS
Procedures:<br>Months 2, 3 and 4 post-transplantation (± 1 week)	• Concomitant medication (including PD medication)<br>• Weight<br>• Vital signs<br>• Physical examination<br>• AE assessment<br>• Serum or urine pregnancy test (for female participants of childbearing potential)<br>• Review nIMP compliance<br>• Blood samples:<br>○ *All visits*: FBC, U&E, tacrolimus/ciclosporin level, LFTs, CRP, glucose<br>○ *Month 3 only*: plus HLA antibody titres, HbA1c<br>○ *Months 2 and 3 only*: plus blood lipids<br>• Imaging (*months 2 and 3 only*): MRI (safety)<br>• Motor assessments (*month 3 only*):<br>○ ON state: MDS-UPDRS part III, 9 hole peg test, timed sit-stand-walk test, RUSH dyskinesia scale, AIMS, 30 second tap test<br>○ MDS-UPDRS parts I, II and IV<br>○ Collection of Hauser patient diary card and PKG® (Lund participants only)<br>• Psychiatric assessments (*month 3 only*): MADRS
Procedures:<br>Month 5 post-transplantation (± 1 week)	• Concomitant medication (including PD medication)<br>• Weight<br>• Vital signs<br>• Physical examination<br>• AE assessment<br>• Serum or urine pregnancy test (for female participants of childbearing potential)<br>• Review nIMP compliance<br>• Blood samples: FBC, U&E, tacrolimus/ciclosporin level, LFTs, CRP, glucose
Procedures:<br>Month 6 post-transplantation (± 1 week)<br><br>(*PET & MRI imaging ± 1 month)	• Concomitant medication (including PD medication)<br>• Weight<br>• Vital signs<br>• Physical examination<br>• AE assessment<br>• Serum or urine pregnancy test (for female participants of childbearing potential)<br>• Review nIMP compliance<br>• Blood samples: FBC, U&E, tacrolimus/ciclosporin level, LFTs, CRP, HLA antibody titres, glucose, HbA1c, blood lipids<br>• Imaging*:<br>○ ***Optional for Cambridge participants****:* PET (F-DOPA)/CT<br>○ *All participants*: MRI (safety)<br>○ ***Optional for Cambridge participants***: MRI (extended battery)<br>• Motor assessments:

	○ ON and OFF state: MDS-UPDRS part III, 9 hole peg test, timed sit-stand-walk test, RUSH dyskinesia scale, AIMS, 30 second tap test<br>○ MDS-UPDRS parts I, II and IV<br>○ L-dopa challenge test<br>○ Collection of Hauser patient diary card and PKG® (Lund participants only)<br>• Cognitive assessments (ON state): MoCA<br>• Psychiatric assessments: MADRS<br>• **Optional** lumbar puncture & accompanying blood samples[1]<br>• **Optional** research blood sample
Procedures<br>Months 7 and 8 (± 1 week)	• For female participants of childbearing potential only: urine pregnancy test, to be completed remotely
Procedures:<br>Month 9 post-transplantation (± 1 week)<br><br>(*MRI imaging ± 1 month)	• Concomitant medication (including PD medication)<br>• Weight<br>• Vital signs<br>• Physical examination<br>• AE assessment<br>• Serum or urine pregnancy test (for female participants of childbearing potential)<br>• Review nIMP compliance<br>• Blood samples: FBC, U&E, tacrolimus/ciclosporin level, LFTs, CRP, HLA antibody titres, glucose, HbA1c, blood lipids<br>• Imaging: MRI (safety)*<br>• Motor assessments:<br>○ ON state: MDS-UPDRS part III, 9 hole peg test, timed sit-stand-walk test, RUSH dyskinesia scale, AIMS, 30 second tap test<br>○ MDS-UPDRS parts I, II and IV<br>○ Collection of Hauser patient diary card and PKG® (Lund participants only)
Procedures:<br>Months 10 and 11 (± 1 week)	• For female participants of childbearing potential only: urine pregnancy test, to be completed remotely
Procedures:<br>Month 12 post-transplantation (± 1 week)<br><br>(*PET & MRI imaging ± 1 month)	• Concomitant medication (including PD medication)<br>• Weight<br>• Vital signs<br>• Physical examination<br>• AE assessment<br>• Serum or urine pregnancy test (for female participants of childbearing potential)<br>• Review nIMP compliance<br>• Blood samples: FBC, U&E, tacrolimus/ciclosporin level, LFTs, CRP, HLA antibody titres, glucose, HbA1c, blood lipids<br>• Imaging*:<br>○ *All participants:* PET (F-DOPA)/CT<br>○ *All participants*: MRI (safety)<br>○ ***Optional for Cambridge participants****:* PET (PE2i)/CT, MRI (extended battery)

	• Motor assessments:<br>○ ON and OFF state: MDS-UPDRS part III, 9 hole peg test, timed sit-stand-walk test, RUSH dyskinesia scale, AIMS, 30 second tap test<br>○ MDS-UPDRS parts I, II and IV<br>○ L-dopa challenge test<br>○ Collection of Hauser patient diary card and PKG® (Lund participants only)<br>• Cognitive assessments (ON state): MoCA<br>• Psychiatric assessments: MADRS<br>• Non-motor/QOL assessments: PDQ-39, EQ-5D-5L, PD NMSS
Procedures:<br>Months 13, 14, 15, 16 and 17 (± 1 week)	• For female participants of childbearing potential only: urine pregnancy test, to be completed remotely
Procedures 15+ months post transplantation (up to 2 weeks following completion of immunosuppressants)[2]	• Concomitant medication (including PD medication)<br>• Vital signs<br>• Weight<br>• AE assessment<br>• Review nIMP compliance
Procedures:<br>Month 18 post-transplantation (± 2 weeks)	• Concomitant medication (including PD medication)<br>• Vital signs<br>• Physical examination<br>• AE assessment<br>• Serum or urine pregnancy test (for female participants of childbearing potential)<br>• Review nIMP compliance<br>• Blood sample: HLA antibody titres, glucose, HbA1c, blood lipids<br>• **Optional** research blood sample<br>• **Optional** lumbar puncture & accompanying blood sample[1]<br>• Motor assessments:<br>○ ON state: MDS-UPDRS part III, 9 hole peg test, timed sit-stand-walk test, RUSH dyskinesia scale, AIMS, 30 second tap test<br>○ MDS-UPDRS parts I, II and IV<br>○ Collection of Hauser patient diary card and PKG® (Lund participants only)
Procedures:<br>Month 24 post-transplantation (± 2 weeks)<br><br>(*PET & MRI imaging ± 1 month)	• Concomitant medication (including PD medication)<br>• Vital signs<br>• Physical examination<br>• AE assessment<br>• Blood sample: HLA antibody titres<br>• Imaging*:<br>○ ***Optional for Cambridge participants****:* PET (F-DOPA)/CT<br>○ *All participants*: MRI (safety)<br>○ ***Optional for Cambridge participants***: PET (PE2i)/CT, MRI (extended battery)<br>• Motor assessments<br>○ ON and OFF state: MDS-UPDRS part III, 9 hole peg test, timed sit-stand-walk test,

	RUSH dyskinesia scale, AIMS, 30 second tap test<br>o MDS-UPDRS parts I, II and IV<br>o L-dopa challenge test<br>o Collection of Hauser patient diary card and PKG® (Lund participants only)<br>• Cognitive assessments (ON state): MoCA<br>• Psychiatric assessments: MADRS<br>• Non-motor/QOL assessments: PDQ-39, EQ-5D-5L, PD NMSS
Procedures:<br>Month 30 post-transplantation (± 2 weeks)	• Concomitant medication (including PD medication)<br>• Vital signs<br>• Physical examination<br>• AE assessment<br>• Blood sample: HLA antibody titres<br>• Motor assessments:<br>o ON state: MDS-UPDRS part III, 9 hole peg test, timed sit-stand-walk test, RUSH dyskinesia scale, AIMS, 30 second tap test<br>o MDS-UPDRS parts I, II and IV<br>o Collection of Hauser patient diary card and PKG® (Lund participants only)
Procedures:<br>Month 36 post-transplantation (± 2 weeks)<br><br>(*PET & MRI imaging ± 1 month)	• Concomitant medication (including PD medication)<br>• ECG<br>• Vital signs<br>• Physical examination<br>• AE assessment<br>• Blood sample: HLA antibody titres<br>• **Optional** research blood sample<br>• Imaging* *all participants*:<br>o MRI (extended battery) with combined safety MRI<br>o PET (F-DOPA)/CT<br>o PET (PE2i/CT)<br>• Motor assessments:<br>o ON and OFF state: MDS-UPDRS part III, 9 hole peg test, timed sit-stand-walk test, RUSH dyskinesia scale, AIMS, 30 second tap test<br>o MDS-UPDRS parts I, II and IV<br>o L-dopa challenge test<br>o Collection of Hauser patient diary card and PKG® (Lund participants only)<br>• Cognitive tasks:<br>o ON state: MoCA, HVLT-R, Stroop test, digit span test, WAIS similarities test, Boston naming task<br>• Psychiatric assessments: MADRS<br>• Non-motor/QOL assessments: PDQ-39, EQ-5D-5L, PD NMSS<br>• Enrol to long-term follow-up study
Procedures for safety monitoring during trial	• Reviews by Trial Management Group (TMG) and Data and Safety Monitoring Board (DSMB)<br>• Safety monitoring of participants at each visit

	• Premature termination would be triggered by concerns raised by the investigators and/or the DSMB regarding safety & ethical implications of continuing the trial
Criteria for withdrawal of participants	• Participant decision<br>• Investigator decision prior to surgery (e.g. due to AE which precludes transplantation)

[1] Where possible, blood analysis will be performed on samples already collected
[2] If the completion of immunosuppressants is within 2 weeks of the scheduled 18 month post-surgery visit, then this visit and the assessments listed will not take place.

5 Trial Flow Chart

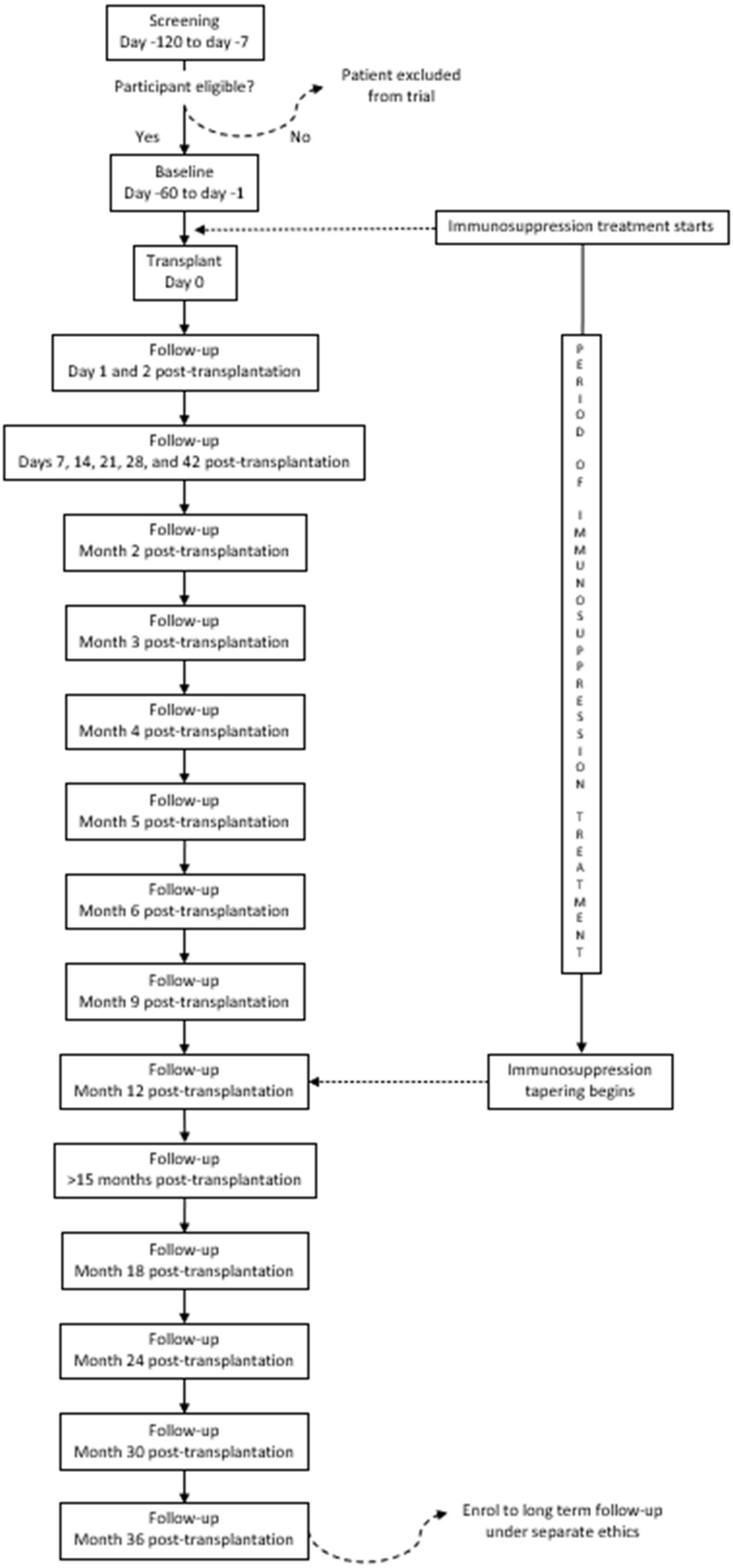

6 Introduction

6.1 Background

6.1.1 Disease background

Parkinson's disease (PD) is a common neurodegenerative disease affecting approximately 1% of people over 65 years of age, and as such is likely to become more common as the population ages and life expectancy increases (Barker & Foltynie, 2004). Approximately 10% of all PD patients are diagnosed at an age earlier than 50 years old and, for these patients, a disease course for more than 20 years is likely. PD is characterised clinically by the development of bradykinesia, rigidity and a resting tremor, which has been attributed in part to the progressive degeneration of the dopaminergic input from the substantia nigra to the striatum.

The striatum has widespread connectivity with several other nuclei and brain regions, contributing to the many symptoms of dopamine deficiency in PD. The cause for this progressive loss of nigrostriatal dopaminergic neurons is not entirely understood, but it is associated with the presence of alpha-synuclein positive Lewy bodies, in some of the remaining substantia nigra pars compacta neurons (SNc) at post-mortem. However, Lewy bodies are also found in many other parts of the brain (including a range of subcortical and brainstem nuclei as well as the cortex) and this may underlie some of the non-motor features of PD such as cognitive, sleep, and affective problems, as well as autonomic dysfunction, which are found in the majority of cases of PD. Indeed, it is increasingly being understood that PD is a disorder which has widespread pathology from its onset and that therefore the nigral pathology is only part of a much more widespread pathological process (Braak et al., 2006; Braak et al., 2004). However, whilst this is true, the core loss of the dopaminergic nigrostriatal pathway is not disputed, nor the effects of treatment targeting this pathway in the clinic.

Thus, despite this non-nigral, non-dopaminergic pathology, the principal feature of the disease remains the progressive loss of dopamine (DA) in the striatum, which can be treated successfully with a range of symptomatic dopaminergic drug therapies. Thus, both levodopa (L-dopa) and DA agonists significantly help many of the features of PD, particularly in the early stages of the disease (Lang & Lozano, 1998a, 1998b). However, with time, the symptoms progress and this coupled to the long-term use of dopaminergic drug therapies produces a range of problems including the development of drug induced motor complications such as 'ON-OFF' fluctuations and levodopa-induced dyskinesias (LIDs). At this stage of the disease, drug therapies become increasingly disappointing in terms of a reliable clinical benefit (Lewis et al., 2003). Therefore, other therapeutic approaches are used, including more invasive ways of delivering continuous dopaminergic therapy, such as apomorphine pumps and DuoDopa® (levodopa carbidopa intestinal gel (LCIG) delivery of L-dopa into the small bowel via a small indwelling catheter and a pump), as well as neurosurgical interventions such as deep brain stimulation (DBS), especially of the subthalamic nucleus (Witt et al., 2008).

These latter therapies can be very effective, but only treat the symptoms without any attempt to repair the underlying diseased network. Thus, these treatments also start to fail, in part because of the continued loss of nigral dopaminergic neurons (Voon et al., 2008; Wider et al., 2008). Therefore, whilst an improved understanding of disease pathogenesis may enable us to better treat all aspects of PD, more restorative approaches to repairing the dopaminergic nigrostriatal tract, including cell replacement, neurotrophic support and pharmacological and gene therapies, may also prove very useful (Evans & Barker, 2008; Wijeyekoon & Barker, 2009).

6.1.2 Transplantation as a treatment option

One of the most effective reparative therapies in patients to date has been with allotransplants of DA neuroblasts obtained from foetal ventral mesencephalic (VM) tissue. However, this cell transplantation approach has given inconsistent results. Some grafted patients have responded extremely well and have come off all their anti-PD medication for years, whilst others have shown no or only modest clinical improvements (Brundin et al., 2000; Defer et al., 1996; Freed et al., 1992; Hagell et al., 1999; Hauser et al., 1999; Mendez et al., 2002). Moreover, a subset of patients also developed severe, off-state graft-induced dyskinesias (GIDs), which in a few cases have required additional neurosurgical intervention (Freed et al., 2001; Hagell et al., 2002; Olanow et al., 2003). The reasons for this have been debated and thought to relate to:

- (i) patient selection (less advanced patients tend to have a larger benefit and less risk of developing GIDs and they also have less ventral striatal DA loss, which has been shown to be a predictor of clinical efficacy of the transplant);
- (ii) the distribution of DA cells across the grafted striatum;
- (iii) the presence of contaminating serotoninergic neurons in the transplants;
- (iv) the variability in the grafted tissue implanted given each patient has their own unique transplant made from tissue collected from termination of pregnancies.

As a result, a trial was undertaken using foetal VM tissue across Europe (TransEUro, NCT01898390) (Barker & consortium, 2019), which has now reached its primary endpoint (long-term follow-up of the participants is still ongoing). However, as became evident early on in this study, tissue availability made it impossible for the trial to proceed at the speed and numbers of patients needed to truly address these issues. Furthermore, some of the above issues could not be answered with foetal tissue allografts, especially the delivery of a standardised tissue implant, which would only be possible with stem cell derived DA cells.

6.1.3 Human pluripotent stem cell transplantation

Given these issues and the ethical and logistical problems inherent in using human foetal tissue, preclinical efforts have sought to produce DA neurons from human stem cell sources – most notably from human embryonic stem cells (hESCs). This work has led to the derivation of protocols in 2011 (Kriks et al., 2011) and 2012 (Kirkeby et al., 2012), which have been shown to reproducibly and robustly give rise to authentic midbrain DA cells of the type lost in PD. Subsequently these cells have been shown to:

- (i) survive long term in immunocompromised donor rats;
- (ii) differentiate into dopaminergic cells upon grafting, produce long-range target specific projections, and to make pre- and post-synaptic contact with neurons of the host animal;
- (iii) reverse motor deficits in animal models of PD with equivalent potency to human foetal-derived DA cells;
- (iv) not form teratomas or any other tumours upon transplantation (Adler et al., 2019; Aldrin-Kirk et al., 2016; Cardoso et al., 2018; Grealish et al., 2015; Heuer et al., 2016; Hoban et al., 2020; Kirkeby et al., 2012; Kirkeby et al., 2017; Lehnen et al., 2017; Nolbrant et al., 2017; Tiklova et al., 2020).

Recent work by the collaborators involved with the STEM-PD trial has led to the development of protocols, which are Good Manufacturing Practice (GMP) compatible, and show good survival and functional efficacy upon transplantation in rodent models of

PD (Adler et al., 2019; Cardoso et al., 2018; Kirkeby et al., 2017; Nolbrant et al., 2017). Manufacturing has been shown to be efficient, which would make such a PD therapy clinically competitive if trials showed safety and efficacy.

While this work has been pursued by our groups in Europe (namely Parmar/Kirkeby in Lund and Barker in Cambridge), other groups from around the world have taken similar approaches with other types of human pluripotent stem cells (Barker et al., 2017). These efforts involved a Japanese study led by Jun Takahashi which entered a clinical trial starting in 2018 using a human induced pluripotent stem cell source (Normile, 2018). A phase I/II clinical trial with a similar hESC-derived DA cell product developed by the team of Lorenz Studer at the Sloan Kettering Institute in New York has also recently been granted clinical trial authorisation by the Food and Drug Administration, and the first patient was transplanted in June 2021 (Communications, 2021).

6.1.4 STEM-PD product

The STEM-PD product is a cryopreserved cell product, consisting of ventral midbrain dopaminergic progenitor cells derived from the clinical-grade hESC line RC17, which was chosen as it reliably gives rise to high-purity VM progenitor cells. In addition, the RC lines from Roslin Cells Ltd. have been derived and cultured under full GMP conditions and RC17 has been tested according to regulatory standards.

A highly efficient protocol has been established for producing transplantable VM DA progenitor cells involving 16 days of directed differentiation of pluripotent hESCs (Nolbrant et al., 2017), and this is the protocol used to manufacture the STEM-PD product.

6.2 Data from non-clinical studies

Data from non-clinical studies are fully outlined in the Investigator’s Brochure (IB).

6.3 Clinical data

As this is a first in human trial, there is currently no clinical data available using the STEM-PD product. Earlier experiences from our group with foetal VM transplants are described in the IB.

6.4 Risk-benefit evaluation

See Appendix I for a detailed description of the risks for the participants; what measures have been taken to reduce those risks, and the possible benefits for the participants. Our conclusion is that the trial has a positive risk-benefit balance.

7 Rationale for Trial

There are currently no reparative therapies for PD. Previous studies using human foetal DA cells have shown that transplants of these cells can survive long-term when transplanted into the striatum of patients with advanced PD. Furthermore, these grafts can restore DA levels at this site to normal and dramatically improve the patients’ clinical state back to near normality for many years (e.g. (Kefalopoulou et al., 2014)).

Therefore, in this trial, we are assessing whether bilateral transplants of midbrain dopaminergic neuronal precursor cells derived from hESCs will survive grafting and differentiate into DA neurons in the striatum of patients with moderate PD. We will further assess whether this can be shown to be safe, feasible and well-tolerated, with some preliminary signs of efficacy. We plan to graft participants with moderate PD who have already shown clear benefits from oral dopaminergic therapies.

The transplants of the STEM-PD product are expected to mature over a period of at least one year with an expected maximal benefit seen 2-3 years post grafting, or possibly even longer. Participants will be followed up until 36 months post-transplantation under this protocol. Long-term follow up (until withdrawal or death) will be carried out under a separate protocol and ethics application.

There are currently no Conformité Européene (CE) marked devices available that have been designed specifically for delivering cells to the brains of patients with PD. The STEM-PD product will be administered using a non-CE marked class III neurosurgical medical device, the Rehncrona-Legradi (R-L) device. This device has been successfully used in Lund for delivering foetal tissue to the brains of patients with PD over the last 30 years and most recently in the TransEUro study where transplants were carried out between 2015-2018. The R-L device is an in-house manufactured device (at Region Skåne – Skåne University Hospital [RS-SUS]) that will not be subject of investigation in this trial. No data (aside from safety data as required for in-house manufactured medical devices) will be specifically collected on the R-L device.

8 Trial Design

8.1 Statement of design

This is a multicentre, single arm, dose escalation, first in human advanced therapy investigational medicinal product (ATIMP) trial.

8.2 Justification of design

8.2.1 <u>Patient group</u>

8.2.1.1 *Stage of disease*

This type of cell replacement therapy is envisioned to be a one-time treatment for patients with mild to moderate PD aiming at long-lasting restorative anti-parkinsonism effects with a magnitude similar to, or exceeding that, of some patients grafted with foetal cell-derived dopaminergic cells.

In this trial we will include patients with moderate PD (Hoehn and Yahr stage 2-3 in the OFF state), with fluctuating disease but who have not yet reached the more severe advanced phase of the condition with uncontrollable motor fluctuations. There are good scientific reasons that the more advanced stages of disease are less likely to benefit from cell replacement treatment and these include:

- Patients that have developed severe fluctuations with abnormal movements, induced by the disease and the medication – so-called LIDs – will not be included. The reason for this is that the presence of LIDs and a more advanced disease stage correlated with the development of so-called GIDs in the earlier studies with foetal dopaminergic cell transplantations.
- Patients in the more advanced stage of PD (Hoehn and Yahr stage 4), i.e. severely disabled patients, will also not be included because the transplant is expected to take some time (> 1 year) to have any benefit. Such patients are therefore more likely to require an invasive intervention, such as pump strategies or DBS, before the transplant can be seen to be effective.
- Patients in the more severe stages may also have more pronounced autonomic dysfunctions, rendering them more prone to immediate complications in relation to surgery and long anaesthetic procedures.
- Patients with the most advanced disease (Hoehn and Yahr stage 5), i.e. patients who are at least part time bedridden due to lack of medication effects, are

unsuitable because of the very high risk that they will develop severe complications of the surgery due to immobility and the ensuing immunosuppressive treatment.

Although the primary aim of this trial is safety and feasibility, considering the burden and risks of the trial, we strongly advocate for medical and ethical reasons to include patients that might eventually also benefit from the transplant, which is why we have selected patients with moderate PD.

8.2.1.2 *Age limitations*

Patients above 75 years of age are excluded in accordance with the standard clinical guidelines that apply to neurosurgical procedures for PD, such as DBS, as the rate and risk for complications increases after this age. The delayed potential benefits of the grafts also make it less likely that older patients will benefit from the treatment.

Although cell replacement treatment strategies are likely to benefit patients with young onset PD (YOPD), patients younger than 50 years will not be included in this trial. This is because YOPD patients often respond well to oral medications and progress slowly and thus any intervention of the type being administered in this trial is harder to justify.

In addition, the radiation exposure limitations related to the planned positron emission tomography (PET) scans are age dependent and the risk/benefit is unfavourable for younger individuals, in particular for women. This, therefore, makes YOPD patients a less than ideal initial population to target with this therapeutic at this stage of trialling.

8.2.2 Surgery at a single site

All participants, whether recruited from the Cambridge or Lund site, will undergo surgery at RS-SUS. The STEM-PD product will be administered using a non-CE marked class III neurosurgical medical device, the R-L device. The device is manufactured by RS-SUS, for use in-house only and all participants will therefore undergo surgery at RS-SUS.

8.2.3 Bilateral transplants

Transplantation of the cells will be done bilaterally in one surgical session, because:

a) Staged transplantation with an identical cell product (in spite of immunosuppressive treatment) may increase the risk of immunological rejection of the second transplant, as well as jeopardising the survival of the first graft.
b) A bilateral procedure performed by an experienced neurosurgeon is expected to take up to 10 hours, which is acceptable for the type of patients to be included and comparable to surgery for DBS using electrophysiological recording targeting techniques. A unilateral procedure would take at least 6 hours, and then another 6 hours for the surgery of the second hemisphere at a later time point.
c) A bilateral procedure will mean the patient only requires one prolonged period of general anaesthesia as opposed to two in a relatively short time if sequential grafting is undertaken.
d) The induction of immunosuppressive treatment will be limited to one session and the overall duration of the immunosuppression will be shorter, reducing some of the risks for the participants.

To limit the risk of reduced cell viability during the surgery, the STEM-PD product will be thawed, washed and resuspended in delivery vehicle at the clinical site immediately prior to transplantation (for details on product preparation, refer to STEM-PD SOP1 and

the Pharmacy Manual [STEM-PD SOP9]).

8.2.4 Single arm trial

This is the first time the STEM-PD product will be administered to humans. The main purpose of this trial is to investigate safety, tolerability and feasibility of the transplantation using the STEM-PD product.

To include a control group in this trial with only 8 participants is not considered meaningful, because:

a) The trial is a first in human trial that is not primarily assessing efficacy.
b) Safety issues will be clearly related to the STEM-PD product, and a control group is not needed for the trial endpoints to be recognised.
c) In terms of distinguishing intervention effects from those relating to the underlying PD, a more relevant general cohort of patients already exists in the form of the TransEUro observational cohort of patients with PD (Barker & consortium, 2019). These patients have been followed up for 10 years and it is from this cohort that patients will be selected to be part of the STEM-PD trial. As such, the non-grafted patients followed up in the TransEUro observational cohort can act as an indirect comparator to the transplanted participants. In addition, we are also following up 11 patients who have been grafted within the TransEUro programme with foetal dopaminergic cells that can also serve as historical controls and reference patients.
d) The course of graft development can be measured objectively using magnetic resonance imaging (MRI) and PET with the participant's baseline data as the control time-point.

8.2.5 Dose of STEM-PD product

Refer to protocol section 10.1.1.8 and the IB (section 6.2: Planned dose and method of administration) for details on dose calculation.

Doses are expressed as the expected number of surviving DA neurons per putamen. From non-clinical efficacy and potency studies using the clinical batch of STEM-PD (GMP batch #3), it has been shown that 100,000 transplanted STEM-PD cells give rise to 2,825 surviving DA neurons after graft maturation. As the non-clinical studies have used the same batch of STEM-PD product as will be used in the clinical trial, the transplantation doses are calculated from this number.

8.2.5.1 *Starting dose (dose 1)*

The starting dose of this trial is selected as a dose of cells that is likely to be the minimal therapeutic dose, i.e. 100,000 surviving DA neurons per putamen, obtained by transplanting 3.54 million STEM-PD cells per putamen. We regard that it is unethical to use a lower dose that would be expected not to be clinically effective due to the risks related to the transplantation procedure and the one-year long immunosuppressive therapy (both of which are unrelated to the cell dose).

8.2.5.2 *Dose 2*

To ensure that we are not using a potentially suboptimal cell dose, we also plan to test a higher dose, which is double the dose of dose 1. The dose 2 of 200,000 surviving DA neurons (= 7.08 million transplanted STEM-PD cells per putamen) has been chosen as the dose 2 needs to be substantially higher than dose 1 to be able to observe possible dose differences with only 4 patients per dose. In addition, the higher dose would give an estimated number of surviving DA neurons that is still within the range that has been shown to produce clinical benefits but without side effects in human foetal VM

grafted patients, and well within the range of the number of DA neurons that normally innervate the putamen in healthy humans.

The decision as to how we will move from dose 1 to dose 2 is as follows: once all of the 4 participants in the dose 1 group have reached the 6 months follow-up, data relating to clinical evaluations, MRI scans and their 6 months [^{18}F]-fluorodopa (F-DOPA) PET imaging will be reviewed by the Data and Safety Monitoring Board (DSMB) in conjunction with the clinical sub-group of the Trial Management Group (TMG). The DSMB will make a recommendation on how to proceed, and a decision will be made by the TMG around the progression to dose 2. The DSMB can also recommend to wait for additional data (e.g. up to the 12 month follow-up with imaging) – see section 19.2.1 for further information.

8.2.6 Timing of trial objective evaluation

Although it expected that dopaminergic neurons will form within 4-6 months of being implanted, it is anticipated that it will take considerably longer for these dopaminergic neurons to grow fibres to allow full innervation of the surrounding putamen which is needed for them to have clinical benefits in patients. As such we would expect the maximal benefits from the grafted cells to take several years to become manifest, although we would expect to see dopamine cell survival at earlier time points. For this reason, we have decided to assess some of the secondary trial objectives (e.g. PET imaging) at later time-points, whilst continuing to also perform the assessments earlier to evaluate cell survival ahead of fibre outgrowth and functional benefits.

8.2.7 PET and extended battery MRI imaging

It is essential that all imaging for each individual participant is done at a single centre, given that PET imaging is very sensitive to site and type of camera used to collect the data. All participants will undergo all PET imaging at RS-SUS.

The parameters in the extended battery MRI are also machine-dependent, and so it is essential that this is done at a single centre. These scans will be performed at the same time point as the PET imaging, in order to reduce the burden for participants. Participants recruited from Cambridge will therefore undergo the extended battery MRI at RS-SUS, at the same time points as PET imaging.

Cambridge participants travelling to Lund will be accompanied by a member of the trial team, and all travel, accommodation and living costs will be covered.

8.2.8 Non-IMPs

Full details of the non-investigational medicinal products (nIMPs) to be used are provided in section 10.2.

8.2.8.1 *Immunosuppressant regime*

The proposed immunosuppressant regime matches that which is used as standard in organ transplant practice and has been developed in conjunction with our local transplantation clinical colleagues. Transplantation of allogeneic tissues and organs will lead to recognition of the grafted tissue and organ by the host immune system and a response elicited. Thus, some level of immunosuppression is needed to ensure that the hosts immune system does not reject the grafted cells. This need for use of immunosuppressive treatment to allow for optimal graft survival is best exemplified by the experimental studies comparing allogeneic (same species different individuals) dopaminergic cells produced from induced pluripotent stem cells (iPS) with autologous (same species and same individual) cells with and without immunosuppression (Morizane et al., 2017). The surviving cell numbers are significantly higher with the use

of immunosuppressive treatment when allogeneic cells were grafted. As a result, we are adopting the immunosuppressive regimes used with whole organ transplants that are done routinely in clinic, including the use of agents to protect against possible side effects from such therapies- infections, osteoporosis etc. The major difference with our use of these agents and those done for whole organ grafts, is that we will limit the post-transplant period of immunosuppression to a year as previous foetal dopamine cell trials in PD have shown that after this point grafts appear to survive indefinitely without any further immunosuppression.

8.2.8.2 *Antibiotics*

Prophylactic antibiotics will be used at the time of surgery and for up to 48 hours post-transplant, as per routine practice. Additional antibiotics will be used for the duration of the immunosuppressant treatment.

8.2.8.3 *Osteoporosis prophylaxis*

The proposed osteoporosis prophylaxis is required due to the corticosteroid use and will be mandatory whilst the participant is receiving prednisolone (as outlined in section 10.2.2.5).

8.2.8.4 *PET ligands*

PET imaging is used within the trial to allow for the assessment of the survival of dopaminergic neurons derived from the transplanted STEM-PD product. The proposed PET ligands to be used within the trial are: F-DOPA, which measures DA synthesis/storage capacity of the transplant, and PE2i, which selectively labels mature DA nerve terminals.

8.3 Number of centres

The trial will be conducted at two sites: Cambridge University Hospitals NHS Foundation Trust (CUH) (Cambridge, UK) and RS-SUS (Lund, Sweden).

University College London Hospitals NHS Foundation Trust (UCLH) will act as a patient identification centre (PIC) for the trial.

Surgery will be performed at RS-SUS (Lund, Sweden) for all participants.

All PET/computerised tomography (CT) imaging and extended battery MRI will be performed at RS-SUS.

8.4 Number of participants

Eight participants will be transplanted in this trial.

8.5 Participants trial duration

Recruitment is expected to occur over 18 months (although there will be a break in recruitment to allow for review of the preliminary data from the first 4 participants, to determine which dose will be used moving forward; see above and section 19.2.1). For each participant there will be a screening and baseline visit within 120 days prior to surgery. Participants will be followed up for 36 months post transplantation.

After this time, participants will enter long-term follow up (until withdrawal or death) that will be carried out under a separate ethics application and protocol.

8.6 Trial objectives

8.6.1 Primary objectives

To assess the safety, tolerability and feasibility of intraputamenal transplantation of the STEM-PD product in patients with moderate PD.

8.6.2 Secondary objectives

To evaluate the course and efficacy on clinical features following intraputamenal transplantation of the STEM-PD product in patients with moderate PD.

To assess the survival of DA cells following transplantation of the STEM-PD product in patients with moderate PD using PET imaging.

To determine the safety and clinical efficacy between doses (if dose escalation is undertaken) of the STEM-PD product, including assessment of whether there is a dose response effect.

8.7 Trial outcome measures

8.7.1 Primary outcome measures

- The number and nature of adverse events (AEs) and serious adverse events (SAEs) in the first 12 months following transplantation
- Absence of space occupying masses on cranial MRI in the first 12 months following transplantation

8.7.2 Secondary outcome measures

- Changes in clinical effects at 36 months following transplantation compared to baseline (including emergence of new neurological features, including GIDs, global cognitive changes, and changes in non-motor/quality of life (QOL) assessments)
- Changes in motor features in the OFF state
- Change in anti-Parkinson medication as measured by L-dopa equivalent dose (LED)
- Changes in F-DOPA uptake and dopamine transporter (DAT) binding at 36 months on PET imaging with F-DOPA and PE2i compared to PET imaging performed pre-transplant
- The number and nature of SAEs and AEs in the 12 to 36 months period following transplantation

8.7.3 Exploratory outcome measures

- Changes in PET imaging at 6, 12 and 24 months post-transplant compared to PET imaging performed pre-transplant
- Change in clinical features compared to baseline as determined by measurement using wearable movement monitoring devices
- Levels of donor cell specific anti-human leukocyte antigen (HLA) class I antibodies post-transplant
- Changes in markers of inflammation in the cerebrospinal fluid (CSF)
- Change in response to a L-dopa challenge test including measuring the duration and profile of the L-dopa effects between baseline and 36 months

9 Selection and Withdrawal of Participants

9.1 Inclusion criteria

To be included in the trial the participant must:

- Have given written informed consent to participate in the trial
- Diagnosed with PD as defined using Queens Square Brain Bank criteria
- Moderate disease as defined by having Hoehn and Yahr stage 2-3 in OFF state
- Disease duration > 10 years
- Male or female, aged between 50 and 75 years (inclusive)
- Have a significant response to DA therapies as judged by the Principal Investigator (PI) or other delegated clinician
- Have symptoms that are not appropriately controlled by existing oral anti-PD medications, as judged by the PI or other delegated clinician
- Ability to travel to Lund for surgery
- Followed up for at least 12 months prior to inclusion in this trial in the TransEUro observational study (reference: UK Research Ethics Committee (REC): 10/H0304/77; Swedish Ethical Review Authority (Etikprövningsmyndigheten) (EPM): Lund 2011/290, 2014/877 and 2019-06529)
- Be fluent in English/Swedish to enable completion of questionnaires as assessed by the PI or other delegated clinician at Cambridge/Lund, respectively
- Be approved by the TMG clinical sub-group for trial participation

9.2 Exclusion criteria

The presence of any of the following will preclude participant inclusion:

- Tremor dominant disease, as assessed by the PI or other delegated clinician
- Significant drug induced dyskinesias as defined by a score of > 2 in the Abnormal Involuntary Movement Scale (AIMS) dyskinesias rating scale, in any body part in the ON state
- Ongoing major medical or psychiatric disorders, including depression (Montgomery-Åsberg Depression Rating Scale [MADRS] > 20) and psychosis, that make participation unsuitable, as judged by the PI or other delegated clinician
- Any contraindication to neurosurgery
- Unable to be imaged using MRI
- Extensive ventral striatal loss or normal findings on F-DOPA PET at screening
- Significant cognitive impairment indicative of an incipient dementia/established dementia or values consistent with Montreal Cognitive Assessment (MoCA) score of ≤ 24
- Unable to perform normal copying of interlocking pentagons and/or a semantic fluency score for naming animals of less than 20 over 90 seconds
- Other concomitant treatment with neuroleptics (including atypical neuroleptics) and/or cholinesterase inhibitors
- Previous neurosurgery to the brain, or cell or organ transplantation, or recipient of repeated blood transfusions
- Any contraindication to immunosuppressive therapy, prophylactic antibiotics and/or osteoporosis prophylaxis (refer to STEM-PD Trial Immunosuppressant Manual)
- High levels of pre-formed specific anti-HLA antibodies to the cell product
- Severely reduced thiopurine methyltransferase (TPMT) activity (less than half of the lower normal TPMT activity level)
- History of documented severe/significant allergy requiring treatment
- Female who is pregnant or breastfeeding

- Received an investigational drug (including investigational vaccines) or used an invasive investigational medical device within 4 weeks of the screening visit, or is currently enrolled in an interventional investigational trial
- Female of childbearing potential or male unwilling to follow contraception requirements (see protocol section 12.16)
- Any other condition which, in the opinion of the investigator, makes the patient inappropriate for entry into the trial

9.3 Participant withdrawal criteria

9.3.1 Participant decision to withdraw

A participant can withdraw consent from any trial-related procedure or from the trial entirely at any time during the trial, without having to provide any justification, and this will not affect their standard of care.

Any participant who withdraws prior to surgery will be replaced to ensure 8 participants are transplanted. There will be no additional follow-up for participants withdrawing prior to surgery.

Any participant who withdraws post-surgery will be asked for consent to contact their neurologist/physician to allow us to monitor for the emergence of any potential side effects of the surgery and transplant, as well as asking for consent for using clinical data concerning their PD. These individuals will also be given contact details of the trial team in the event any problems arise relating to their participation in this trial.

Any data collected and samples already obtained will be included in the data reported for the trial.

9.3.2 Investigator withdrawal of participant

The investigator may withdraw a participant from the trial prior to surgery if a participant develops AEs, which, in the judgement of the investigator, would preclude the participant from transplantation, or develop medical issues that constitute a contraindication to inclusion. Investigators will not withdraw participants from the trial post-surgery.

10 Trial Treatments and Transplantation

10.1 Treatment summary

The STEM-PD product is classified as an ATIMP.

10.1.1 STEM-PD product

The STEM-PD product is a cryopreserved cell therapy product consisting of ventral midbrain dopaminergic progenitor cells derived from the clinical-grade hESC line (RC17) produced according to GMP.

10.1.1.1 *Legal status*

The trial is being carried out under a clinical trial authorisation (CTA). The product is therefore only to be used by the named investigators, for the participants specified in this protocol, and within the trial.

10.1.1.2 *Supply*

The STEM-PD product is owned by Lund University (LU) and manufactured by Royal Free Hospital (RFH), Centre for Cell, Gene & Tissue Therapeutics (CCGTT), London.

The ATIMP will be provided free of charge to the trial site. The STEM-PD product will only be dispatched to the site after receipt of confirmation from the STEM-PD trial coordinator that all greenlight regulatory documents are in place. Each shipment will contain sufficient vials for the transplantation of at least one participant, including back-up vials. Refer to the STEM-PD Trial Pharmacy Manual (STEM-PD SOP9) for further details.

10.1.1.3 *Packing and labelling*

For shipping of the STEM-PD product to the trial site, vials removed from storage at the manufacturing site will be placed into a cryobox and the box containing the vials will then be added to a temperature-monitored dry shipper for transfer by courier to the clinical trial site. Each shipment will contain sufficient vials for the transplantation of at least one participant, including backup vials. The vials will be stored locally at the site in vapour-phase liquid nitrogen until administration to the participant.

To complement the information on the vial label, each shipment of the STEM-PD product vials will include documentation as follows:

- Product documentation containing identification of the product, the vial identification numbers (IDs) in the shipment, and the clinical trial ID
- Batch certificate for the STEM-PD product with the latest updated expiry date
- Certificate of analysis (CoA) for the product, also with the latest updated expiry date

This documentation will be added to the Investigator Site File (ISF) and Trial Master File (TMF) after correct receipt of each shipment, and the product vials will be stored at the clinical site until dispensing and administration to the participant.

On the day of transplantation, STEM-PD product vials will be retrieved from liquid nitrogen storage at site by a trained member of the trial team, for thawing, washing and resuspension to prepare the cells in the final formulation for participant administration. During this procedure, the product will be transferred to a new vial and prepared in its final formulation for intraputamenal administration. A label containing the participant identification number will be added to the vial with the final STEM-PD product formulation, which is used in neurosurgery theatre for administration.

10.1.1.4 *Storage conditions*

The STEM-PD product will be transported and stored in temperature-monitored liquid nitrogen between -135°C and -195°C. The STEM-PD product will be stored at the site in a facility fulfilling all national requirements according to the European Union (EU) tissue and cell directives. For further information, refer to the STEM-PD Trial Pharmacy Manual (STEM-PD SOP9).

10.1.1.5 *Preparing the STEM-PD product for surgery*

On the day of the surgery, the number of vials of STEM-PD product needed for the surgery will be thawed and prepared as described in the Preparation of STEM-PD Drug Product for Patient Administration standard operating procedure (SOP) (STEM-PD SOP1). For further information, refer to the STEM-PD Trial Pharmacy Manual (STEM-PD SOP9).

10.1.1.6 *Monitoring sterility of the STEM-PD product*

Samples from the clinical cell handling procedure will be sent for 14-day sterility testing on the day of surgery. Results from the 14-day sterility testing will be communicated to the PI in written form by ALS Mikrolab in Sollentuna once analysed. All sterility results

must be stored in the Pharmacy Site File (PSF) when received. In the case of detection of microbial growth post-surgery in a submitted sample, the responsible clinician and trial coordinator must be notified immediately. The responsible clinician will discuss the results with a microbiologist who will then advise on appropriate follow up to prevent infections in the participant. The trial coordinator must further immediately inform the relevant authorities of the event.

10.1.1.7 *Maximum duration of treatment of a participant*

Participants will receive bilateral transplantation of the STEM-PD product during a single surgical session (Day 0) using the R-L instrument. The bilateral procedure is expected to take up to 10 hours.

10.1.1.8 *Dose*

Doses are expressed as the expected number of surviving DA neurons per putamen. For detailed information on dose calculation, refer to IB, section 6.2: Planned dose and method of administration.

Participants will receive either:

Dose 1:

Target yield:	100,000 surviving TH+ neurons per putamen
Total transplanted dose per putamen:	3.54×10^6 STEM-PD cells
Total transplanted dose per patient:	7.08×10^6 STEM-PD cells
Total number of deposits per putamen:	20
Volume per deposit:	2.5 µl
Total volume per putamen:	50 µl
Number of tracts per putamen:	5
Number of deposits per tract:	4
Target concentration of cell suspension:	70,800 cells/µl

Dose 2:

Target yield:	200,000 surviving TH+ neurons per putamen
Total transplanted dose per putamen:	7.08×10^6 STEM-PD cells grafted per putamen
Total transplanted dose per patient:	1.42×10^7 STEM-PD cells
Total number of deposits per putamen:	40
Volume per deposit:	2.5 µl
Total volume per putamen:	100 µl
Number of tracts per putamen:	5
Number of deposits per tract:	8
Target concentration of cell suspension:	70,800 cells/µl

The first four participants will receive dose 1, after which the imaging and clinical data will be reviewed by the independent DSMB, in conjunction with the clinical sub-group of the TMG. A decision will then be made as to the dosing for the next four participants. Refer to section 19.2.1 for overview of dose changing decision.

In summary, assuming there are no concerns either clinically or on imaging in all four participants treated with dose 1, and it is in line with the DSMB recommendation and final decision of the clinical sub-group of the TMG, the next four participants will receive dose 2.

10.1.1.9 *Dosing schedule*

No two participants will receive the transplantation on the same day. There will be gaps of at least 1 week between the participants as outlined in the table and text below (also refer to Appendix III). After the first participant transplant, there will be a delay of least

one month to assess any acute perioperative problems and/or safety signals, before the TMG clinical sub-group make the decision to transplant the second participant. Following the second transplant there will be a delay of 3 months to wait for the 3-month post-transplantation MRI assessment, which will provide important information on how the cells are behaving in the grafted brain especially with respect to any growth. MRI imaging and safety data will be considered by the TMG clinical sub-group before deciding to proceed with the transplantations of participants 3 and 4, which will take place at least one week apart.

After the fourth participant transplant there will be a delay of at least 6 months to collect participant safety and imaging data which will inform dosing decision for participants 5-8 (see sections 19.1.1.2 and 19.2.1). The dosing of participants 5-8 will follow the same schedule as for participants 1-4 if the decision is taken to move to dose 2.

**Anticipated timeline**	**Planned participants**	**Comments**<br>The TMG clinical sub-group will take into consideration safety signals from all participants and surgical procedure before proceeding with the next transplant
Month 0	Participant 1	TMG clinical sub-group assess screening results of participant 1 before approving to proceed with transplantation for participant 1
Month 1	Participant 2	One month after the transplant for participant 1, TMG assess safety signals from participant 1<br><br>TMG clinical sub-group review screening results of participant 2 before approving to proceed with transplantation for participant 2
Month 4	Participant 3<br><br>Participant 4	TMG clinical sub-group review safety data for participants 1 & 2 and MRI imaging for participants 1 and 2 for levels of proliferation<br><br>TMG clinical sub-group review screening results of participants 3 and 4 before approving transplantations for participants 3 and 4<br><br>Transplantations will take place at least one week apart
Month 10		DSMB assess minimum 6-month data for transplanted participants 1-4 and make a recommendation of how to proceed.<br><br>TMG clinical sub-group make final decision on dosing for participants 5-8.
Month 11*	Participant 5	TMG clinical sub-group assess screening results of participant 5 before approving to proceed with transplantation for participant 5
Month 12*	Participant 6	One month after the transplant for participant 5, TMG assess safety signals from participant 5

**Anticipated timeline**	**Planned participants**	**Comments**<br>The TMG clinical sub-group will take into consideration safety signals from all participants and surgical procedure before proceeding with the next transplant
		TMG clinical sub-group review screening results of participant 2 before approving to proceed with transplantation for participant 6
Month 15*	Participant 7<br><br>Participant 8	TMG clinical sub-group review MRI for participants 5 and 6 for levels of proliferation<br><br>TMG clinical sub-group review screening results of participants 7 and 8 before approving transplantations for participants 7 and 8<br><br>Transplantations will take place at least one week apart

* Note: The timelines for participants 5-8 are based on the assumption that the decision is taken at Month 10 by the TMG clinical sub-group to proceed with dose 2 at this time.
If the decision at Month 10 is to wait for further data on the first four participants (who received dose 1) (e.g. to 12 months post-transplant), the timelines will be adjusted accordingly.
If the decision at Month 10 is for participants 5-8 to receive dose 1 (and not proceed to dose 2), there will be only a minimum gap of one week between each transplantation.

10.1.1.10 *Surgical procedure for transplantation*

Refer to the STEM-PD Trial Surgical Manual (STEM-PD SOP2) for a detailed description.

A surgical planning MRI and CT angiogram will be performed prior to the day of surgery, as fully outlined in section 12.13.3.1.

Participants will be admitted to hospital the day before the planned surgery. Assessments will be performed the day before surgery, as outlined in the schedule of assessments (refer to section 12.12).

Surgery is performed under general anaesthesia. The actual use of anaesthetic will depend on the local standards and processes. An anaesthesiologist assessment will be performed prior to the day of surgery, in line with routine processes for neurosurgery.

The head preparation allows for either complete head shave or minimal shaving. This will be discussed with the participant prior to the surgery, and the participant will be asked for their preference.

The standard stereotactic frame for head fixation will be used, as per local guidelines. On the day of surgery, a stereotactic MRI (with the frame on the head) will be performed and fused with the previous surgical planning MRI to obtain the coordinates for the procedure.

Data will be recorded on surgical planning, including (not exclusively) co-ordinates and trajectories, to allow appraisal of targeting and implantation accuracy.

In the event where an interruption to the surgery between the two hemispheres is necessary, the TMG clinical sub-group will discuss how to proceed including which pre-surgery assessments will and won't be repeated. Should the clinical sub-group decide to

progress with the second hemisphere transplant, the interval between completion of the surgeries from the first to the second side should not exceed 1 month, however ideally this will be completed within 3 days (72 hours), provided the participant has received the immunosuppressive therapy according to the protocol. In this event, immunosuppressants and other nIMPs will be started based on the timing of the first surgery, with an additional dose of IV methyl prednisolone sodium succinate at the time of the second surgery. Additionally, all follow-up visits and cessation of nIMPs will be calculated from the timing of the second surgery, including the second basiliximab dose which will be given 4 days after the second surgery.

10.1.1.11 *Administration*

For full details around the administration of the STEM-PD product, refer to STEM-PD Trial Surgical Manual (STEM-PD SOP2).

In the event that the PI is unable to be present for the transplant procedure, another investigator may present. This may be the Sponsor-Investigator, but in this instance they will be performing this role as an investigator rather than in their capacity as Sponsor.

The administration device to be used in the transplant is the R-L device (see section 11).

The procedure will be a single-stage bilateral transplant using stereotactic image guided targeting, with one or more burr holes per hemisphere. Sulci/ventricles and vessels will be avoided in trajectory planning. To minimise any brain shift, the participant will be operated on in the neutral supine position and the dural incision will be limited.

Five trajectories per putamen will be made. Up to eight deposits of 2.5 μL each will be made along each trajectory giving a total of 20 deposits for dose 1 (= 50 μL) and 40 deposits for dose 2 (= 100 μL) per putamen.

To prevent reflux of cells around the delivery cannula, a waiting time after each deposit will be implemented, as described in detail in the STEM-PD Trial Surgical Manual (STEM-PD SOP2). Each trajectory will take approximately 45 minutes. The bilateral procedure is expected to take up to 10 hours.

The operation will end by closing the wound using local standard procedures. To minimise the risk of infection there will be no filling of the burr holes. After termination of immunosuppressive treatment, the burr holes can be filled if the participant requests this. For Cambridge participants, this procedure can be completed within the UK.

10.1.1.12 *Cell proliferation post grafting*

When transplanted, 66% of the STEM-PD cells are actively cycling. Three months post-transplant, this is reduced to 1.1% and 0.4% after 6 months. When interpreting participant's safety MRIs post-transplant, the clinical sub-group of the TMG should therefore consider that active proliferation of the STEM-PD cells ceases some time between 8 and 12 weeks.

10.1.1.13 *Known drug reactions*

The only possible interaction with the STEM-PD product would be the use of major anti-mitotic drugs as used in cancer, which could impair survival of dividing cells in the graft. Therefore, the use of major anti-mitotic drugs will be avoided in the first three months following completion of the transplant unless otherwise medically indicated.

There are restrictions in place for concomitant medication use during the trial due to the use of nIMPs and the surgical procedure. For further information, see section 10.3.

10.1.1.14 *Dose modifications*

Not applicable.

10.1.1.15 *Placebo/active comparator products*

There are no placebo/active comparator products to be used in this trial.

10.2 Non-investigational medicinal products (nIMPs)

10.2.1 Antibiotics

10.2.1.1 *Prophylactic antibiotics at the time of surgery*

Prophylactic antibiotics will be used as per local procedures at the time of surgery and for up to 48 hours post-transplant, as per local routine practice. Refer to the STEM-PD Trial Surgical Manual (STEM-PD SOP2) for further information.

10.2.1.2 *Trimethoprim-sulfamethoxazole*

Trimethoprim-sulfamethoxazole (also known as co-trimoxazole), in a ratio of 1:5, will be given as prophylaxis post-operatively, 3 times per week for the entire duration of the immunotherapy. Formulation to be used can be any available product on the market at each respective site.

10.2.2 Immunosuppressive treatment

Immunosuppressive treatment will start the day before the planned transplantation and will be continued for at least 12 months post-transplant (plus a tapering period). Full details are described in the STEM-PD Trial Immunosuppressant Manual (STEM-PD SOP3). Some of the immunosuppressive treatments required in the trial may affect the effectiveness of certain contraceptive methods. Where the effectiveness of a contraceptive method will be at risk due to an interaction with a trial nIMP, the local organ transplantation team will be consulted for advice.

Treatment will consist of the following combination of products.

10.2.2.1 *IV methyl prednisolone sodium succinate*

1 g IV methyl prednisolone sodium succinate is given as a one-off dose at the time of surgery. All formulations on the market are allowed.

10.2.2.2 *Tacrolimus*

Tacrolimus must be used as a formulation that is given twice a day (i.e., not a depot product). Approximately 0.05 mg/kg twice daily to commence the day before transplantation and for 12 months (plus tapering period as described in the STEM-PD Trial Immunosuppressant Manual, STEM-PD SOP3). Dosing will be based on an individual's measurement of blood concentrations. Refer to the STEM-PD Trial Immunosuppressant Manual (STEM-PD SOP3) for target concentrations. Brand switching for tacrolimus is not permitted in the trial and therefore single brand prescribing will be adopted for this medication.

If a recipient does not tolerate tacrolimus, an alternative to tacrolimus will be used (see section 10.2.2.3).

10.2.2.3 *Alternatives to tacrolimus*

In the event tacrolimus is not tolerated, participants will be commenced on ciclosporin. Ciclosporin will be used at a dose of 2 mg/kg twice daily, to commence the day before transplantation for 12 months (plus tapering period as outlined in the STEM-PD Trial Immunosuppressant Manual, STEM-PD SOP3). Refer to the STEM-PD Trial Immunosuppressant Manual (STEM-PD SOP3) for target concentrations. Brand switching for ciclosporin is not permitted in the trial and therefore single brand prescribing will be adopted for this medication. For the purpose of this trial, Sandimmun Neoral® will be used. Note the UK brand equivalent of this medication is Neoral®.

10.2.2.4 *Azathioprine*

Azathioprine, 1 mg/kg once daily, will commence the day before transplantation and be given once a day for 12 months. Formulation to be used can be any available product on the market at each respective site.

If azathioprine is not tolerated at 1 mg/kg, the dose may be lowered at the PI's discretion. In the event that a participant does not tolerate a lowered dose of azathioprine, the azathioprine may be stopped altogether at the PI's discretion. If azathioprine is stopped, no replacement medication will be given for this.

10.2.2.5 *Prednisolone*

Prednisolone (up to 40mg per day given once a day) as specified in the STEM-PD Trial Immunosuppressant Manual, STEM-PD SOP3. Doses will be adjusted according to participant tolerance and gradually reduced to 10mg once a day from week 12 onwards at the latest.

10.2.2.6 *Basiliximab*

Basiliximab will be given at a dose of 20 mg IV at day 0 (day of surgery) and day 4 post-transplant. All formulations on the market are permitted.

10.2.3 Prophylaxis

During the treatment with corticosteroids (first year after surgery), osteoporosis prophylaxis will be given to start three days post-surgery in the form of alendronic acid, 70 mg once weekly, and calcium carbonate/vitamin D supplements daily, or equivalent therapies as locally approved.

Additionally, gastro-protection prophylaxis should also be considered whilst the participant is receiving treatment with corticosteroids. The medication to be given will be at the PI's discretion, based on any contraindications and and/or any concomitant medications that the participant may already be taking (e.g., if the participant is already taking a proton pump inhibitor then another medication may not be required). Where omeprazole or lansoprazole is given, the concentration of tacrolimus should be monitored according to STEM-PD SOP3.

10.2.4 Dispensing of nIMPs

All medication will be dispensed as per local procedures, and either given to participants during their inpatient stay, at their clinic appointment or sent to the participant at home after their clinic appointment. Medications dispensed in Lund to Cambridge participants will be labelled in English.

The site is responsible for ensuring there is sufficient stock available for the trial.

10.2.5 PET ligands

For the PET scans two radiolabelled ligands (F-DOPA and PE2i) will be used.

The F-DOPA will be produced at the Cyclotron Unit, RS-SUS and Invicro Imaging Facilities, Hammersmith Hospital Campus, London for the scans to be performed in Sweden and the UK, respectively.

The PE2i ligand will be produced at the Cyclotron Unit, RS-SUS.

10.2.6 L-dopa challenge agent

A challenge dose of soluble L-dopa with a dopa decarboxylase inhibitor is given, calculated as the participant's average dose (total 24 hours LED/number of dosages per 24 hour period - up to a maximum of 250 mg of L-dopa). The specific agent used will depend on what is available locally. For Sweden, Madopark Quick® will be used. For the UK, dispersible Madopar® will be used.

If there are supply issues with dispersible Madopark Quick® or Madopar®, equivalent doses of non-soluble Madopar® or Sinemet® may be used.

10.3 Concomitant therapy

No participation in another clinical treatment trial or trial of a new invasive medical device is allowed.

Participants are permitted to take their standard drug therapies during the trial and, if necessary, have routine DBS treatment for their PD subsequent to the transplant if clinically indicated. Medication will be adapted according to usual clinical practice. Medication at the start of the trial as well as changes will be recorded in the electronic case report form (eCRF).

DBS treatment can be considered in case of worsening PD and/or significant disabling GIDs that cannot be managed pharmacologically. A graft recipient with severe GID and DBS treatment will be regarded as having had a SAE, and subject to detailed evaluations. However, DBS does not preclude most evaluations in the trial because they can be done with the stimulator switched off.

At a time-point of 3 months prior to the scheduled surgery the patient's anti-PD medication will be kept unchanged and stable unless it is medically warranted to adjust the dosages.

For the purposes of this trial, any COVID-19 vaccine will be recorded as a simple concomitant medication.

10.3.1 Prohibited medications

Following transplantation, participants should avoid DA-blocking agents (as would normally be contraindicated in patients with PD). However, if medically warranted these may be permitted.

During the nIMP treatment period, the use of drugs that are known to interact with the nIMPs should be avoided. Refer to STEM-PD Trial Immunosuppressant Manual (STEM-PD SOP3) for further details.

10.3.2 Medication withdrawal prior to surgery

On the baseline visit preceding surgery, it must be determined which of the participant's routine medication(s) should be withdrawn and when. Refer to the STEM-PD Trial Surgical Manual (STEM-PD SOP2) for details.

10.4 Accountability and dispensing

10.4.1 Pharmacy responsibilities

All pharmacy aspects of the trial are the responsibility of the local site PI, who will delegate these responsibilities to the local pharmacist or other appropriately qualified person. This delegation of duties must be recorded on the delegation log. The PI or a delegated individual (e.g. trial pharmacist) must ensure, at the site where the activity is undertaken, that the trial ATIMP and nIMPs are stored and dispensed in accordance with local practice, applicable regulatory requirements, protocol and Pharmacy Manual (STEM-PD SOP9).

The PI (or delegate, e.g. trial pharmacist) at RS-SUS is fully responsible for the ATIMP at site.

Dispensing of medication will be delegated to the relevant pharmacy. Local staff training logs must be kept up to date and made available on request. The delegation of duties and pharmacy staff signature logs must be completed with details of all staff involved in handling and/or dispensing the ATIMP. For the Cambridge site only, staff training logs will not be maintained for Pharmacy staff only involved in the receipt, handling, and dispensing of the nIMPs and accountability records will not be kept for the nIMPs.

Pharmacy responsibilities are fully outlined in the STEM-PD Trial Pharmacy Manual (STEM-PD SOP9).

10.4.2 Drug accountability

Full accountability and traceability must be recorded for all trial ATIMPs, such that if a product needs to be recalled or regulatory inspection undertaken, batch-specific details of all ATIMPs issued can be identified. Records documenting receipt, handling, storage, dispensing, retrieval of unused ATIMPs and destruction/disposal at site as permitted must all be filed in the PSF.

Following termination of the trial and at the request of the STEM-PD trial coordinator (on behalf of the Sponsor), all unused ATIMPs will be accounted for and destroyed in accordance with local practice or transferred to LU for research. Refer to the STEM-PD Trial Pharmacy Manual (STEM-PD SOP9) for further details.

10.4.3 Returns and destruction

Any unused or expired ATIMP vials may be destroyed as biological waste according to local policy or transferred to LU for research purposes, after written Sponsor authorisation. Where vials are sent to LU for research purposes, they can no longer be used for clinical use. Refer to the STEM-PD Trial Pharmacy Manual (STEM-PD SOP9) for further details.

All previously dispensed, unused nIMPs should be returned by the participant to the trial team and destroyed via normal processes.

11 Administration Device

11.1 Device details

The R-L device is a non-CE marked class III neurosurgical medical device. The R-L device consists of a re-usable delivery system intended for use during stereotactic brain surgery to deliver cell suspensions for the treatment of Parkinson's disease. The R-L device system is comprised of:

- A R-L injection device
- A guide cannula and blunt stylet
- A loading station

The R-L device is intended to draw up cells immediately before implantation; no cells are stored in the device prior to use.

The R-L device is designed to be re-used between participants and will be cleaned/sterilised in accordance with local requirements.

The R-L instrument will be used with a commercially-available stereotactic positioning system which is CE marked and used within its intended purpose, and hence is not to be tested in this trial as part of a clinical investigation.

Refer to protocol section 27.1.1.6 and the IB for earlier experiences with the R-L device.

For information on the compatibility between the device and the STEM-PD product, refer to Rehncrona-Legradi STEM-PD compatibility report.

11.2 Legal status

The R-L device is a non-CE marked device. The device has been manufactured in-house by RS-SUS for use on RS-SUS patients only. There is no objective to place it on the market, and this trial is not investigating the device, hence the device does not require to be tested as part of a clinical investigation.

11.3 Device manufacturer

The device is manufactured by RS-SUS, who is also the legal manufacturer.

11.4 Device supply

The R-L device is supplied by RS-SUS for use exclusively at RS-SUS.

11.5 Storage

The R-L device will be stored in accordance with local requirements.

11.6 Device accountability

There is no requirement for formal device accountability records to be maintained for the R-L device.

12 Procedures and Assessments

Trial assessments will be completed within the visit windows detailed in this section. If necessary, assessments can be completed over multiple days.

12.1 Location for assessments

Surgery for all participants (recruited at both CUH and RS-SUS) will take place at RS-SUS. Participants will travel to Lund approximately 7 days in advance of the surgery

and will remain in Lund for approximately 5 days post-surgery. Therefore, the trial assessments at day 1 & day 2 post-surgery will take place at RS-SUS for all participants.

Imaging will be performed in the following locations:

- F-DOPA PET/CT – will be performed at the Department of Neurophysiology for all participants
- PE2i PET/CT – will be performed at the Department of Neurophysiology, RS-SUS for all participants
- Surgical planning MRI and CT angiogram – Department of Neuroradiology, RS-SUS for all participants
- MRI with frame – Department of Neuroradiology, RS-SUS for all participants
- Safety MRI
    - Initial safety MRI (at day 2 ± 24 hours post-transplant) will be performed at Department of Neuroradiology, RS-SUS for all participants
    - All other safety MRIs will be performed at the Department of Neuroradiology, RS-SUS for participants from RS-SUS, and Department of Radiology, Addenbrooke's Hospital or Wolfson Brain Imaging Centre (WBIC), University of Cambridge for participants from CUH
- Extended battery MRI – Department of Neuroradiology, RS-SUS for all participants

All other assessments and procedures will be performed locally at each of the recruiting sites. In Lund, assessments will be performed at the Department of Neurology, RS-SUS. In Cambridge, assessments will be performed at John van Geest Centre for Brain Repair (VGB) (Department of Clinical Neurosciences, University of Cambridge [UCAM]), Wellcome Clinical Research Facility, and Addenbrooke's Hospital, Cambridge.

12.2 Potential effect of coronavirus 2019 (COVID-19)

The situation with COVID-19 may change over time, and accordingly national and local guidance on testing and research will be followed. In the event of another lockdown or threat of increased risk of transmission associated with attending visits and as such where seeing participants in-person or face-to-face is limited or not allowed, some assessments may be conducted remotely (either via phone or videoconference) or via the posting of questionnaires/assessments to participants to complete and send back. Safety assessments will continue to be performed in-person where needed. Safety bloods will be performed in accordance with local hospital processes at the time, for example participants may be asked to have blood testing performed off-site.

For the purposes of this trial, any doses of a COVID-19 vaccine (including booster jabs) will be recorded as a simple concomitant medication.

12.3 Participant identification

Potential participants will be identified through the ongoing TransEUro observational study that was started in 2011 (reference: UK REC: 10/H0304/77; EPM: Lund 2011/290, 2014/877 and 2019-06529).

12.4 Consent

The informed consent form (ICF) will be approved by the REC (in the UK) and the EPM (in Sweden) and will be in compliance with Good Clinical Practice (GCP), local regulatory and legal requirements, and General Data Protection Regulation (GDPR). The investigator or designee will ensure that each trial participant is fully informed about the nature and objectives of the trial and possible risks associated with their participation.

The investigator will obtain written informed consent from each participant before any trial-specific activity is performed. The ICF used for this trial and any changes made during the course of this trial will be prospectively approved by the REC (in the UK) and the EPM (in Sweden). The site will retain the original of each participant's signed ICF and the participant will receive a copy.

Any new information that becomes available, which might affect the participant's willingness to continue participating in the trial, will be communicated to the participant as soon as possible. This will occur verbally over the phone, if appropriate, in writing by post or email, or at their next visit. If applicable, participants will be asked to re-consent.

12.5 Screening evaluation

12.5.1 Participant registration

Participants will be allocated a unique trial ID after signing the ICF. Participant's registration will be undertaken via the STEM-PD trial database.

12.5.2 Screening assessments (day -120 to day -7)

Trial specific assessments will only be conducted after the participant has given written informed consent. The following assessments will then be completed:

- Concomitant medication, including PD medication
- Height and weight
- Physical examination
- Demographics
- Medical history
- Years of education
- Smoking status
- AE assessment
- Serum pregnancy test (applicable only for women of childbearing potential)
- Blood samples: full blood count (FBC), urea and electrolytes (U&E), liver function tests (LFTs), coagulation tests (including activated partial prothromboplastin test [APTT] and prothrombin time [PT]), C-reactive protein (CRP), microbiology/serology, HLA antibody titres against donor tissue, glucose, HbA1c, blood lipids, TPMT
- Methicillin-resistant Staphylococcus aureus (MRSA) - for Cambridge participants only
- Imaging:
    - MRI (screening)
    - PET (F-DOPA)/CT
- Motor assessments:
    - Movement Disorder Society Unified Parkinson's Disease Rating Scale (MDS-UPDRS) part III (in ON and OFF state)
    - AIMS (in ON and OFF state)
    - MDS-UPDRS parts I, II and IV
- Cognitive assessments:
    - MoCA (in ON state)
    - Pentagon copying (in ON state)
    - Semantic (animal naming) fluency (in ON state)
- Psychiatric assessments:
    - MADRS
- Assessment of eligibility

12.6 TMG clinical sub-group review

Once all screening assessments have been performed and all other eligibility criteria have been confirmed by the site, the clinical sub-group of the TMG will convene to review the participant and make a decision on whether to proceed with transplant surgery. The objective of this review is to ensure that both sites agree to enrol the participant in the trial (i.e. ensuring the inclusion/exclusion criteria are applied in the same way).

The recruiting site PI will be responsible for presenting to the clinical sub-group and will provide details relevant to eligibility criteria. The baseline assessments and surgery will then be scheduled once the clinical sub-group has issued approval to proceed. Further details of this process are outlined in the STEM-PD TMG Terms of Reference.

12.7 Baseline assessments (day -60 to day -1)

After review of the MRI and PET scans and confirmation that the participant remains eligible for a transplant, an appointment will be made to attend within 60 days of surgery. Instructions will be given concerning a temporary stop of any relevant medication before surgery (if applicable), and in line with routine neurosurgical requirements.

The following assessments will be completed:

- Concomitant medication, including PD medication
- Weight
- Electrocardiogram (ECG)
- Vital signs
- Physical examination
- AE assessment
- Serum or urine pregnancy test (applicable only for women of childbearing potential)
- Research blood sample (**optional**)
- Lumbar puncture for CSF collection (**optional**) & accompanying blood samples
- Imaging:
    - MRI (surgical planning)
    - CT angiogram
    - MRI (extended battery)
    - PET (PE2i)/CT
- Motor assessments:
    - MDS-UPDRS part III (in ON and OFF state)
    - 9 hole peg test (in ON and OFF state)
    - Timed sit-stand-walk test (in ON and OFF state)
    - RUSH dyskinesia scale (in ON and OFF state)
    - AIMS (in ON and OFF state)
    - 30 second tap test (in ON and OFF state)
    - MDS-UPDRS parts I, II and IV
    - Collection of Hauser patient diary card
    - **Lund participants only:** Collection of Parkinson KinetiGraph® (PKG®)
    - L-dopa challenge test
- Cognitive assessments:
    - Hopkins verbal learning task-revised (HVLT-R) (in ON state)
    - Stroop test (in ON state)
    - Digit span test (in ON state)
    - Wechsler Adult Intelligence Scale (WAIS) similarities test (in ON state)
    - Boston naming task (in ON state)
- Psychiatric assessments:

  - MADRS
- Non-motor/QOL assessments:
  - Parkinson's Disease Questionnaire 39 (PDQ-39)
  - EQ-5D-5L
  - PD non motor symptom scale (NMSS)
- Provision of participant contact/alert card

12.8 Cambridge participants travelling to Lund for surgery

Participants recruited from CUH will travel to Lund for surgery at approximately day -7. The participants will undergo pre-surgical checks as per standard processes at RS-SUS, including meeting with the anaesthetist, trial surgeon and neurologist. The surgical planning MRI, CT angiogram, extended battery MRI and PE2i PET/CT will also be scheduled for this period.

A companion may accompany participants if they wish, and all travel, living and accommodation costs will be covered for the entire duration they remain in Lund.

Additionally, a member of the trial team from CUH and at least one of the neurosurgeons from CUH will travel to Lund prior to the surgery.

12.9 Day before surgery (day -1)

Participants will be admitted as an inpatient at RS-SUS in preparation for surgery. The following assessments will be completed:

- Concomitant medication, including PD medication
- Weight
- Serum or urine pregnancy test (applicable only for women of childbearing potential) (will not need to be repeated on day -1 if performed within the 3 days prior to this visit, i.e. to day -4)
- Routine blood tests: FBC, U&E, LFTs, coagulation tests, CRP, cross match testing, glucose, HbA1c, blood lipids (will not need to be repeated on day -1 if performed within the 3 days prior to this visit, i.e. to day -4)

The relevant nIMPs will also be commenced, as outlined in section 10.2.

12.10 Day of surgery (day 0)

The following assessments will be completed and must be performed prior to surgery:

- Concomitant medication, including PD medication
- Vital signs
- AE assessment
- Imaging: MRI (with frame)

Once completed, participants will then proceed to theatre for transplantation of the STEM-PD product.

12.11 Post-surgery assessments

See schedule of assessments (section 12.12).

12.11.1 Timing of assessments

Assessments will be performed on day 1, and day 2 post-surgery. Further visits will take place at 7 days, 14 days, 21 days, 28 days, and 42 days following surgery. Participants will then be seen at 2 months, 3 months, 4 months, 5 months, 6 months, 9 months, 12 months, 18 months, 24 months, 30 months, and 36 months following

surgery. Participants may also be seen for an additional visit at 15+ months, dependent on the timing of stopping nIMPs (see section 12.11.14).

Acceptable windows around the planned visit time points are indicated in the relevant sections below.

Following the 36-month visit, participants will be invited, and are expected to participate in a separate follow-up study (see section 12.14).

12.11.2 Procedures: day 1 and day 2 post-surgery

The following assessments will be performed at day 1 and day 2 post-surgery.

- Concomitant medication (including PD medication)
- ECG (**day 2 only**)
- Vital signs
- AE assessment
- Blood tests:
  - FBC, U&E, tacrolimus/ciclosporin level, LFTs, CRP, glucose
  - **Day 1 only**: plus blood lipidsp
- Imaging: MRI **(**safety; **day 2 only (± 24 hours))**

These assessments will be performed at RS-SUS for all participants. Participants from the UK are expected to travel back to the UK approximately 5 days post-surgery, assuming they are clinically fit to do so.

12.11.3 Procedures: 7, 14, 21, 28, 42 days post-surgery

The following assessments will be performed at days 7, 14, 21, 28 and 42 post-surgery (± 2 days).

- Concomitant medication, including PD medication
- Weight
- Vital signs
- Physical examination
- AE assessment
- Serum or urine pregnancy test (for female participants of childbearing potential) (**day 28 only**)
- Review nIMP compliance
- Blood tests:
  - **All visits**: FBC, U&E, tacrolimus/ciclosporin level, LFTs, CRP, glucose
  - **Days 7, 14, and 28 only**: plus blood lipids
- Imaging: MRI **(**safety; **day 28 only)**
- Psychiatric assessments **(day 28 only)**:
  - MADRS

12.11.4 Procedures: 2 months post-surgery

The following assessments will be performed at 2 months post-surgery (± 1 week).

- Concomitant medication, including PD medication
- Weight
- Vital signs
- Physical examination
- AE assessment
- Serum or urine pregnancy test (for female participants of childbearing potential)
- Review nIMP compliance
- Blood tests: FBC, U&E, tacrolimus/ciclosporin level, LFTs, CRP, glucose, blood lipids
- Imaging: MRI (safety)

12.11.5 Procedures: 3 months post-surgery

The following assessments will be performed at 3 months post-surgery (± 1 week).

- Concomitant medication, including PD medication
- Weight
- Vital signs
- Physical examination
- AE assessment
- Serum or urine pregnancy test (for female participants of childbearing potential)
- Review nIMP compliance
- Blood tests: FBC, U&E, tacrolimus/ciclosporin level, LFTs, CRP, HLA antibody titres against donor tissue, glucose, HbA1c, blood lipids
- Imaging: MRI (safety)
- Motor assessments:
  - MDS-UPDRS part III (in ON state)
  - 9 hole peg test (in ON state)
  - Timed sit-stand-walk test (in ON state)
  - RUSH dyskinesia scale (in ON state)
  - AIMS (in ON state)
  - 30 second tap test (in ON state)
  - MDS-UPDRS parts I, II and IV
  - Collection of Hauser patient diary card
  - **Lund participants only:** Collection of PKG®
- Psychiatric assessments:
  - MADRS

12.11.6 Procedures: 4 months post-surgery

The following assessments will be performed at 4 months post-surgery (± 1 week).

- Concomitant medication, including PD medication
- Weight
- Vital signs
- Physical examination
- AE assessment
- Serum or urine pregnancy test (for female participants of childbearing potential)
- Review nIMP compliance
- Blood tests: FBC, U&E, tacrolimus/ciclosporin level, LFTs, CRP, glucose

12.11.7 Procedures: 5 months post-surgery

The following assessments will be performed at 5 months post-surgery (± 1 week).

- Concomitant medication, including PD medication
- Weight
- Vital signs
- Physical examination
- AE assessment
- Serum or urine pregnancy test (for female participants of childbearing potential)
- Review nIMP compliance
- Blood tests: FBC, U&E, tacrolimus/ciclosporin level, LFTs, CRP, glucose

12.11.8 Procedures: 6 months post-surgery

The following assessments will be performed at 6 months post-surgery (± 1 week). The PET and MRI imaging will have an allowed window of ± 1 month.

- Concomitant medication, including PD medication
- Weight
- Vital signs

- Physical examination
- AE assessment
- Serum or urine pregnancy test (for female participants of childbearing potential)
- Review nIMP compliance
- Blood tests: FBC, U&E, tacrolimus/ciclosporin level, LFTs, CRP, HLA antibody titres against donor tissue, glucose, HbA1c, blood lipids
- Imaging:
  - MRI (extended battery) **optional for Cambridge participants**
  - PET (F-DOPA)/CT: **optional for Cambridge participants**
  - MRI (safety)
- Motor assessments:
  - MDS-UPDRS part III (in ON and OFF state)
  - 9 hole peg test (in ON and OFF state)
  - Timed sit-stand-walk test (in ON and OFF state)
  - RUSH dyskinesia scale (in ON and OFF state)
  - AIMS (in ON and OFF state)
  - 30 second tap test (in ON and OFF state)
  - MDS-UPDRS parts I, II and IV
  - Collection of Hauser patient diary card
  - **Lund participants only:** Collection of PKG®
  - L-dopa challenge test
- Cognitive assessments:
  - MoCA (in ON state)
- Psychiatric assessments:
  - MADRS
- **Optional** lumbar puncture & accompanying blood samples
- **Optional** research blood sample

12.11.9 Procedures: 7 and 8 months post-surgery

For female participants of childbearing potential only a urine pregnancy test will be completed by the participants at home at 7 and 8 months post-surgery (± 1 week). A member of the trial team will contact the participant to confirm this has been done and record the result.

12.11.10 Procedures: 9 months post-surgery

The following assessments will be performed at 9 months post-surgery (± 1 week). MRI imaging will have an allowed window of ± 1 month.

- Concomitant medication, including PD medication
- Weight
- Vital signs
- Physical examination
- AE assessment
- Serum or urine pregnancy test (for female participants of childbearing potential)
- Review nIMP compliance
- Blood tests: FBC, U&E, tacrolimus/ciclosporin level, LFTs, CRP, HLA antibody titres against donor tissue, glucose, HbA1c, blood lipids
- Imaging: MRI (safety)
- Motor assessments:
  - MDS-UPDRS part III (in ON state)
  - 9 hole peg test (in ON state)
  - Timed sit-stand-walk test (in ON state)
  - RUSH dyskinesia scale (in ON state)
  - AIMS (in ON state)
  - 30 second tap test (in ON state)

  - MDS-UPDRS part I, II and IV
  - Collection of Hauser patient diary card
  - **Lund participants only:** Collection of PKG®

12.11.11 Procedures: 10 and 11 months post-surgery

For female participants of childbearing potential only a urine pregnancy test will be completed by the participants at home at 10 and 11 months post-surgery (± 1 week). A member of the trial team will contact the participant to confirm this has been done and record the result.

12.11.12 Procedures: 12 months post-surgery

The following assessments will be performed at 12 months post-surgery (± 1 week). The PET and MRI imaging will have an allowed window of ± 1 month.

- Concomitant medication, including PD medication
- Weight
- Vital signs
- Physical examination
- AE assessment
- Serum or urine pregnancy test (for female participants of childbearing potential)
- Review nIMP compliance
- Blood tests: FBC, U&E, tacrolimus/ciclosporin level, LFTs, CRP, HLA antibody titres against donor tissue, glucose, HbA1c, blood lipids
- Imaging:
  - MRI (extended battery): **optional for Cambridge participants**
  - MRI (safety)
  - PET (F-DOPA)/CT
  - PET (PE2i)/CT: **optional for Cambridge participants**
- Motor assessments:
  - MDS-UPDRS part III (in ON and OFF state)
  - 9 hole peg test (in ON and OFF state)
  - Timed sit-stand-walk test (in ON and OFF state)
  - RUSH dyskinesia scale (in ON and OFF state)
  - AIMS (in ON and OFF state)
  - 30 second tap test (in ON and OFF state)
  - MDS-UPDRS parts I, II and IV
  - Collection of Hauser patient diary card
  - **Lund participants only:** Collection of PKG®
  - L-dopa challenge test
- Cognitive assessments:
  - MoCA (in ON state)
- Psychiatric assessments:
  - MADRS
- Non-motor/QOL assessments:
  - PDQ-39
  - EQ-5D-5L
  - PD NMSS

12.11.13 Procedures: 13, 14, 15, 16 and 17 months post-surgery

For female participants of childbearing potential only a urine pregnancy test will be completed by the participants at home at 13, 14, 15, 16, and 17 months post-surgery (± 1 week). A member of the trial team will contact the participant to confirm this has been done and record the result.

12.11.14 Procedures: 15+ months post-surgery

The 15+ month visit will take place after at least 15 months post-surgery and within two weeks of the participant coming off their immunosuppressants. However, if this occurs within 2 weeks of the scheduled 18 month post-surgery visit, then this visit and the assessments listed below will not take place:

- Concomitant medication (including PD medication)
- Weight
- Vital signs
- AE assessment
- Review nIMP compliance

12.11.15 Procedures: 18 months post-surgery

The following assessments will be performed at 18 months post-surgery (± 2 weeks).

- Concomitant medication, including PD medication
- Vital signs
- Physical examination
- AE assessment
- Serum or urine pregnancy test (for female participants of childbearing potential)
- Review nIMP compliance
- Blood tests: HLA antibody titres against donor tissue, glucose, HbA1c, blood lipids
- Research blood sample (**optional**)
- Lumbar puncture for CSF collection & accompanying blood sample, where an appropriate sample has not already been collected (**optional**)
- Motor assessments:
    - MDS-UPDRS part III (in ON state)
    - 9 hole peg test (in ON state)
    - Timed sit-stand-walk test (in ON state)
    - RUSH dyskinesia scale (in ON state)
    - AIMS (in ON state)
    - 30 second tap test (in ON state)
    - MDS-UPDRS parts I, II and IV
    - Collection of Hauser patient diary card
    - **Lund participants only:** Collection of PKG®

12.11.16 Procedures: 24 months post-surgery

The following assessments will be performed at 24 months post-surgery (± 2 weeks). The PET and MRI imaging will have an allowed window of ± 1 month.

- Concomitant medication, including PD medication
- Vital signs
- Physical examination
- AE assessment
- Blood tests: HLA antibody titres against donor tissue
- Imaging:
    - MRI (extended battery) : **optional for Cambridge participants**
    - MRI (safety)
    - PET (F-DOPA)/CT : **optional for Cambridge participants**
    - PET (PE2i/CT): **optional for Cambridge participants**
- Motor assessments:
    - MDS-UPDRS part III (in ON and OFF state)
    - 9 hole peg test (in ON and OFF state)
    - Timed sit-stand-walk test (in ON and OFF state)
    - RUSH dyskinesia scale (in ON and OFF state)
    - AIMS (in ON and OFF state)

- 30 second tap test (in ON and OFF state)
- MDS-UPDRS part I, II and IV
- Collection of Hauser patient diary card
- **Lund participants only:** Collection of PKG®
- L-dopa challenge test

- Cognitive assessments:
  - MoCA (in ON state)
- Psychiatric assessments:
  - MADRS
- Non-motor/QOL assessments:
  - PDQ-39
  - EQ-5D-5L
  - PD NMSS

12.11.17 Procedures: 30 months post-surgery

The following assessments will be performed at 30 months post-surgery (± 2 weeks).

- Concomitant medication, including PD medication
- Vital signs
- Physical examination
- AE assessment
- Blood tests: HLA antibody titres against donor tissue
- Motor assessments:
  - MDS-UPDRS part III (in ON state)
  - 9 hole peg test (in ON state)
  - Timed sit-stand-walk test (in ON state)
  - RUSH dyskinesia scale (in ON state)
  - AIMS (in ON state)
  - 30 second tap test (in ON state)
  - MDS-UPDRS parts I, II and IV
  - Collection of Hauser patient diary card
  - **Lund participants only:** Collection of PKG®

12.11.18 Procedures: 36 months post-surgery

The following assessments will be performed at 36 months post-surgery (± 2 weeks). The PET and MRI imaging will have an allowed window of ± 1 month.

- Concomitant medication, including PD medication
- ECG
- Vital signs
- Physical examination
- AE assessment
- Blood tests: HLA antibody titres against donor tissue
- Research blood sample (**optional**)
- Imaging:
  - MRI (extended battery)
  - PET (F-DOPA)/ CT
  - PET (PE2i)/CT
- Motor assessments:
  - MDS-UPDRS part III (in ON and OFF state)
  - 9 hole peg test (in ON and OFF state)
  - Timed sit-stand-walk test (in ON and OFF state)
  - RUSH dyskinesia scale (in ON and OFF state)
  - AIMS (in ON and OFF state)
  - 30 second tap test (in ON and OFF state)
  - MDS-UPDRS parts I, II and IV

  - Collection of Hauser patient diary card
  - **Lund participants only:** Collection of PKG®
  - L-dopa challenge test
- Cognitive assessments:
  - MoCA (in ON state)
  - HVLT-R (in ON state)
  - Stroop test (in ON state)
  - Digit span test (in ON state)
  - WAIS similarities test (in ON state)
  - Boston naming task (in ON state)
- Psychiatric assessments:
  - MADRS
- Non-motor/QOL assessments:
  - PDQ-39
  - EQ-5D-5L
  - PD NMSS
- Enrol to long-term follow-up study

12.12 Schedule of assessments

Assessment	Screening	Baseline	Day before surgery	Surgery Day 0	Days							Months											
Visit	Day -120 to day -7	Day -60 to day -1			1	2	7	14	21	28	42	2	3	4	5	6	9	12	15+[12]	18	24	30	36
**GENERAL**																							
Informed consent	X																						
Assessment of eligibility	X																						
Concomitant medication (including PD medication)	X	X	X	X	X	X	X	X	X	X	X	X	X	X	X	X	X	X	X	X	X	X	X
Height	X																						
Weight	X	X	X				X	X	X	X	X	X	X	X	X	X	X	X	X				
ECG		X				X																	X
Vital signs		X		X	X	X	X	X	X	X	X	X	X	X	X	X	X	X	X	X	X	X	X
Physical examination	X	X					X	X	X	X	X	X	X	X	X	X	X	X		X	X	X	X
Demographics	X																						
Medical history	X																						
Years of education	X																						
Smoking status	X																						
AE assessment	X	X		X	X	X	X	X	X	X	X	X	X	X	X	X	X	X	X	X	X	X	X
Pregnancy test[1]	X	X	X							X		X	X	X	X	X	X	X		X			
Provision of participant contact/alert card		X																					
Review nIMP compliance							X	X	X	X	X	X	X	X	X	X	X	X	X	X			
Enrolment to long-term F/U																							X
**BIOLOGICAL SAMPLES**																							
FBC	X		X[3]		X	X	X	X	X	X	X	X	X	X	X	X	X	X					
U&E	X		X[3]		X	X	X	X	X	X	X	X	X	X	X	X	X	X					
Tacrolimus/ciclosporin level[2]					X	X	X	X	X	X	X	X	X	X	X	X	X	X					
LFTs	X		X[3]		X	X	X	X	X	X	X	X	X	X	X	X	X	X					
Coagulation tests	X		X[3]																				
CRP	X		X[3]		X	X	X	X	X	X	X	X	X	X	X	X	X	X					
Cross match testing			X[3]																				

Assessment	Screening	Baseline	Day before surgery	Surgery Day 0	Days							Months											
Visit	Day -120 to day -7	Day -60 to day -1			1	2	7	14	21	28	42	2	3	4	5	6	9	12	15+[12]	18	24	30	36
**BIOLOGICAL SAMPLES (continued)**																							
Microbiology/serology	X																						
HLA antibody titres[4]	X												X			X	X	X		X	X	X	X
Glucose	X		X[3]		X	X	X	X	X	X	X	X	X	X	X	X	X	X		X			
HbA1c	X		X[3]										X			X	X	X		X			
Blood lipids	X		X[3]		X		X	X		X		X	X			X	X	X		X			
TPMT	X																						
MRSA (Cambridge participants only)	X																						
Research blood sample (optional)		X														X				X			X
Lumbar puncture for CSF collection and accompanying blood sample[5] (optional)		X														X				X			
**IMAGING**																							
MRI (screening)	X																						
MRI (surgical planning)		X																					
CT angiogram		X																					
MRI (with frame)				X																			
MRI (safety)						X				X		X	X			X[6]	X	X[6]			X[6]		X[6]
MRI (extended battery)		X														X[7]		X[7]			X[7]		X
PET scan (F-DOPA)[8]	X															X[7]		X			X[7]		X
PET scan (PE2i)[8]		X																X[7]			X[7]		X
**MOTOR ASSESSMENTS**																							
MDS-UPDRS part III[9]	X ON + OFF	X ON + OFF											X ON			X ON + OFF	X ON	X ON + OFF		X ON	X ON + OFF	X ON	X ON + OFF
MDS-UPDRS parts I, II and IV	X	X											X			X	X	X		X	X	X	X
9 hole peg test[9]		X ON + OFF											X ON			X ON + OFF	X ON	X ON + OFF		X ON	X ON + OFF	X ON	X ON + OFF

Assessment	Screening	Baseline	Day before surgery	Surgery Day 0	Days							Months											
Visit	Day -120 to day -7	Day -60 to day -1			1	2	7	14	21	28	42	2	3	4	5	6	9	12	15+[12]	18	24	30	36
**MOTOR ASSESSMENTS (continued)**																							
Timed sit-stand-walk test[9]		X ON + OFF											X ON			X ON + OFF	X ON	X ON + OFF		X ON	X ON + OFF	X ON	X ON + OFF
Hauser patient diary card[10]		X											X			X	X	X		X	X	X	X
RUSH dyskinesia scale[9]		X ON + OFF											X ON			X ON + OFF	X ON	X ON + OFF		X ON	X ON + OFF	X ON	X ON + OFF
AIMS[9]	X ON + OFF	X ON + OFF											X ON			X ON + OFF	X ON	X ON + OFF		X ON	X ON + OFF	X ON	X ON + OFF
30 second tap test[9]		X ON + OFF											X ON			X ON + OFF	X ON	X ON + OFF		X ON	X ON + OFF	X ON	X ON + OFF
PKG®[10]		X											X			X	X	X		X	X	X	X
L-dopa challenge test[9]		X														X		X			X		X
**COGNITIVE ASSESSMENTS**																							
MoCA	X ON															X ON		X ON			X ON		X ON
Pentagon copying	X ON																						
Semantic (animal naming) fluency[11]	X ON																						
HVLT-R		X ON																					X ON
Stroop test		X ON																					X ON
Digit span test		X ON																					X ON
WAIS similarities test		X ON																					X ON
Boston naming task		X ON																					X ON
**PSYCHIATRIC ASSESSMENTS**																							
MADRS	X	X								X			X			X		X			X		X

Assessment	Screening	Baseline	Day before surgery	Surgery Day 0	Days							Months											
Visit	Day -120 to day -7	Day -60 to day -1			1	2	7	14	21	28	42	2	3	4	5	6	9	12	15+[12]	18	24	30	36
**NON-MOTOR/QOL ASSESSMENTS**																							
PDQ-39		X																X			X		X
EQ-5D-5L		X																X			X		X
PD NMSS		X																X			X		X
**TREATMENT**																							
Surgery (transplantation of STEM-PD product)				X																			
nIMPs			**As prescribed (including tapering)**																				

[1] To be performed for female participants of childbearing potential only. At screening, a serum pregnancy test will be performed, and on all subsequent visits either a serum or urine pregnancy test will be performed at site discretion. Monthly urine pregnancy tests will be performed by participants at home at months 7, 8, 10, 11, 13, 14, 15, 16 and 17. A member of the trial team will contact the participant to confirm the pregnancy test has been performed and record the result.
[2] To be performed depending on which immunosuppressant treatment the participant is on, i.e. if participant on tacrolimus treatment then tacrolimus level will be tested, and if on ciclosporin A the CyA level will be tested.
[3] Samples will not need to be repeated on day -1 if performed within the 3 days prior to this visit, i.e. to day -4). Blood typing does not need to be repeated.
[4] At screening, will include a screening test of anti-HLA antibodies. If positive, specific assessment of any major histocompatibility complex (MHC) class I antigens against those expressed in the STEM-PD product will be carried out. For all other visits, HLA antibodies will be monitored.
[5] Where possible, blood analysis will be performed on samples already collected.
[6] For visits completed in Lund, this will be done simultaneously with the extended battery MRI
[7] Optional for Cambridge participants.
[8] Low dose CT scans will be performed in conjunction with each PET scan performed at RS-SUS.
[9] To be videoed with the participant wearing a cap.
[10] Hauser patient diary cards and PKG® will be provided to participants ahead of the visit to enable completion of data prior to the visit. At the visits the trial team will collect in the Hauser patient diary cards and PKG®, as outlined in STEM-PD SOP4: Motor and Cognitive Assessments. PKG for Lund participants only.
[11] The semantic (animal naming) fluency assessment may also be videoed if required by the site to assist with scoring.
[12] The 15+ month visit will occur within two weeks of the participant completing their course of immunosuppressants. If the completion of immunosuppressants is within 2 weeks of the scheduled 18 month post-surgery visit, then this visit and the assessments listed will not take place.

12.13 Summary of assessments

12.13.1 General

12.13.1.1 *Concomitant medication, including PD medication*

All concomitant medications, including any PD medication, will be checked and changes recorded. Changes to PD medication will be evaluated and calculated as the LED (in mg) (Schade et al., 2020). Calculation of the LED will be performed as fully outlined in STEM-PD SOP4.

12.13.1.2 *Height, weight and vital signs*

Height (in metres) and weight (in kilograms) will be recorded.

Vital signs will be recorded and include: blood pressure, pulse, and body temperature.

12.13.1.3 *ECG*

A 12-lead ECG will be performed and standard measurements will be reviewed as per local routine practice. Any abnormalities will be recorded and managed as per local routine practice.

12.13.1.4 *Physical examination*

Will include a review of all body systems as per standard practice.

12.13.1.5 *Demographics*

Will include: sex, ethnicity, date of birth, and date of PD diagnosis.

12.13.1.6 *Medical history, years of education and smoking status*

Pre-existing medical conditions will be recorded at the screening visit. In the event that any pre-existing conditions worsen during the course of the trial, or new medical diagnoses occur, these will be recorded as AEs. Disease progression will only be recorded as an SAE if it is considered more severe than expected in the trial population.

Additionally, data will be collected for years of education and smoking status (never smoked, ex-smoker, and current smoker).

12.13.1.7 *Pregnancy testing*

Pregnancy tests will be performed only for female participants of childbearing potential. At the screening visit, a serum pregnancy test will be performed. At subsequent visits, and for up to 18 months post-surgery, serum or urine pregnancy tests will be performed on a monthly basis. Where this doesn't coincide with a trial visit, participants will take a urine pregnancy test at home. Participants will be contacted by a member of the trial team to confirm the results of the test.

12.13.2 Biological samples

12.13.2.1 *Full blood count (FBC)*

To include: white cell count, neutrophil count, lymphocyte count, platelet count, haemoglobin, blood haematocrit, and mean corpuscular volume.

12.13.2.2 *Urea and electrolytes (U&E)*

To include: urea, potassium, sodium, and creatinine.

12.13.2.3 *Tacrolimus/ciclosporin level*

Trough blood levels (12 hours) after last dose of tacrolimus/ciclosporin, and prior to the next dose be monitored. Participants on tacrolimus treatment will have tacrolimus levels monitored, and participants on ciclosporin will have ciclosporin levels monitored. Refer to the STEM-PD Trial Immunosuppressant Manual (STEM-PD SOP3) for target concentrations.

12.13.2.4 *Liver function tests (LFTs)*

To include: alanine transaminase (ALT), albumin, alkaline phosphatase (ALP), and total bilirubin.

12.13.2.5 *Coagulation test*

To include: APTT and PT.

12.13.2.6 *C-reactive protein (CRP)*

CRP levels will be measured.

12.13.2.7 *Cross match test*

Blood will be sent to the local laboratory, to include:

- Blood typing (blood group, rhesus status)
- BAS test (blood group control and antibody screening) / other tests in case a blood transfusion is needed, as per local pre-operative procedures.

Blood typing is routinely done before the MRI under anaesthesia as per local procedures.

12.13.2.8 *Glucose metabolism & blood lipids*

To include:

- HbA1c;
- Glucose will be performed non-fasting, and repeated as a fasting measurement if clinically indicated;
- Blood lipids, including cholesterol (total, low density lipoprotein [LDL] and high density lipoprotein [HDL]), and total lipids triglycerides. Blood lipids will also be performed non-fasting and repeated as a fasting measurement if clinically indicated.

12.13.2.9 *Microbiology/serology*

Participants will be tested for human immunodeficiency virus (HIV 1/2), hepatitis B (HBV), hepatitis C (HCV), human T-lymphotropic virus (HTLV) I+II, and treponema pallidum (and confirmed positive results for all agents are regarded as contraindication for immunotherapy, unless vaccinated for HBV, or treated and viral free for HCV). Participants will also be tested for the following opportunistic and neurotropic viruses: herpes simplex virus (HSV 1 and 2), varicellae zoster virus (VZV), John Cunningham (JC) virus and toxoplasma gondii. Serum Immunoglobulin G (IgG) and Immunoglobulin M (IgM) antibody titres will be performed for cytomegalovirus (CMV) and Epstein-Barr virus (EBV).

12.13.2.10 *HLA antibody titres against donor tissue*

At screening, participants will undergo a screening test of anti-HLA antibodies, and, if positive a specific assessment will be performed of any MHC class I antigens against those expressed in the STEM-PD product.

HLA antibodies will be monitored throughout the course of the trial.

12.13.2.11 *Thiopurine methyltransferase (TPMT)*

The TPMT level will be measured.

12.13.2.12 *MRSA*

To check for the presence of MRSA.

12.13.2.13 *Research blood sample– optional*

Optional research blood samples, which may include whole blood, DNA, plasma and serum samples, will be collected from participants who consent to this. Samples will be analysed for immunological responses and any remaining samples will be retained for future research (see section 16.2).

12.13.2.14 *Cerebrospinal fluid (CSF) collection – optional*

Optional lumbar punctures will be performed for participants who consented to these. CSF will be collected and local routine parameters measured such as cell count, protein and glucose levels, presence of oligoclonal bands of immunoglobulins. In case of oligoclonal band analyses an accompanying blood sample will also be taken (where an appropriate sample has not already been collected) to analyse blood glucose and oligoclonal bands. Analysis of routine parameters will be performed locally at each site.

The samples will also be analysed for immunological responses. The remainder of the samples will be stored for future ethically-approved research.

12.13.3 Imaging

For further information, refer to the STEM-PD Trial Imaging Manual at each imaging centre.

12.13.3.1 *MRI scan*

Participants will undergo different types of MRI during the course of the trial.

- **Screening MRI**: To check for any contraindications for inclusion. Contraindications for inclusion include evidence of structural changes that may affect the course of disease or ability to tolerate neurosurgical procedure, e.g. vascular malformations (arterio-venous malformations, haemangiomas, aneurysms) or other structural changes that may be of importance, as judged by the PI or clinical sub-group of the TMG.
- **Surgical planning MRI**: The MRI will be performed under general anaesthesia with added angiographic visualisation using either time of flight (TOF) or gadolinium contrast (refer to STEM-PD Trial Surgical Manual, STEM-PD SOP2). As with the surgery, this will be performed in the neutral supine position. In addition a CT angiogram will be performed to allow visualisation of the cerebral vessels in more detail.
- **MRI with frame**: To be performed on the day of surgery only, as this is a requirement for neurosurgery. The image from the MRI with frame will be registered with the previous surgical planning MRI to obtain the final frame coordinates for the procedure. This may be performed under local anaesthesia if the participant agrees to this: if not, this scan will also be performed under general anaesthesia.
- **Safety MRI**: These scans will be checked to monitor for any safety concerns following the transplant.
- **Extended battery MRI**: Participants will undergo multi-sequence MRI lasting no longer than 55 minutes. Participants will be in an OFF state for the extended battery scan. Where possible, this will be performed on the same day as the PET

scans which take place in Lund, as per the schedule of assessments. If it is not possible to complete all MRI sequences on the same day as the PET scan, the remaining MRI sequences may be performed the following day (in an ON or OFF state, at the discretion of the PI).

12.13.3.2 *PET scans*

Participants will undergo a PET protocol consisting of F-DOPA and PE2i scans.

F-DOPA: 185MBq [^{18}F]F-DOPA will be injected as a single intravenous bolus injection, with volumes made to 10 ml using saline and injected over 10 seconds (1 ml/second), followed by a 10 ml saline flush. Scanning will be performed 0-90 minutes post-injection. Data will be stored in LIST-mode (raw data) to allow for different reconstruction protocols to be used.

The F-DOPA scans are performed after being off L-dopa/catechol-o-methyl transferase (COMT) inhibitors for at least 12 hours or 24 hours for long-acting dopamine agonists/monoamine oxidase B (MAO-B) inhibitors, i.e. "practically defined OFF phase". Other medications interfering with F-DOPA imaging will be stopped at least 24 hours prior to PET scanning, or according to local recommendations. Carbidopa (150 mg) is administered orally with water 60 minutes prior to the scans. Participants are instructed to eat a low-protein breakfast and avoid a heavy meal on the day of the scanning. Participants are also instructed to avoid consumption of caffeine (including no coffee, tea or energy drinks), and to not smoke on the day of the scanning.

[^{18}F]FE-PE2i: 185MBq [^{18}F]FE-PE2i will be injected as an intravenous injection and scanning will be performed 0-75 minutes post-injection. Data will be stored in LIST-mode (raw data) to allow for different reconstruction protocols to be used. PE2i scans are also performed after being off medication, as described above for F-DOPA scans.

Low dose CT scans will be performed in conjunction with each PET scan performed at RS-SUS.

In the event of a delayed or cancelled surgery, the F-DOPA imaging will only be repeated if performed for more than 6 months before the new surgery date. PE2I scan will not be repeated. Other assessments will need to be repeated if they fall outside of the relevant screening or baseline window.

12.13.4 Motor assessments

For further information, refer to the STEM-PD Trial Motor and Cognitive Assessments SOP (STEM-PD SOP4).

12.13.4.1 *Videoing of motor assessments*

All motor assessments (as well as the semantic (animal naming) fluency assessment if the site requires this to assist with scoring) will be videoed to enable independent review of the assessments and validation of the scores. Participants will be required to wear a cap for the video assessments to ensure it is not possible for the independent reviewer to distinguish before and after surgery. This will be fully covered in the participant information sheet (PIS).

12.13.4.2 *ON and OFF testing*

Specified motor tasks will be performed in both ON and OFF states.

For the OFF state, participants will be asked to stop medication at least 12 hours prior to the visit (for L-dopa/COMT inhibitors) or at least 24 hours (for long-acting dopamine agonists/MAO-B inhibitors).

The ON state assessments will be performed when participants feel their medication has worked, which would be typically 60 to 90 minutes after taking their medications.

12.13.4.3 *Movement Disorder Society – Unified Parkinson's Disease Rating Scale (MDS-UPDRS)*

This is a scale which has sections to be completed by the person with PD together with their carers (if appropriate), as well as sections to be completed by the clinician.

Part I assesses the non-motor aspects of experiences of daily living in the week prior to the visit. Part II assesses the motor aspects of experiences of daily living in the week prior to the visit. Part III assesses the motor examination of a participant at the time of the visit (including the Hoehn and Yahr stage), and part IV assesses motor complications in the week prior to the visit. Each item will receive a score ranging from 0 to 4, where 0 represents the absence of impairment and 4 represents the highest degree of impairment.

12.13.4.4 *Nine-hole peg test*

The nine-hole peg test is used to measure finger dexterity in participants with neurological diagnoses. It is administered by asking the participant to take the pegs from a container, one by one, and placing them into the holes on the board as quickly as possible. Participants must then remove the pegs, one by one, and replace them into the container. Scores are based on the time taken to complete the test activity.

12.13.4.5 *Timed sit-stand-walk test*

The timed sit-stand-walk test is used as a measure of lower extremity strength and speed of walking. Participants begin the test sitting in a chair and told to come to a full stand, then walk 6 metres to a defined point, return to the chair and sit down. The time taken to complete this is measured.

12.13.4.6 *Hauser patient diary card*

The Hauser patient diary card is a 24-hour diary, divided into 30-minute sections. This is used to record PD symptoms and how much time is spent in the different motor states (ON without dyskinesia, ON with non-troublesome dyskinesias, ON with troublesome dyskinesias, OFF, or asleep). Participants will be provided with the diary cards prior to attending the visits and asked to complete this for 3 consecutive days. The completed diary cards will then be collected at the relevant visits.

12.13.4.7 *Parkinson KinetiGraph® (PKG®)*

The PKG® is a wrist worn accelerometer from Global Kinetics Pty Ltd. The PKG® watch is worn on the most affected side of the body (same side for all tests). Registration will be done for 7 consecutive days just before a visit for verification with the Hauser diary and tor the purposes of this trial, the following data will be collected: bradykinesia and dyskinesia index, OFF time (to correlate with the Hauser patient diary card), and registration of intake of anti-PD medications. Participants will be provided with the PKG® prior to attending the visits to complete data collection and will be asked to return the PKG® at the relevant visit. Only Lund participants will complete the PKG® assessments.

Once the electronic data is transferred from the device to a computer platform, it is processed to generate data.

12.13.4.8 *RUSH dyskinesia scale*

The RUSH dyskinesia scale is a scale to assess the severity of overall dyskinesias based on interference in activities in daily living, to distinguish chorea from dystonia (the two major types of dyskinesias in PD), and to identify the single most disabling form of dyskinesia.

12.13.4.9 *Abnormal Involuntary Movement Scale (AIMS)*

The AIMS is used to quantify dyskinesias in the arms, legs or head, and trunk, which includes items such as facial behaviours (jaw, facial muscles, tongue and lips), full-body behaviours (upper, lower, and trunk), and incapacitation due to these movements. The items are scored from 0 to 4, where 0 is normal/healthy and 4 is severely affected.

12.13.4.10 *30 second tap test*

The 30 second tap test is a dexterity assessment where the participant is asked to finger tap for 30 seconds. It is tested on both sides, as quickly as possible with the fingers fully open. The number of taps over 30 seconds is registered and the test is performed twice on each hand.

12.13.4.11 *Challenge test of levodopa*

The L-dopa challenge test will be performed after completion of the OFF motor assessments.

A challenge dose of L-dopa is given, calculated as the participant's average dose (total 24 hours LED/number of dosages per 24 hour period - up to a maximum of 250 mg of L-dopa) at the time of the baseline visit. The same dose will be used at baseline, 6 months and 12 months, with possible changes in doses used at 24 and 36 months based on their oral DA intake at these times.

The challenge dose is given after being off standard L-dopa/COMT inhibitors medications for at least 12 hours or at least 24 hours for long-acting dopamine agonists or L-Dopa preparations and for MAO-B inhibitors, i.e. "practically defined OFF phase". Participants are tested after a low protein breakfast, examples of which will be provided to the participants.

After taking the challenge dose, participants will wait for 30 minutes and then perform the MDS-UPDRS part III and the AIMS. These two assessments will then be repeated every 30 minutes up to 120 minutes after taking the challenge dose.

12.13.5 Cognitive assessments

For further information, refer to STEM-PD SOP4: Trial Motor and Cognitive Assessments.

12.13.5.1 *Montreal Cognitive Assessment (MoCA)*

The MoCA is a 30-question test of global cognitive function, which evaluates different types of cognitive abilities, including: orientation, short-term memory, executive function, visuospatial ability, language ability, abstraction, animal naming, and attention.

12.13.5.2 *Hopkins verbal learning task-revised (HVLT-R)*

The HVLT-R consists of a 12-item word list, composed of four words each from one of three semantic categories to be learned over the course of three immediate recall learning trials. Participants are also required to undergo a delayed recall trial (requiring free recall of any of the words remembered) and a recognition trial (composed of 24 words, including the 12 target words plus 12 false-positives).

12.13.5.3 *Semantic (animal naming) fluency*

Participants are asked to name as many animals as possible in 90 seconds. As outlined above for the motor assessments (section 12.13.4.1) this assessment may also be videoed to assist with scoring.

12.13.5.4 *Stroop test*

The Stroop test is a measure of verbal processing speed and response inhibition, consisting of three timed trials.

12.13.5.5 *Digit span test (forwards and backwards)*

Digit span is measured for forward and reverse-order recall of digit sequences.

12.13.5.6 *Boston naming task*

In the Boston naming task participants are shown a number of images and are asked to say what the image is.

12.13.5.7 *Wechsler Adult Intelligence Scale (WAIS) similarities test*

The WAIS similarities assessment includes 18 pairs of words, where participants are asked to name how they are alike or similar.

12.13.5.8 *Pentagon copying*

In the pentagon copying assessment, participants are asked to copy an intersecting double pentagon figure.

12.13.6 Psychiatric assessments

12.13.6.1 *Montgomery-Åsberg Depression Rating Scale (MADRS)*

The MADRS is a ten-item diagnostic questionnaire used to measure the severity of depressive episodes in patients. The questionnaire includes questions on the following symptoms: 1) apparent sadness; 2) reported sadness; 3) inner tension; 4) reduced sleep; 5) reduced appetite; 6) concentration difficulties; 7) lassitude; 8) inability to feel emotions; 9) pessimistic thoughts; and, 10) suicidal thoughts.

12.13.7 Non-motor/quality of life (QOL) questionnaires

12.13.7.1 *Parkinson's Disease Questionnaire 39 (PDQ-39)*

The PDQ-39 is a self-rated questionnaire designed to measure PD-related quality of life. It contains 39 questions, covering 8 aspects of quality of life: mobility, activities of daily living (ADL), emotions, stigma, social support, cognition, communication and bodily discomfort. It will be completed by the participant.

12.13.7.2 *EQ-5D-5L*

The EQ-5D-5L is a standardised instrument for measuring generic health status in terms of five dimensions: mobility, self-care, usual activities, pain/discomfort and anxiety/depression. It will be completed by the participant.

12.13.7.3 *PD non motor symptom scale (NMSS)*

The PD NMSS is a 30-point validated scale which is designed to quantify the extent and severity of non-motor symptoms of PD experienced by the patient. It will be completed by the delegated members of the local research team.

12.14 Long-term follow-up assessments

Participants will be followed-up until 36 months post-transplant as part of this protocol. Long-term follow up will be carried out as part of a separate protocol and ethics application.

12.15 End of trial participation

The end of trial participation will be 36 months post-transplant (or the last participant visit if withdrawn earlier). After 36 months post-transplant, participants will be expected to transfer into a separate long-term follow-up study (the expectation for this will be explained in the STEM-PD trial PIS and discussed with the participant as part of the consent process).

Any participant who withdraws post-surgery will be asked for consent to contact their neurologist/physician to allow us to monitor for the emergence of any potential side effects of the surgery and/or cell product.

12.16 Trial restrictions

For the purposes of this trial, a woman is considered of childbearing potential (i.e., fertile), following menarche and until becoming post-menopausal unless permanently sterile. Permanent sterilisation methods include hysterectomy, bilateral salpingectomy and bilateral oophorectomy.

A postmenopausal state is defined as no menses for 12 months without an alternative medical cause. A high follicle stimulating hormone (FSH) level in the postmenopausal range may be used to confirm a postmenopausal state in women not using hormonal contraception or hormonal replacement therapy. However in the absence of 12 months of amenorrhea, a single FSH measurement is insufficient.

Women of childbearing potential are required to use one highly effective method of contraception for the duration of the immunosuppressant treatment, plus an additional 3 months after completion of immunosuppressant treatment. Highly effective forms of contraception include:

- Combined (oestrogen and progestogen containing) hormonal contraception associated with inhibition of ovulation
    - Oral
    - Intravaginal
    - Transdermal
- Progestogen-only hormonal contraception associated with inhibition of ovulation
    - Oral
    - Injectable
    - Implantable
- Intrauterine device (IUD)
- Intrauterine hormonal-releasing system
- Bilateral tube occlusion

Contraception does not need to be used by women of childbearing potential when:

- The participant only has one partner, and the male partner has had a vasectomy
- The participant is unable to get pregnant
- The participant practices true abstinence (where this is in accordance with the participant’s preferred and usual lifestyle). If the participant becomes sexually active during the course of the trial, they must use contraception as outlined above.

Women should also avoid breast-feeding for the duration of the immunosuppressant treatment.

Men are required to use effective contraception for the entire duration of the immunosuppressant treatment. This includes:

- Barrier contraception (condom and spermicide) even if female partner(s) are using another method of contraception or are already pregnant (also to protect male partners from exposure to the trial nIMPs etc.)

Contraception does not need to be used by men when:

- The participant has had a vasectomy
- The participant only has one partner, and the female partner is unable to get pregnant
- The participant practices true abstinence (where this is in accordance with the participant's preferred and usual lifestyle). If the participant becomes sexually active during the course of the trial, they must use contraception as outlined above.

13 Assessment of Safety

Refer to the STEM-PD Trial Safety Management Plan for further information.

13.1 Definitions

13.1.1 Adverse event (AE)

Any untoward medical occurrence in a participant or clinical trial participant administered a medicinal product and which does not necessarily have a causal relationship with this treatment.

An AE can therefore be any unfavourable and unintended sign (including an abnormal laboratory finding), symptom, or disease temporally associated with the use of an investigational medicinal product (IMP), whether or not considered related to the IMP.

Please note: Recording of all AEs must start from the point of informed consent regardless of whether a participant has yet received a medicinal product.

All AEs will be coded using the Medical Dictionary for Regulatory Activities (MedDRA).

13.1.2 Adverse event of special interest (AESI)

An AE of special interest (AESI) (serious or non-serious) is one of scientific and medical concern specific to the Sponsor's product or programme, for which ongoing monitoring and rapid communication by the investigator to the Sponsor could be appropriate. Such an event might require further investigation in order to characterise and understand it. Depending on the nature of the event, rapid communication by the trial Sponsor to other parties (e.g. regulators) might also be warranted.

13.1.3 Adverse reaction to an investigational medicinal product (AR)

All untoward and unintended responses to an IMP related to any dose administered. All AEs judged by either the reporting investigator or the Sponsor as having a reasonable causal relationship to a medicinal product qualify as adverse reactions (ARs). The expression reasonable causal relationship means to convey in general that there is evidence or argument to suggest a causal relationship.

13.1.4 Unexpected adverse reaction

An AR, the nature, or severity of which is not consistent with the applicable reference safety information (RSI) (see section 13.1.7 for RSI for this trial).

When the outcome of the AR is not consistent with the applicable RSI this AR should be considered as unexpected.

The term "severe" is often used to describe the intensity (severity) of a specific event. This is not the same as "serious," which is based on participant/event outcome or action criteria.

13.1.5 Serious adverse event or serious adverse reaction (SAE/SAR)

Any untoward medical occurrence that at any dose:

- results in death;
- is life-threatening;
- requires hospitalisation or prolongation of existing in-patients' hospitalisation;
- results in persistent or significant disability or incapacity;
- is a congenital anomaly or birth defect; and/or,
- is an important medical event – some medical events may jeopardise the participant or may require an intervention to prevent one of the above characteristics/consequences. Such events (hereinafter referred to as 'important medical events') should also be considered as 'serious'.

Life-threatening in the definition of a SAE or serious AR (SAR) refers to an event in which the participant was at risk of death at the time of event; it does not refer to an event which hypothetically might have caused death if it were more severe.

13.1.6 Suspected unexpected serious adverse reaction (SUSAR)

A SAR, the nature and severity of which is not consistent with the information set out in the RSI.

An untoward and unintended response to a nIMP which does not result from a possible interaction with the ATIMP is, by definition, not a suspected unexpected SAR (SUSAR).

13.1.7 Reference safety information (RSI)

A list of medical events that defines which reactions are expected for the IMP within a given trial and thus determining which SARs require expedited reporting.

The RSI is contained in a clearly identified section of the IB for the ATIMP.

For this trial the RSI for the STEM-PD product is section 6.7.2 of the IB approved by the Medicines and Healthcare products Regulatory Agency (MHRA) and Swedish Medical Products Agency (Läkemedelsverket) (MPA) for use in the trial.

13.2 Expected adverse reactions/serious adverse reactions (AR/SARs)

All expected ARs are listed in the latest MHRA and MPA approved version of the RSI as specified in section 13.1.7. These must be used when making a determination as to the expectedness of the AR. If the AR meets the criteria for seriousness, this must be reported as outlined below.

13.3 Evaluation of adverse events

All AEs, including events related to the nIMPs or the R-L device, must be recorded from the point of informed consent regardless of whether a participant has yet received an investigational product. Individual AEs should be evaluated by the investigator. This includes the evaluation of its seriousness, and any relationship between the AE (causality) and:

- the ATIMP (the STEM-PD product); and/or,
- the surgical procedure; and/or
- concomitant therapies.

AEs related to the nIMPs will not be reported as part of this trial unless the event results from a possible interaction with the ATIMP. AEs related to the nIMPs will be reported as per relevant normal processes.

Adverse events related to the R-L device will be reported to the manufacturer, only in the event of a malfunction or deterioration of the devices' properties or performance, and/or the event of errors and deficiencies in labelling or instructions for use that may lead to or have led to:

- the death of a patient, a user or another person, or
- a serious deterioration in the health of a patient, a user or another person.

The STEM-PD trial coordinator is responsible for onward reporting of such events to Medicinsk Teknik Skåne (as the manufacturer of the R-L device). Medicinsk Teknik Skåne is responsible for onward reporting to the Health and Social Care Inspectorate (IVO) in Sweden.

13.3.1 Assessment of seriousness

Seriousness is assessed against the criteria in section 13.1.5. This defines whether the event is an AE, SAE or SAR.

13.3.2 Assessment of causality

Definitely:	A causal relationship between the AE and IMP is clinically/biologically certain. **This is therefore an AR.**
Probable:	A causal relationship is clinically/biologically highly plausible and there is a plausible time sequence between onset of the AE and administration of the IMP and there is a reasonable response on withdrawal. **This is therefore an AR.**
Possible:	A causal relationship is clinically/biologically plausible and there is a plausible time sequence between onset of the AE and administration of the IMP. **This is therefore an AR.**
Unlikely:	A causal relation between the AE and the IMP is improbable and another documented cause of the AE is most plausible. **This is therefore an AE.**
Unrelated:	A causal relationship between the AE and the IMP can be definitely excluded and another documented cause of the AE is most plausible. **This is therefore an AE.**

Unlikely and unrelated causalities are considered not to be trial IMP related.
Definitely, probable and possible causalities are considered to be trial IMP related.

A pre-existing condition must not be recorded as an AE or reported as an SAE unless the condition worsens during the trial and meets the criteria for reporting or recording in the appropriate section of the case report form (CRF).

13.3.3 Clinical assessment of severity

Severity will be assessed using the following grading:

Grade 1: Mild; asymptomatic or mild symptoms; clinical or diagnostic observations only; intervention not indicated.

Grade 2: Moderate; minimal, local or non-invasive intervention indicated; limiting age-appropriate instrumental ADL.

Grade 3: Severe or medically significant but not immediately life-threatening; hospitalisation or prolongation of hospitalisation indicated; disabling; limiting self-care ADL.

Grade 4: Life-threatening consequences; urgent intervention indicated.

Grade 5: Death related to AE.

13.3.4 Recording of adverse events

AEs and ARs should be recorded in the medical notes and the appropriate section of the CRF and/or AE/AR log. SAEs and SARs should be reported to the STEM-PD trial coordinator as detailed in section 13.6, who will oversee further reporting as required.

13.4 Adverse events of special interest (AESIs)

The following events have been identified as AESIs for the STEM-PD trial:

- Stroke
- Major intracranial haemorrhage
- Central nervous system (CNS) infection
- Major worsening of the participant's PD or rapid and substantial worsening of their existing dyskinesias that does not respond to a reduction in dopaminergic therapy
- Clinically significant event which may be related to immunosuppressive treatment, such as atypical infections, malignancies (squamous skin cancer, Kaposi's sarcoma, and lymphomas) and metabolic events (osteoporosis, diabetes)
- Any event occurring up to 24 hours post-transplantation (not including well-known surgery relating events, e.g. nausea, malaise).

AESIs should be recorded in the medical notes and the appropriate section of the CRF and the AESI report. AESIs should be reported to the STEM-PD trial coordinator as detailed in section 13.5, who will oversee further reporting as required.

13.5 Reporting of AESIs

Sites are expected to report AESIs to the STEM-PD trial coordinator as soon as possible and within 5 working days of being made aware of an AESI. Details of where to report AESIs can be found on the STEM-PD Trial AESI form and the front cover of the protocol.

Where events also meet the definition of a SAE, SAR or SUSAR, the relevant reporting timelines apply.

13.6 Reporting of SAEs

The Principal Investigator is responsible for ensuring that the assessment of all SAEs for expectedness and relatedness is completed and the onward notification of all SAEs to the Sponsor immediately but not more than 24 hours of first notification. The Principal

Investigator must include the trial coordinator in all communications regarding SAEs. The Sponsor must keep detailed records of all SAEs reported to them by the trial team.

The Principal Investigator is also responsible for prompt reporting of all SAE findings to the competent authorities (i.e. MHRA and MPA) if they could:

- adversely affect the health of participants
- impact on the conduct of the trial
- alter the risk to benefit ratio of the trial
- alter the competent authority's authorisation to continue the trial in accordance with Directive 2001/20/EC

The completed SAE form should be emailed. Details of where to report the SAEs can be found on the STEM-PD Trial SAE form and the front cover of the protocol. Any SAE reported without an assigned causality will be assumed to be related to the ATIMP and expedited reporting will be undertaken in accordance with section 13.7.

Copies of the SAE report will be shared with Novo Nordisk.

For any SARs, a copy of the completed SAE form will also be shared with the ATIMP manufacturer.

13.7 Reporting of SUSARs

All suspected ARs which occur in the concerned trial, and that are both unexpected and serious (i.e. SUSARs) are subject to expedited reporting. Refer to section 13.1.7 for the RSI to be used in this trial.

13.7.1 Who should report and whom to report to?

The Sponsor delegates the responsibility of notification of SUSARs to the Principal Investigator. The Principal Investigator or delegated trial team member must report all the relevant safety information previously described, to:

- the Sponsor
- the competent authorities (i.e. MHRA and MPA)
- the ethics committees

The Principal Investigator or delegated trial team member shall inform all investigators concerned of relevant information about SUSARs that could adversely affect the safety of participants.

A copy of the completed SAE form will also be shared with the ATIMP manufacturer and with Novo Nordisk.

Reporting to Eudravigilance will be completed by the MPA on behalf of the Sponsor for all participants (whether recruited in the UK or Sweden).

13.7.2 When to report?

13.7.2.1 *Fatal or life-threatening SUSARs*

All parties listed in section 13.7.1 must be notified as soon as possible but no later than **7 calendar days** after the trial team and the Sponsor have first knowledge of the minimum criteria for expedited reporting.

In each case, relevant follow-up information should be sought and a report completed as soon as possible. It should be communicated to all parties within an additional **8 calendar days**.

13.7.2.2 *Non-fatal and non-life-threatening SUSARs*

All other SUSARs and safety issues must be reported to all parties listed in section 13.7.1 as soon as possible but no later than **15 calendar days** after first knowledge of the minimum criteria for expedited reporting. Further relevant follow-up information should be given as soon as possible.

13.7.3 How to report?

13.7.3.1 *Minimum criteria for initial expedited reporting of SUSARs*

Information on the final description and evaluation of an AR report may not be available within the required timeframes for reporting. For regulatory purposes, initial expedited reports should be submitted within the time limits as soon as the minimum following criteria are met:

a) a suspected IMP
b) an identifiable participant (e.g. trial participant code number)
c) an AE assessed as serious and unexpected, and for which there is a reasonable suspected causal relationship
d) an identifiable reporting source

and, when available and applicable:

- a unique clinical trial identification (EudraCT number or in case of non-European Community trials the trial protocol code number)
- a unique case identification (i.e. Sponsor's case identification number)

13.7.3.2 *Follow-up reports of SUSARs*

In case of incomplete information at the time of initial reporting, all the appropriate information for an adequate analysis of causality should be actively sought from the reporter or other available sources. Further available relevant information should be reported as follow-up reports.

In certain cases, it may be appropriate to conduct follow-up of the long-term outcome of a particular reaction.

13.7.3.3 *Format of the SUSARs reports*

Electronic reporting is the expected method for expedited reporting of SUSARs to the competent authorities. The format and content as defined by the competent authority should be adhered to.

13.8 Pregnancy reporting

Pregnancies will be reported for female participants from the point of signing the ICF to 15 months post-transplantation. For participant's partners, pregnancies will be reported until 15 months post-transplantation. All pregnancies should be reported to the STEM-PD trial coordinator using the relevant STEM-PD Trial Pregnancy Reporting Form within 24 hours of notification.

Should a participant become pregnant post-surgery, the participant will be followed up as normal; however, immunosuppressive treatment will be stopped, and repeat PET scans and extended battery MRI scans will not take place. Safety MRI scans will be performed where possible.

Pregnancy is not considered an AE unless a negative or consequential outcome is recorded for the mother or child/foetus. If the outcome meets the serious criteria, this would be considered an SAE.

14 Toxicity – Emergency Procedures

In the event that a participant develops a major intracranial complication following the transplant (for example a tumour or a large bleed) they should attend hospital as an emergency. Participants will be provided with contact details for the research team and would be encouraged to contact the research team where possible in the case of an emergency. These events must be reported in accordance with section 13.

15 Evaluation of Results

15.1 Primary response criteria

15.1.1 AEs and SAEs in the first 12 months following transplantation

All safety events will be recorded from the point of signing the ICF until the end of trial participation and assessed as to whether they meet the definition of AE, AR, SAE, SAR, SUSAR or AESI (as outlined in section 13).

15.1.2 MRI in the first 12 months following transplantation

MRIs will be performed as outlined in the schedule of assessments (section 12.12). Specifically, MRIs will be assessed for any indication of space occupying masses, as well as for any other safety concerns relating to the transplantation of the STEM-PD product.

15.2 Secondary response criteria

15.2.1 Clinical effects at 36 months following transplantation

Clinical effects will be reviewed and recorded throughout the trial to 36 months post-transplantation. In particular, the following will be assessed and recorded:

- Any emergence of new neurological features, specifically any signs of GIDs
- Global cognitive changes, as determined by the cognitive assessments over the duration of the trial
- Changes in non-motor and QOL assessments

15.2.2 Changes in motor features in the OFF state

Throughout the course of the trial, participants will undergo a number of motor assessments in both the ON and OFF states. Changes in the motor assessments will be recorded during the course of the trial.

15.2.3 Change in anti-PD medication

At every visit during the trial, a review of concomitant medication (including anti-PD medication) will be performed and any changes recorded. Anti-PD medication will be recorded as a LED, and the change in LED will be monitored throughout the trial.

15.2.4 Change in F-DOPA uptake and DAT binding

The screening F-DOPA PET scans will act as the 'baseline' measurement for changes in F-DOPA uptake. The baseline PE2i will act as the 'baseline' measurement for DAT binding. Changes from this baseline measurement up to and including the 36 months post-transplant time point will be recorded.

15.2.5 AEs and SAEs in the 12 to 36 months period following transplantation

The difference between this and the primary response criteria (section 15.1.1) is that this specifically relates to any safety events reported after the 12 months post-transplant time point.

15.3 Evaluation of ON measurements

Where assessments are performed in the ON state, the results will be analysed in two separate groups. The first group will be assessments performed ON following the participants' usual dose of PD medications, and the second group will be those ON assessments that are performed on the same day as the L-dopa challenge test. The reason for this being that the L-dopa challenge test does not represent a 'normal' ON for the participants as they will receive a different challenge dose of L-dopa.

16 Storage and Analysis of Samples

16.1 Safety blood samples

Blood samples will be collected and analysed as per standard practice in the routine NHS Biochemistry, Haematology and Microbiology laboratories of Addenbrooke's Hospital or the Department of Laboratory Medicine at RS-SUS.

For participants in the UK, TPMT will be sent to Sandwell and Birmingham Hospital for analysis via standard of care processes at Addenbrooke's Hospital.

16.2 Optional research blood samples

Where the participant has given consent, the optional research DNA, whole blood, plasma and serum samples may be collected and analysed for an immune response. Some of the analysis will be performed locally at each site, although samples may be analysed centrally where this is feasible. Centralised samples will be stored in the John van Geest secure clinical suite, or in the Clifford Allbutt Building (CAB). Only authorised people at site will have access to the samples. Samples will be labelled with participant trial-specific identifiers.

The optional research blood samples collected at RS-SUS will be registered at Region Skåne Biobank and handled according to the applicable biobank law and regulations. This will not apply for samples analysed and/or discarded within the exemption period mentioned in the applicable biobank law. The samples are coded to protect the participant's identity. All samples and the code list are stored securely and separately to prevent unauthorised persons from accessing them.

Any remaining samples will be stored for future use in ethically-approved research if the participant has consented to this. Refer to the relevant STEM-PD Trial Laboratory Manual for further details.

16.3 Optional CSF samples

Where the participant has given consent, the optional CSF samples will be collected. Standard analysis will be completed on the CSF samples and will be performed locally at each site (in the routine NHS Biochemistry, Haematology and Microbiology laboratories of Addenbrooke's Hospital or the Department of Laboratory Medicine at RS-SUS). Local routine parameters will be measured such as cell count, protein and glucose levels, and presence of oligoclonal bands of immunoglobulins.

Additional testing of the CSF will be performed to analyse for an immune response. Some of the analysis will be performed locally at each site, although samples may be analysed centrally where this is feasible. Centralised samples will be stored in the John van Geest secure clinical suite, or at the CAB. Samples will be labelled with participant trial-specific identifiers and will only be accessible to authorised persons.

Any remaining CSF samples will be stored locally at each recruiting site. Only authorised people at site will have access to the samples.

CSF samples collected at the RS-SUS that are not analysed and/or discarded within the exemption period mentioned in the applicable biobank law, will be registered at Region Skåne Biobank and handled according to the applicable biobank law and regulations. The samples are coded to protect the participant's identity. All samples and the code list are stored securely and separately to prevent unauthorised persons from accessing them.

Any remaining CSF will be stored for future, ethically-approved research if the participant has consented to this. Refer to the relevant STEM-PD Trial Laboratory Manual for further details.

17 Statistics

17.1 Statistical methods

Full details of all statistical issues and planned statistical analyses will be specified in a separate statistical analysis plan (SAP), which will be finalised prior to the locking of the trial database.

No formal hypothesis will be performed for the primary outcome measure relating to the number and nature of AEs and SAEs in the first 12 months following transplantation. The statistical analysis will be mainly descriptive and the number of AEs/SAEs will be summarised.

No formal hypothesis will be performed for the primary outcome measure relating to the MRI in the first 12 months following transplantation. The statistical analysis will be mainly descriptive.

17.2 Interim analyses

An analysis will be performed once all of the first four participants (i.e. those on dose 1) have reached the 6 month post-transplant visit. This will at minimum include a review of safety events to date, changes on PET and MRI imaging, and any change in MDS-UPDRS part III scores.

An additional analysis will be performed once all of the first four participants (i.e. those on dose 1) have reached the 12 month post-transplant visit. This will at minimum include a review of safety events to date, changes on PET and MRI imaging, and any change in MDS-UPDRS part III scores.

17.3 Number of participants to be enrolled

No formal sample size calculations are planned as this is not a confirmatory trial, and the primary outcomes are descriptive. In addition, the trial is not powered for confidence intervals of a given width. Instead, four participants will be dosed with dose 1, followed by four participants with dose 2 (or dose 1), as this was judged to be an appropriate number to give us reliable information about the above outcomes, while not exposing a larger cohort to unknown risks. This number was derived in part from similar first in human studies done with transplants of human foetal VM DA cells and novel DA gene therapies, such as OXB-102 and ProSavin (Barker et al., 2013; Palfi et al., 2014).

17.4 Criteria for the premature termination of the trial

The trial does not have any pre-determined stopping criteria. However, premature termination would be triggered by concerns raised by the investigators and/or the DSMB regarding safety and ethical implications of continuing the trial.

17.5 Criteria for temporary halt of transplantations

A temporary halt to further transplantations will be triggered if:

- One of the participants develops a SAE, judged by the investigator or the Sponsor to be possibly, probably or definitely related to the STEM-PD product (i.e. an SAR); or,
- Severe (grade 3-5 as assessed according to section 13.3.3) non-serious ARs in two participants, independent of whether they are within or not within the same system organ class

In the case of a temporary halt, all investigators, the DSMB, and trial sites will be notified promptly. Regulatory authorities and ethics committees will be informed of the temporary halt within 15 days via substantial amendment.

In this event, follow-up of participants who have previously undergone transplantation will continue as per protocol.

If the trial is temporarily halted, the trial teams in Cambridge and Lund, and a representative of the Sponsor would need to make a formal decision to restart the trial, providing justification to do so. The DSMB will also be consulted prior to making this decision and will be asked to provide a recommendation on how to proceed. Restarting the trial will require a substantial amendment.

Apart from the above, discontinuation of a planned transplantation for an individual participant will be decided by the PI in consultation with the clinical sub-group of the TMG.

17.6 Procedure to account for missing or spurious data

All participants for which we have data will be included in the analysis. For participants that withdraw for any reason, their data prior to withdrawal will be used where possible. Results from analyses that deviate from the SAP will be distinguished from results that followed the SAP, either by presenting them in different places (e.g. tables or subsections) or with footnotes if presented together.

17.7 Definition of the end of the trial

The end of the trial will be defined as the date of the last participant's last visit. The end of the trial will not be declared until all samples in the UK have been analysed as per this protocol.

18 Data Handling and Record Keeping

Pseudoanonymised data, which may include imaging data, will be shared with Novo Nordisk and may be used for scientific purposes and to develop STEM-PD as a future treatment.

18.1 Case report form (CRF)

All data will be transferred into an eCRF, which will be pseudo-anonymised. All trial data in the CRF must be extracted from, and be consistent with, the relevant source documents. The CRFs must be completed, dated and signed by the investigator or designee in a timely manner. It remains the responsibility of the investigator for the

timing, completeness, legibility and accuracy of the CRF pages. The CRF will be accessible to trial coordinators, data managers, the investigators, clinical trial monitors, auditors and inspectors as required.

All data collected on paper must be clear and legible. Any errors should be crossed out with a single stroke so that the original entry can still be seen. Corrections should be inserted, and the change dated and initialled by the investigator or designee. If it is not clear why the change has been made, an explanation should be written next to the change. Typing correction fluid must not be used.

18.2 Source data

To enable peer review, monitoring, audit and/or inspection the investigator must agree to keep records of all participating participants (sufficient information to link records e.g., CRFs, hospital records and samples), all original signed ICFs and copies of the CRF pages.

For this trial source documents include but are not limited to: signed ICFs; participant medical records; radiology, laboratory and pharmacy records; questionnaires; and blood results.

18.3 Data protection & participant confidentiality

All investigators and trial site staff involved in this trial must comply with the requirements of the GDPR 2018 and additional applicable national/local rules.

For Cambridge: Investigators and trial site staff must comply with the UK Data Protection Act 2018. Investigators and trial site staff involved in this trial must also comply with the requirements of CUH and UCAM policies with regards to the collection, storage, processing, transfer and disclosure of personal information and will uphold the Act’s core principles.

The trial staff will ensure that the participants’ anonymity is maintained. The participants will be identified by initials and a participant ID on the CRF and any electronic database. All documents will be stored securely and only accessible by trial staff and authorised personnel.

Pseudoanonymised data will be shared between Cambridge and Lund to allow for analysis of one data set.

Identifiable data will be shared between Cambridge and Lund sites specifically for the purpose of arranging the surgery, PET imaging and extended battery MRI at RS-SUS for the Cambridge participants. Patient identifiable data will be held on secure network drives hosted by the University of Cambridge Clinical School’s Secure Server, which is ISO 27001 certified, and conforms with NHS Information Governance Toolkit and NHS Data Security Protection Toolkit. Access to this data will be restricted to authorised members of the trial team via a two-factor authentication system. Data transfers will be performed according to the NHS Code of Practice on Confidentiality using the Cambridge University Secure Research Computing Platform
Servers and a two-factor authentication system.

MRI scans collected at the WBIC are stored in an identifiable format, visible to WBIC staff. Imaging Data is not encrypted or anonymised, but is held on a secure, restricted access, password protected server. Data access is restricted to members of the research team, who may download the imaging data in a pseudo-anonymised format. Imaging data is likely to be held indefinitely.

Patient details generated in the WBIC as part of the scanning process will be held indefinitely on the WBIC Radiology Information System (RIS), equivalent to an electronic hospital records system. RIS data will be held on secure, restricted access, password protected servers only accessible by WBIC members of staff.

Pseudoanonymised images will also be shared between the sites and Imperial College London to allow for analysis of the imaging.

Global Kinetics Pty Ltd. is the provider and manufacturer of the PKG® system. The PKG® is programmed by the site before providing to the participant with the participant ID, date of birth and sex using a tablet computer. The data from the PKG® is collected from the device unit after the participant has returned it to the trial site. The PKG® is CE marked, class 2E in Europe and in the US it has a 510K clearance for assessment of Parkinsonian features. The electronic data from the PKG® is transferred by the trial site staff from the device to a tablet computer and uploaded to a server owned by Global Kinetics Pty Ltd., based in Ireland, to generate a report. The data from the PKG® device is analysed by Global Kinetics Pty Ltd. and provided back to the site. Data is stored on the tablet computer until the report has been received from Global Kinetics Pty Ltd., after which the data will be removed from the tablet. Data uploaded to the server in Ireland are stored for 8 years after the end of the trial. Product and cybersecurity insurance for the use of the PKG's is provided by Global Kinetics.

Pseudoanonymised data, including copies of safety reports, will be shared, in a restricted and confidential manner with Novo Nordisk.

18.4 Archiving of trial documentation

At the end of the trial, the Sponsor will arrange for all centrally-held trial-related documentation to be archived securely for a minimum of 30 years. Arrangements for confidential destruction will then be made. It is the responsibility of PIs to ensure data and all essential documents relating to the trial held at site are retained securely for a minimum of 30 years after the end of the trial, in accordance with national legislation.

Essential documents are those which enable both the conduct of the trial and the quality of the data produced to be evaluated and show whether the site complied with the principles of GCP and all applicable regulatory requirements.

Cambridge will notify the sites when trial documentation held at sites may be archived. All archived documents must continue to be available for inspection by applicable authorities upon request.

The trial-specific data stored on the University of Cambridge Clinical School Secure Server will be archived in line with the University of Cambridge policy.

18.4.1 Archiving of documentation relating to ATIMP traceability

In accordance with the Advanced Therapy Regulations (1394/2007/EC), all parties (the Sponsor, the manufacturer of the STEM-PD product and the investigator/institution where the STEM-PD product is used) should keep their parts of the traceability records for a minimum of 30 years after the expiry date of the product. These requirements will be set out in contractual agreements between the Sponsor and the parties.

To comply with the regulatory requirements, each responsible party must ensure that the information relating to the traceability and accountability, from the production of

the ATIMP to the recipient being administered the ATIMP, are archived for a minimum of 30 years after the expiry date of the product.

The following essential documents and traceability data must be retained by the investigator and institution responsible for the application of the ATIMP to the participant.

- Shipping records of the ATIMP
- CoA of the ATIMP
- Treatment allocation and decoding information
- Participant identification code list
- ATIMP accountability at the site, including final disposition of both used and unused product

These records contain relevant information for traceability purposes and at least the following minimum data set from these records should be kept for 30 years after the expiry date of the product, or longer if required by the terms of the CTA or by the agreement with the Sponsor:

- Identification of the investigator/institution
- Identification of the Sponsor
- Identification of the manufacturing site
- Product name/code
- Pharmaceutical form, route of administration, quantity of dosage units and strength
- Batch and/or code number
- Trial reference code
- Participant identification code list (links name of participant to trial number/code)
- Product expiry/re-test date
- Date of administration of the product to participant
- Records of any product that was unused or destroyed at site or transferred for research purposes, and its final status

The patient medical records must contain the product name/code, the trial reference code, the trial participant code, the administration date(s) and dose(s). This maintains the traceability chain and ensures that a link can be made back to the identity of the product and the further traceability records held by the investigator/institution, product manufacturer and Sponsor.

19 Trial Committees

19.1 Trial Management Group (TMG)

The TMG will comprise investigators from both Cambridge and Lund, as well as other individuals closely involved with the running of the trial. The TMG will aim to meet monthly (and at least every 3 months) to ensure all practical details of the trial are progressing well. Safety events and trial non-compliances will also be reviewed by the TMG.

19.1.1 TMG clinical sub-group

The clinical subgroup of the TMG is composed of the Lead Investigator, the Co-Investigators, the PIs from each site, the Neurosurgeons from each site, and the imaging team from each site.

19.1.1.1 *Approval for transplantation*

The clinical sub-group of the TMG will assess each potential participant and approve to move ahead with transplantation once the local PI has confirmed that the participant

meets all other eligibility criteria. At minimum, the following information should be provided by the PI to the clinical sub-group for consideration:

- Confirmation that the participant meets all inclusion criteria and none of the exclusion criteria
- Confirmation that the participant has a good response to dopaminergic therapies
- Confirmation there are no other concerns of the recruiting centre to allow the participant to proceed with the trial

In order for a participant to be approved for transplantation, the PIs for both the Cambridge and the Lund sites and the neurosurgeon from the Lund site must be present. Although not mandatory, the neurosurgeon from the Cambridge should be present where possible for Cambridge participants. A representative from the local PET team should also be available at this meeting where possible. However, if a representative from the imaging team cannot attend, a written assessment of the participant's F-DOPA PET scan (according to exclusion criteria) must be provided to the clinical sub-group ahead of the meeting in order for a decision on inclusion to be made.

The vote to approve transplantation must be unanimous.

19.1.1.2 *Dose escalation*

Additionally, the clinical sub-group will make the final decision on the dose change following receipt of the DSMB recommendation. Once participants 1-4 have been followed up for 6 months post-transplantation, the TMG will review the safety and imaging (MRI and PET) data along with the DSMB recommendations to determine what dose participant 5-8 will receive. In order for the decision to be made, at minimum, the following members should be in attendance:

- At least two-thirds of members of the clinical sub-group from each site
- Representation from both Cambridge and Lund sites
- The lead investigator for STEM-PD project
- The PI from both Cambridge and Lund sites
- The Sponsor/Investigator for RS-SUS
- At least 1 representative from the imaging, surgical and clinical neurology team

19.2 Data and Safety Monitoring Board (DSMB)

The DSMB will meet in person or by video/teleconference, with a minimum frequency of twice per year. Additionally, a meeting will be called once participants 1-4 have been followed up for 6 months post-transplant. This will be to assess preliminary safety and imaging data for the DSMB to make a recommendation on which dose participants 5-8 will receive – see section 19.2.1.

More frequent meetings may be required at the discretion of the committee chair, or if required for unexpected safety events.

The chair of the committee will be independent, as will 75% of its voting members. To allow a meeting to take place, a minimum of two-thirds of DSMB members (including the DSMB Chair) need to be present. The Lead Investigator for the STEM-PD Project, Protocol Author/PI for CUH and PI for RS-SUS should also be available for the meetings.

19.2.1 DSMB recommendation for dosing

The DSMB, will make a recommendation for the dosing once participants 1-4 have been dosed and data is available for imaging and clinical measurements, as well as safety reports, 6 months after the last patient has been grafted. The DSMB can recommend

either to: i) remain at dose 1; ii) proceed to dose 2; or, iii) wait longer to collect more data. The final decision will be made by the clinical sub-group of the TMG, after receiving confirmation of the DSMB's recommendation.

20 Ethical & Regulatory Considerations

REC (in the UK), the EPM (in Sweden), Health Research Authority (HRA) (UK only), Administration of Radioactive Substances Advisory Committee (ARSAC) (UK only) and MHRA/MPA must approve this trial as required for each country.

20.1 Ethical committee review

Before the start of the trial or implementation of any substantial amendment we will obtain approval of the trial protocol, protocol amendments, ICFs and other relevant documents e.g. advertisements and General Practitioner information letters (if applicable) from the REC (in the UK) and the EPM (in Sweden). All correspondence with the REC and the EPM will be retained in the TMF and relevant ISF.

Annual reports will be submitted to the REC (in the UK) and the EPM (in Sweden) in accordance with national requirements. It is the STEM-PD trial coordinator's responsibility to produce the annual reports as required.

20.2 Regulatory compliance

The trial will not commence until a CTA is obtained from the MHRA and/or MPA as per national requirements. The protocol and trial conduct will comply with the Medicines for Human Use (Clinical Trials) Regulations 2004 and any relevant amendments.

Development Safety Update Reports (DSURs) will be submitted to the MHRA and MPA in accordance with national requirements. It is the STEM-PD trial coordinator's responsibility to produce the annual reports as required.

20.3 Protocol amendments

Protocol amendments must be reviewed by, and agreement received from, the Sponsor for all proposed amendments prior to submission to the HRA, REC/EPM and/or MHRA/MPA.

The only circumstance in which an amendment may be initiated prior to HRA, REC/EPM and/or MHRA/MPA approval is where the change is necessary to eliminate apparent, immediate risks to the participants (i.e., urgent safety measures). In this case, accrual of new participants will be halted until the HRA, REC/EPM and/or MHRA/MPA approval has been obtained. Additionally, the sites are responsible for notifying the STEM-PD trial coordinator of any urgent safety measure.

The STEM-PD trial coordinator will then be responsible for notifying all trial investigators and regulatory authorities to ensure urgent safety measures are implemented as applicable across the centres.

20.4 Peer review

The protocol has been peer reviewed by members of the Research Advisory Committee at CUH.

20.5 Declaration of Helsinki and GCP

The trial will be performed in accordance with the spirit and the letter of the declaration of Helsinki, the conditions and principles of GCP, the protocol and applicable local regulatory requirements and laws.

20.6 GCP training

All trial staff must hold evidence of appropriate GCP training or undergo GCP training prior to undertaking any responsibilities on this trial. This training should be updated every 2 years or in accordance with the local site policy. Sites are responsible for ensuring evidence of GCP training is filed in the ISF, and provided to the STEM-PD trial coordinator if requested for central filing in the TMF.

21 Sponsorship, Financial and Insurance

21.1 Sponsorship

The trial is sponsored by RS-SUS.

21.2 Funding

The trial is funded by a grant from Novo Nordisk to LU.

21.3 Insurance

CUH, as a member of the NHS Clinical Negligence Scheme for Trusts, will accept full financial liability for harm caused to participants in the clinical trial caused through the negligence of its employees and honorary contract holders. There are no specific arrangements for compensation should a participant be harmed through participation in the trial, but no-one has acted negligently.

UCAM will arrange insurance for negligent harm caused as a result of protocol design and for non-negligent harm arising through participation in the clinical trial for participants from the Cambridge site. Travel insurance will be arranged via UCAM to cover participants, companions and research staff travelling to Lund for the transplant, PET and MRI imaging.

LU and RS-SUS will arrange insurance to cover the use of the STEM-PD product.

The participants in Sweden are covered by patient insurance (the Patient Injury Act). This insurance also covers the transplants in Lund and any harm caused to participants in the clinical trial caused through negligence of its employees and honorary contract holders for the trial participants from the UK. This includes any problems that may arise once participants from the UK return to the UK, if relating to actions at RS-SUS and/or LU.

22 Monitoring, Audit & Inspection

The PI at each site must make all trial documentation and related records available should an MHRA or MPA inspection occur. Should a monitoring visit or audit be requested, the PI must make the trial documentation and source data available to the Sponsor’s representative. All participant data must be handled and treated confidentially.

The monitoring frequency will be determined by an initial risk assessment performed prior to the start of the trial. A detailed monitoring plan will be generated detailing the frequency and scope of the monitoring for the trial. Throughout the course of the trial, the risk assessment will be reviewed and the monitoring frequency adjusted as necessary.

23 Protocol Compliance and Breaches of GCP

Prospective, planned deviations or waivers to the protocol are not allowed under the relevant regulations on clinical trials and must not be used.

Protocol deviations, non-compliances, or breaches are departures from the approved protocol. They can happen at any time but are not planned. They must be adequately documented on the relevant forms (templates will be provided in the ISF) and sites must report any to the STEM-PD trial coordinator. The STEM-PD trial coordinator will be responsible for onward reporting to the Sponsor as required.

Deviations from the protocol which are found to occur constantly again and again will not be accepted and will require immediate action and could potentially be classified as a serious breach.

Any potential/suspected serious breaches of GCP must be reported immediately to the STEM-PD trial coordinator, who will report as required to the Sponsor without any delay.

24 Publications Policy

Ownership of the data arising from this trial resides with the trial team. On completion of the trial the data will be analysed and tabulated and a final trial report prepared.

The DSMB will be given the opportunity to read and comment on publications before submission. DSMB members will be named, and their affiliations listed in the main report, unless they explicitly request otherwise.

Novo Nordisk will be given the opportunity to read and comment on publications before submission. However, Novo Nordisk will not be involved in the planning, execution, analysis or reporting of the trial.

Participants will be given the opportunity to receive a lay summary of the published results.

25 Protocol Amendments

Version number	Version date	Summary of changes
1.0	15 Oct 2021	Not applicable – initial version<br>*Submitted to MPA only*
2.0	20 Dec 2021	• Update to sponsorship<br>• Clarifications on nIMPs<br>• Clarifications on timings of day -1 assessments<br>• Removal of SARS-CoV2 antibody test and ABO incompatibility antibody testing<br>• Addition of video recording for semantic (animal naming) fluency<br>• Updates to expected AEs<br>• Minor grammatical corrections<br>*Submitted to MPA and EPM only*
2.1	16 Feb 2022	• Update of eligibility criteria:<br>○ Amend to: Hoehn & Yahr stage 2-3<br>○ Amend to: Disease duration > 10 years<br>○ Addition of: Symptoms that are not adequately controlled by existing oral anti-PD medications, as judged by the PI or other delegated clinician<br>• Correction of radiation dose information<br>• Clarification that the Hoehn and Yahr stage scoring is performed as part of the MDS-UPDRS<br>*Submitted to EPM only*
2.2	24 Feb 2022	• Additional secondary objective added: To determine the safety and clinical efficacy between doses of the STEM-PD product including assessment of whether there is a dose response effect<br>• Amendment of co-trimoxazole to INN (Trimethoprim & Sulfamethoxazole)<br>• Additional MADRS assessments at 28 days and 3 months post-transplant<br>• Urine pregnancy testing to be completed by female participants of childbearing potential monthly (and remotely where this doesn't coincide with trial visits)<br>• Addition of justification for the timing of trial objective evaluation<br>• Addition of further justification for the use of relevant NIMPs<br>• Clarification for INN NIMPs that formulations to be used can be any available product on the market<br>• Clarification that where trial immunosuppressive treatments may affect the effectiveness of certain contraceptive methods, the local organ transplantation team will be consulted.

		• Clarification that tacrolimus formulation to be used must be a formulation to be used twice a day (i.e., not a depot product).<br>• Clarification that Sandimmun Neoral® will be used as an alternative to tacrolimus<br>• Clarification of which formulations will be used for the L-dopa challenge agent<br>• Addition of potential effect of coronavirus 2019<br>• Removal of all expected adverse events<br>• Clarification that PI will be responsible for SAE reporting<br>• Amendment to criteria for temporary halt of the trial<br>• Amendment to Appendix II to remove expected adverse events<br>*Submitted to MPA only*
2.3	28 Jun 2022	• Position of TMG member Nicholas Lao-Kaim amended<br>• Clarification that the secondary objective measure which evaluates any dose effect can only be measured when the different doses are used in the trial<br>• Amendment to allow serum or urine pregnancy tests to be performed by site<br>• Amendment to testing for CMV and EBV to include IgM and IgG titres<br>• Clarification that where OFF state measurements are required, L-dopa/COMT inhibitors will be stopped for at least 12 hours, or at least 24 hours for long-acting dopamine agonists/MAO-B inhibitors<br>• Clarification that extended battery MRI scans will be performed in Lund only<br>• Removal of reference to specific version of MedDRA for AE coding<br>• Clarification of the instructions for any necessary interruption to the surgery, not just medically necessary interruptions<br>• Clarification that in the case of cancelled/delayed surgery, F-DOPA PET imaging will need to be repeated the scan was performed more than 6 months before the new surgery date.<br>• Addition of safety MR imaging for participants at 6, 12, and, 24 months post-surgery<br>• Inclusion of ± 1 month window for 9 month post-surgery imaging to be completed<br>*Submitted to MPA only*
3.0	04 May 2023	• Addition of clinicaltrials.gov number for the trial in place of placeholder for ISRCTN number.<br>• Update to contact details for Robert Morris<br>• Amendment to DSMB members Elna-Marie Larssons' title to Professor Emerita

		• Removal of inclusion criteria requiring a full vaccination dose for COVID-19 at least 3 weeks prior to consent. COVID-19 vaccine requirements will instead be recorded in the immunosuppression manual.<br>• Expansion of screening visit window from -90 days to -120 days.<br>• Expansion of baseline visit window from -42 days to -60 days.<br>• Removal of the 12 hour post-transplantation visit<br>• Amendment of the 24 hour and 48 hour post-transplantation visits to day 1 and day 2 respectively for clarity.<br>• Clarification that only participants from Cambridge will be assessed for the presence MRSA at baseline.<br>• A preoperative CT angiogram added to the surgical planning procedures to provide better blood vessel visualisation.<br>• Addition of 6 month timepoint for optional CSF and research blood sample collection<br>• Clarification that in the event of an interruption to surgery between the two hemispheres, the TMG clinical sub-group will also determine which pre-surgery assessments will be repeated.<br>• Allowance for the sponsor investigator to be present for ATIMP administration in the event the PI is unable to attend.<br>• Correction of erroneous ratio of trimethoprim & sulfamethoxazole to 1:5 (section 10.2.1.2).<br>• Removal of dosing schedule for prednisolone to allow for appropriate adjustments to be made to the dose according to clinical judgement.<br>• Erroneous reference to the trial procedures manual in section 12.13.1.1 corrected to SOP4.<br>• Amendment to AESI definition regarding substantially worsening existing dyskinesias to specify that only dyskinesias that do not respond to a reduction in dopaminergic therapy should be considered AESIs<br>• Clarifications to the cross match testing that will be performed<br>• Amendments to the handling and analysis of optional research blood and CSF samples<br>• Clarification that plasma is also collected as part of the optional research blood sample.<br>• Clarification that samples analysed and/or discarded within the exemption period in applicable biobank law will not be registered at Region Skåne Biobank.<br>• Clarification of minor points relating to data transfer to Novo Nordisk:

		○ Addition of statement of the overall context for the data transfer to Novo Nordisk (section 18)<br>○ Amendment of requirement for data to be shared with Novo Nordisk only via password protected email allowing data to be shared via other methods providing that data is encrypted, confidential and shared with restricted access (section 18.3).<br>• Clarification that ethical and regulatory approvals to commence the trial will be according to national requirements (section 20)
3.1	17 May 2023	• Removal of CSF sample collection at day 28 and month 12.<br>• Correction of minor errors:<br>○ Spelling of sulfamethoxazole amended<br>○ Trimethoprim + sulfamethoxazole amended to Trimethoprim-sulfamethoxazole<br>○ Spelling of ciclosporin amended<br>○ Correction to screening and baseline windows in the trial flow chart<br>• Clarification that trimethoprim-sulfamethoxazole is also known as co-trimoxazole<br>• Clarification that Sandimmun Neoral® equivalent in the UK is Neoral®.<br>• Clarification that at the Cambridge site staff training logs will not be maintained for Pharmacy staff only involved in the receipt, handling, and dispensing of the nIMPs at the and accountability records will not be kept for the nIMPs.<br>• Clarification that identifiable data will be shared with Invicro London to facilitate booking imaging.<br>• Confirmation that trial-specific data stored on the Cambridge Clinical School Secure Server will be archived in line with the University of Cambridge policy.<br>• Minor updates to the system for storing and sharing patient identifiable data.<br>• Minor clarifications to optional research blood sample collection.
3.2	28 Oct 2023	• Clarification that the end of the trial will not be declared until all samples in the UK have been analysed.<br>• Confirmation that Product and cybersecurity insurance for the use of the PKG's is provided by Global Kinetics.
4.0	19 Dec 2023	• Definition added to describe women of childbearing potential.

		• Addition of a trial visit within 2 weeks of stopping immunosuppression to more appropriately record nIMP compliance.<br>• Amendment to TPMT exclusion criterion to remove reference to specific value for TPMT deficiency. Participants will be excluded if the lab determines they have severely reduced TPMT activity (less than half of the lower normal TPMT activity level).<br>• Amendment to trial flow chart to reflect new and previous changes to the trial visits.<br>• Correction to the administration volume of dose 2 of the STEM-PD product which has been made to section 10.1.1.11.<br>• Correction to the methods for data transfer from CUH to Invicro London/Imperial College.<br>• Location for the storage of any centralised CSF or research blood samples added to section 16.2 and 16.3.<br>• UK participants will travel to Lund 7 days before planned surgery date as opposed to 6 days.<br>• Correction to Jenny Nelander name and contact details
4.1	14May2024	• Updates to abbreviation table<br>• SOP number for the Pharmacy Manual added<br>• SOP number for the Surgical Manual added<br>• PI to consider use of gastro-protection for the duration of immunosuppression<br>• All PET imaging to be completed at RS-SUS:<br>o Removal of Invicro imaging facilities and requirements<br>o Removal of simultaneous MRI scan for Cambridge participants<br>o Clarification that all F-DOPA PET scans will include a low dose CT scan<br>• Amendment to the PET imaging schedule:<br>o 6 month PET imaging optional for Cambridge participants<br>o 12 month PE2i/CT scan optional for Cambridge participants<br>o 24 month F-DOPA and PE2i scans optional for Cambridge participants<br>• Amendments to MRI (extended battery) schedule:<br>o 6 month scan optional for Cambridge participants<br>o 12 month scan optional for Cambridge participants<br>o 24 month scan optional for Cambridge participants<br>• Option for screening and safety MRI's for CUH participants to be completed at the WBIC.

		• Clarification to travel period pre-surgery for Cambridge participants<br>• Clarification in the timing of assessments that participants may also be seen for an additional visit at 15+ months<br>• For consistency, amendment of UK participants to Cambridge participants throughout<br>• Clarification that the assessments in the L-dopa challenge test will be repeated up to 120 minutes post challenge dose.<br>• Addition of data storage conditions for WBIC generated data<br>• Clarification that Cambridge participants may receive a lower radiation exposure due to the number of optional PET/CT scans
V4.2	25Jul2024	• MRSA screening for UK participants moved from screening visit to baseline visit<br>• PKG assessments made applicable to Lund participants only

26 References

Adler, A. F., Cardoso, T., Nolbrant, S., Mattsson, B., Hoban, D. B., Jarl, U., Wahlestedt, J. N., Grealish, S., Bjorklund, A., & Parmar, M. (2019). hESC-Derived Dopaminergic Transplants Integrate into Basal Ganglia Circuitry in a Preclinical Model of Parkinson's Disease. *Cell Rep*, *28*(13), 3462-3473 e3465. https://doi.org/10.1016/j.celrep.2019.08.058

Aldrin-Kirk, P., Heuer, A., Wang, G., Mattsson, B., Lundblad, M., Parmar, M., & Bjorklund, T. (2016). DREADD Modulation of Transplanted DA Neurons Reveals a Novel Parkinsonian Dyskinesia Mechanism Mediated by the Serotonin 5-HT6 Receptor. *Neuron*, *90*(5), 955-968. https://doi.org/10.1016/j.neuron.2016.04.017

Barker, R. A., Barrett, J., Mason, S. L., & Bjorklund, A. (2013). Fetal dopaminergic transplantation trials and the future of neural grafting in Parkinson's disease. *Lancet Neurol*, *12*(1), 84-91. https://doi.org/10.1016/S1474-4422(12)70295-8

Barker, R. A., & consortium, T. (2019). Designing stem-cell-based dopamine cell replacement trials for Parkinson's disease. *Nat Med*, *25*(7), 1045-1053. https://doi.org/10.1038/s41591-019-0507-2

Barker, R. A., & Foltynie, T. (2004). The future challenges in Parkinson's disease. *J Neurol*, *251*(3), 361-365. https://doi.org/10.1007/s00415-004-0320-8

Barker, R. A., Parmar, M., Studer, L., & Takahashi, J. (2017). Human Trials of Stem Cell-Derived Dopamine Neurons for Parkinson's Disease: Dawn of a New Era. *Cell Stem Cell*, *21*(5), 569-573. https://doi.org/10.1016/j.stem.2017.09.014

Braak, H., Bohl, J. R., Muller, C. M., Rub, U., de Vos, R. A., & Del Tredici, K. (2006). Stanley Fahn Lecture 2005: The staging procedure for the inclusion body pathology associated with sporadic Parkinson's disease reconsidered. *Mov Disord*, *21*(12), 2042-2051. https://doi.org/10.1002/mds.21065

Braak, H., Ghebremedhin, E., Rub, U., Bratzke, H., & Del Tredici, K. (2004). Stages in the development of Parkinson's disease-related pathology. *Cell Tissue Res*, *318*(1), 121-134. https://doi.org/10.1007/s00441-004-0956-9

Brundin, P., Pogarell, O., Hagell, P., Piccini, P., Widner, H., Schrag, A., Kupsch, A., Crabb, L., Odin, P., Gustavii, B., Bjorklund, A., Brooks, D. J., Marsden, C. D., Oertel, W. H., Quinn, N. P., Rehncrona, S., & Lindvall, O. (2000). Bilateral caudate and putamen grafts of embryonic mesencephalic tissue treated with lazaroids in Parkinson's disease. *Brain*, *123 (Pt 7)*, 1380-1390. https://doi.org/10.1093/brain/123.7.1380

Cardoso, T., Adler, A. F., Mattsson, B., Hoban, D. B., Nolbrant, S., Wahlestedt, J. N., Kirkeby, A., Grealish, S., Bjorklund, A., & Parmar, M. (2018). Target-specific forebrain projections and appropriate synaptic inputs of hESC-derived dopamine neurons grafted to the midbrain of parkinsonian rats. *J Comp Neurol*, *526*(13), 2133-2146. https://doi.org/10.1002/cne.24500

Communications, B. A. (2021). *Bayer to advance two first-of-its-kind cell and gene therapies for Parkinson’s disease*. Bayer. Retrieved 20 Jul from

Defer, G. L., Geny, C., Ricolfi, F., Fenelon, G., Monfort, J. C., Remy, P., Villafane, G., Jeny, R., Samson, Y., Keravel, Y., Gaston, A., Degos, J. D., Peschanski, M., Cesaro, P., & Nguyen, J. P. (1996). Long-term outcome of unilaterally transplanted parkinsonian patients. I. Clinical approach. *Brain*, *119 (Pt 1)*, 41-50. https://doi.org/10.1093/brain/119.1.41

Evans, J. R., & Barker, R. A. (2008). Neurotrophic factors as a therapeutic target for Parkinson's disease. *Expert Opin Ther Targets*, *12*(4), 437-447. https://doi.org/10.1517/14728222.12.4.437

Freed, C. R., Breeze, R. E., Rosenberg, N. L., Schneck, S. A., Kriek, E., Qi, J. X., Lone, T., Zhang, Y. B., Snyder, J. A., Wells, T. H., & et al. (1992). Survival of implanted fetal dopamine cells and neurologic improvement 12 to 46 months after transplantation for Parkinson's disease. *N Engl J Med*, *327*(22), 1549-1555. https://doi.org/10.1056/NEJM199211263272202

Freed, C. R., Greene, P. E., Breeze, R. E., Tsai, W. Y., DuMouchel, W., Kao, R., Dillon, S., Winfield, H., Culver, S., Trojanowski, J. Q., Eidelberg, D., & Fahn, S. (2001). Transplantation of embryonic dopamine neurons for severe Parkinson's disease. *N Engl J Med*, *344*(10), 710-719. https://doi.org/10.1056/NEJM200103083441002

Grealish, S., Heuer, A., Cardoso, T., Kirkeby, A., Jonsson, M., Johansson, J., Bjorklund, A., Jakobsson, J., & Parmar, M. (2015). Monosynaptic Tracing using Modified Rabies Virus Reveals Early and Extensive Circuit Integration of Human Embryonic Stem Cell-Derived Neurons. *Stem Cell Reports*, *4*(6), 975-983. https://doi.org/10.1016/j.stemcr.2015.04.011

Hagell, P., Piccini, P., Bjorklund, A., Brundin, P., Rehncrona, S., Widner, H., Crabb, L., Pavese, N., Oertel, W. H., Quinn, N., Brooks, D. J., & Lindvall, O. (2002). Dyskinesias following neural transplantation in Parkinson's disease. *Nat Neurosci*, *5*(7), 627-628. https://doi.org/10.1038/nn863

Hagell, P., Schrag, A., Piccini, P., Jahanshahi, M., Brown, R., Rehncrona, S., Widner, H., Brundin, P., Rothwell, J. C., Odin, P., Wenning, G. K., Morrish, P., Gustavii, B., Bjorklund, A., Brooks, D. J., Marsden, C. D., Quinn, N. P., & Lindvall, O. (1999). Sequential bilateral transplantation in Parkinson's disease: effects of the second graft. *Brain*, *122 (Pt 6)*, 1121-1132. https://doi.org/10.1093/brain/122.6.1121

Hauser, R. A., Freeman, T. B., Snow, B. J., Nauert, M., Gauger, L., Kordower, J. H., & Olanow, C. W. (1999). Long-term evaluation of bilateral fetal nigral transplantation in Parkinson disease. *Arch Neurol*, *56*(2), 179-187. https://doi.org/10.1001/archneur.56.2.179

Heuer, A., Kirkeby, A., Pfisterer, U., Jonsson, M. E., & Parmar, M. (2016). hESC-derived neural progenitors prevent xenograft rejection through neonatal desensitisation. *Exp Neurol*, *282*, 78-85. https://doi.org/10.1016/j.expneurol.2016.05.027

Hoban, D. B., Shrigley, S., Mattsson, B., Breger, L. S., Jarl, U., Cardoso, T., Nelander Wahlestedt, J., Luk, K. C., Bjorklund, A., & Parmar, M. (2020). Impact of alpha-synuclein pathology on transplanted hESC-derived dopaminergic neurons in a humanized alpha-synuclein rat model of PD. *Proc Natl Acad Sci U S A*, *117*(26), 15209-15220. https://doi.org/10.1073/pnas.2001305117

Kefalopoulou, Z., Politis, M., Piccini, P., Mencacci, N., Bhatia, K., Jahanshahi, M., Widner, H., Rehncrona, S., Brundin, P., Bjorklund, A., Lindvall, O., Limousin, P., Quinn, N., & Foltynie, T. (2014). Long-term clinical outcome of fetal cell transplantation for Parkinson disease: two case reports. *JAMA Neurol*, *71*(1), 83-87. https://doi.org/10.1001/jamaneurol.2013.4749

Kirkeby, A., Grealish, S., Wolf, D. A., Nelander, J., Wood, J., Lundblad, M., Lindvall, O., & Parmar, M. (2012). Generation of regionally specified neural progenitors and functional neurons from human embryonic stem cells under defined conditions. *Cell Rep*, *1*(6), 703-714. https://doi.org/10.1016/j.celrep.2012.04.009

Kirkeby, A., Nolbrant, S., Tiklova, K., Heuer, A., Kee, N., Cardoso, T., Ottosson, D. R., Lelos, M. J., Rifes, P., Dunnett, S. B., Grealish, S., Perlmann, T., & Parmar, M. (2017). Predictive Markers Guide Differentiation to Improve Graft Outcome in Clinical Translation of hESC-Based Therapy for Parkinson's Disease. *Cell Stem Cell*, *20*(1), 135-148. https://doi.org/10.1016/j.stem.2016.09.004

Kriks, S., Shim, J. W., Piao, J., Ganat, Y. M., Wakeman, D. R., Xie, Z., Carrillo-Reid, L., Auyeung, G., Antonacci, C., Buch, A., Yang, L., Beal, M. F., Surmeier, D. J., Kordower, J. H., Tabar, V., & Studer, L. (2011). Dopamine neurons derived from human ES cells efficiently engraft in animal models of Parkinson's disease. *Nature*, *480*(7378), 547-551. https://doi.org/10.1038/nature10648

Lang, A. E., & Lozano, A. M. (1998a). Parkinson's disease. First of two parts. *N Engl J Med*, *339*(15), 1044-1053. https://doi.org/10.1056/NEJM199810083391506

Lang, A. E., & Lozano, A. M. (1998b). Parkinson's disease. Second of two parts. *N Engl J Med*, *339*(16), 1130-1143. https://doi.org/10.1056/NEJM199810153391607

Lehnen, D., Barral, S., Cardoso, T., Grealish, S., Heuer, A., Smiyakin, A., Kirkeby, A., Kollet, J., Cremer, H., Parmar, M., Bosio, A., & Knobel, S. (2017). IAP-Based Cell Sorting Results in Homogeneous Transplantable Dopaminergic Precursor Cells Derived from Human Pluripotent Stem Cells. *Stem Cell Reports*, *9*(4), 1207-1220. https://doi.org/10.1016/j.stemcr.2017.08.016

Lewis, S. J., Caldwell, M. A., & Barker, R. A. (2003). Modern therapeutic approaches in Parkinson's disease. *Expert Rev Mol Med*, *5*(10), 1-20. https://doi.org/10.1017/S1462399403006008

Li, J. Y., Englund, E., Holton, J. L., Soulet, D., Hagell, P., Lees, A. J., Lashley, T., Quinn, N. P., Rehncrona, S., Bjorklund, A., Widner, H., Revesz, T., Lindvall, O., & Brundin, P. (2008). Lewy bodies in grafted neurons in subjects with Parkinson's disease suggest host-to-graft disease propagation. *Nat Med*, *14*(5), 501-503. https://doi.org/10.1038/nm1746

Li, J. Y., Englund, E., Widner, H., Rehncrona, S., Bjorklund, A., Lindvall, O., & Brundin, P. (2010). Characterization of Lewy body pathology in 12- and 16-year-old intrastriatal mesencephalic grafts surviving in a patient with Parkinson's disease. *Mov Disord*, *25*(8), 1091-1096. https://doi.org/10.1002/mds.23012

Li, W., Englund, E., Widner, H., Mattsson, B., van Westen, D., Latt, J., Rehncrona, S., Brundin, P., Bjorklund, A., Lindvall, O., & Li, J. Y. (2016). Extensive graft-derived dopaminergic innervation is maintained 24 years after transplantation in the degenerating parkinsonian brain. *Proc Natl Acad Sci U S A*, *113*(23), 6544-6549. https://doi.org/10.1073/pnas.1605245113

Lindvall, O., Rehncrona, S., Brundin, P., Gustavii, B., Astedt, B., Widner, H., Lindholm, T., Bjorklund, A., Leenders, K. L., Rothwell, J. C., Frackowiak, R., Marsden, D., Johnels, B., Steg, G., Freedman, R., Hoffer, B. J., Seiger, A., Bygdeman, M., Stromberg, I., & Olson, L. (1989). Human fetal dopamine neurons grafted into the striatum in two patients with severe Parkinson's disease. A detailed account of methodology and a 6-month follow-up. *Arch Neurol*, *46*(6), 615-631. https://doi.org/10.1001/archneur.1989.00520420033021

Mendez, I., Dagher, A., Hong, M., Gaudet, P., Weerasinghe, S., McAlister, V., King, D., Desrosiers, J., Darvesh, S., Acorn, T., & Robertson, H. (2002). Simultaneous intrastriatal and intranigral fetal dopaminergic grafts in patients with Parkinson disease: a pilot study. Report of three cases. *J Neurosurg*, *96*(3), 589-596. https://doi.org/10.3171/jns.2002.96.3.0589

Morizane, A., Kikuchi, T., Hayashi, T., Mizuma, H., Takara, S., Doi, H., Mawatari, A., Glasser, M. F., Shiina, T., Ishigaki, H., Itoh, Y., Okita, K., Yamasaki, E., Doi, D., Onoe, H., Ogasawara, K., Yamanaka, S., & Takahashi, J. (2017). MHC matching improves engraftment of iPSC-derived neurons in non-human primates. *Nat Commun*, *8*(1), 385. https://doi.org/10.1038/s41467-017-00926-5

Nolbrant, S., Heuer, A., Parmar, M., & Kirkeby, A. (2017). Generation of high-purity human ventral midbrain dopaminergic progenitors for in vitro maturation and intracerebral transplantation. *Nat Protoc*, *12*(9), 1962-1979. https://doi.org/10.1038/nprot.2017.078

Normile, D. (2018). *First-of-its-kind clinical trial will use reprogrammed adult stem cells to treat Parkinson's*. sciencemag.org. Retrieved 20 Jul from

Olanow, C. W., Goetz, C. G., Kordower, J. H., Stoessl, A. J., Sossi, V., Brin, M. F., Shannon, K. M., Nauert, G. M., Perl, D. P., Godbold, J., & Freeman, T. B. (2003). A double-blind controlled trial of bilateral fetal nigral transplantation in Parkinson's disease. *Ann Neurol*, *54*(3), 403-414. https://doi.org/10.1002/ana.10720

Palfi, S., Gurruchaga, J. M., Ralph, G. S., Lepetit, H., Lavisse, S., Buttery, P. C., Watts, C., Miskin, J., Kelleher, M., Deeley, S., Iwamuro, H., Lefaucheur, J. P., Thiriez, C., Fenelon, G., Lucas, C., Brugieres, P., Gabriel, I., Abhay, K., Drouot, X., . . . Mitrophanous, K. A. (2014). Long-term safety and tolerability of ProSavin, a lentiviral vector-based gene therapy for Parkinson's disease: a dose escalation, open-label, phase 1/2 trial. *Lancet*, *383*(9923), 1138-1146. https://doi.org/10.1016/S0140-6736(13)61939-X

Rehncrona, S. (1988). *Kirurgiskt instrument för implantation* (Sweden Patent No. https://tc.prv.se/spd/p/patent?token=03AGdBq27FXDQQSLhS8_VGq_Zg_YFIs3GsjC8L8IG_efto58r8DbSCDwJJSD1tL-1qGOoU8Qk7wdeyogIafD6qJBXgrj1GFHBxt7zWBEC42S_51oQ_CuRk7wM46uEUGRdrnLoBIuMSEnBBpv3S9nQKRwJQHn___328NI2_dndZmkWUlk9G7R996z3YMhzcy2VipEEAN2EaPJXJ72upgY1aLBK4Xj_Z29Tx-QkfTPRqv4xOono6TGAG5PJcpIAka9XILVii7ue8v-3cLn-YsuQ-DE8jOF6BvJiDTjB7o7un-zoIRjhkWE6SoQxD11T_58o-IxwstiO2k6XLX04NfEdcEp0nfd1EHdQyD9FVI8RZst0bxa7kgfhti7xLIVwk7l-zJx86tMb7TepisJy0J882ZnNuyNqUO-

cSeZ_9NHNLFBdBIBco0xeMPkrbv6vrZkzSdnEEaSxrdfJtaIXvYNUFaCU_s_Sv2Wz8jdWZ5H4gdjBvit3vjwZdbpxo&p1=hNfRGojipWl7eM42P9NdVA&p2=_O52ExDgQpU&content=SE8802250-4+%2F+468+270&lang=en&tab=1&hits=true&hitsstart=0&start=0

Schade, S., Mollenhauer, B., & Trenkwalder, C. (2020). Levodopa Equivalent Dose Conversion Factors: An Updated Proposal Including Opicapone and Safinamide. *Mov Disord Clin Pract*, *7*(3), 343-345. https://doi.org/10.1002/mdc3.12921

Schuepbach, W. M., Rau, J., Knudsen, K., Volkmann, J., Krack, P., Timmermann, L., Halbig, T. D., Hesekamp, H., Navarro, S. M., Meier, N., Falk, D., Mehdorn, M., Paschen, S., Maarouf, M., Barbe, M. T., Fink, G. R., Kupsch, A., Gruber, D., Schneider, G. H., . . . Group, E. S. (2013). Neurostimulation for Parkinson's disease with early motor complications. *N Engl J Med*, *368*(7), 610-622. https://doi.org/10.1056/NEJMoa1205158

Schupbach, W. M., Maltete, D., Houeto, J. L., du Montcel, S. T., Mallet, L., Welter, M. L., Gargiulo, M., Behar, C., Bonnet, A. M., Czernecki, V., Pidoux, B., Navarro, S., Dormont, D., Cornu, P., & Agid, Y. (2007). Neurosurgery at an earlier stage of Parkinson disease: a randomized, controlled trial. *Neurology*, *68*(4), 267-271. https://doi.org/10.1212/01.wnl.0000250253.03919.fb

Tiklova, K., Nolbrant, S., Fiorenzano, A., Bjorklund, A. K., Sharma, Y., Heuer, A., Gillberg, L., Hoban, D. B., Cardoso, T., Adler, A. F., Birtele, M., Lunden-Miguel, H., Volakakis, N., Kirkeby, A., Perlmann, T., & Parmar, M. (2020). Single cell transcriptomics identifies stem cell-derived graft composition in a model of Parkinson's disease. *Nat Commun*, *11*(1), 2434. https://doi.org/10.1038/s41467-020-16225-5

Voon, V., Krack, P., Lang, A. E., Lozano, A. M., Dujardin, K., Schupbach, M., D'Ambrosia, J., Thobois, S., Tamma, F., Herzog, J., Speelman, J. D., Samanta, J., Kubu, C., Rossignol, H., Poon, Y. Y., Saint-Cyr, J. A., Ardouin, C., & Moro, E. (2008). A multicentre study on suicide outcomes following subthalamic stimulation for Parkinson's disease. *Brain*, *131*(Pt 10), 2720-2728. https://doi.org/10.1093/brain/awn214

Wider, C., Pollo, C., Bloch, J., Burkhard, P. R., & Vingerhoets, F. J. (2008). Long-term outcome of 50 consecutive Parkinson's disease patients treated with subthalamic deep brain stimulation. *Parkinsonism Relat Disord*, *14*(2), 114-119. https://doi.org/10.1016/j.parkreldis.2007.06.012

Wijeyekoon, R., & Barker, R. A. (2009). Cell replacement therapy for Parkinson's disease. *Biochim Biophys Acta*, *1792*(7), 688-702. https://doi.org/10.1016/j.bbadis.2008.10.007

Witt, K., Daniels, C., Reiff, J., Krack, P., Volkmann, J., Pinsker, M. O., Krause, M., Tronnier, V., Kloss, M., Schnitzler, A., Wojtecki, L., Botzel, K., Danek, A., Hilker, R., Sturm, V., Kupsch, A., Karner, E., & Deuschl, G. (2008). Neuropsychological and psychiatric changes after deep brain stimulation for Parkinson's disease: a randomised, multicentre study. *Lancet Neurol*, *7*(7), 605-614. https://doi.org/10.1016/S1474-4422(08)70114-5

27 Appendices

27.1 Appendix I: Risk-benefit evaluation

27.1.1 Risks for participants in the trial

This section is structured according to the order events may occur.

27.1.1.1 *Risks of trial assessments*

Most tests and assessments are non-invasive and do not constitute any medical risks. Functional assessments of movement abilities will be made in the defined OFF medication state, which is 12-24+ hours after stopping their regular anti-PD medication (participants will stop medication at least 12 hours prior to the visit (for L-dopa/COMT inhibitors), and for at least 24-hours (for longer acting dopamine agonists/L-dopa medications and MAO-B inhibitors)). For the patient population included in this trial this does not pose any danger or risk but may be experienced as unpleasant by the participant due to reduced mobility and occasionally painful dystonic muscle cramps they experience when off their anti-PD medication. Given this, the number and frequency of OFF assessments has been reduced to a minimum.

Blood samples are part of regular medical practice and considered only a minor risk.

Lumbar punctures are optional, but is a routine procedure without any serious risks, but post puncture headaches may occur.

MRI examinations may result in claustrophobia but if this is a major problem it constitutes an exclusion criterion. There are several MRI scans planned and most will be performed without gadolinium contrast enhancement, but in the event of an abnormality being seen on the scan post-transplantation, a repeat scan with contrast may be undertaken. In the planning phase, a TOF angiogram will be undertaken at RS-SUS, if this does not result in a visualisation of vessels, contrast will be used. Gadolinium contrast is used for clinical purposes but will be limited due to reports of a possible accumulation in some tissues with its use.

One of the MRI scans may be performed under general anaesthesia in order to generate high resolution images for neurosurgical planning purposes. The investigation is likely to be short (approximately 45 minutes), and constitutes a low level of risk, but may lead to discomfort in the throat and mouth, and rarely infections and pneumonia.

PET examinations will be conducted a maximum of 9 times during the 3 year trial period and will consist of the fluorinated ligands [^{18}F]FDOPA (x5) and [^{18}F]FE-PE2i (maximum x4), each with target injected radioactivities of 185MBq. Low dose CT scans of approx. 0.3mSv will be acquired in conjunction with each PET scan performed at RS-SUS for attenuation correction of the emission data. Furthermore all participants will undergo a CT angiogram of approx. 1.5 mSv. Thus, the maximal effective dose patients may be exposed to by taking part in this study is 44.4mSv, equivalent to about 18 years average background exposure (~2.3-2.4mSv/yr for people living in the UK and Sweden) over the 3 year study period (i.e. 6 years average background exposure per year of study). Participants from the UK may receive reduced exposure due to the number of optional PET/CT scans

Participants are informed that this level of increased radiation on a population of 10,000 per year is estimated to increase cancer incidence from 2,000 to 2,005 over a 20-year period. This level of increased risk is within the scope of the recommended radiation

doses for medical examinations and is subject to the evaluation of the relevant control agencies (in Sweden the EPM; and in the UK the ARSAC committee).

27.1.1.2 *Risks related to the immunosuppressive treatment*

The immunosuppressive treatment aims at protecting the grafted cells from host immune rejection. Intracerebral implantation results initially in a more effective host immunization compared with other implantation sites.

Once the graft is matured and integrated within the brain parenchyma, the grafted cells will be shielded from immune responses by the brain environment and the blood vessel properties. Risk of graft rejection after cessation of immunosuppressive treatment at 12 months is expected to be low because the brain is a relatively immunologically privileged site and we know that the nerve cells that develop in the cell preparation express low levels of immunological epitopes. This coupled to our combined experience gained from more than 250 patient years after grafting of human foetal cells means that immunosuppression can be withdrawn after about 12 months of treatment without risking graft rejection.

There are several risk aspects of immunosuppressive treatment.

1. Early in the course after transplantation, if the immunosuppressive treatment is interrupted for a period of time, this may lead to rejection and loss of any transplant benefits, or as a consequence lack of transplant development into DA producing cells.
2. During the induction phase (day -1 and first week) ARs may occur related to the higher dosages in this phase that are specific for the various medications used. The proposed combination encompasses corticosteroids, azathioprine and tacrolimus. High dose IV corticosteroid may induce mania and psychosis in a low number of patients, which may have serious consequences should this reaction occur. PD patients cannot readily be treated with neuroleptics in the acute phase to ameliorate these problems and there are no intravenous/subcutaneous preparations of the atypical neuroleptics that can be given to PD patients. However, it is likely that in the case of emergency treatment being needed, benzodiazepine administration will be sufficient in the rare event of such reactions. Serious early ARs to the induction dosages of tacrolimus are also common and involve confusion and other neurological symptoms, which in relation to intracerebral surgery can be expected to occur at a higher rate than reported.
3. The long-term risks involved with effective immunosuppressive treatment are outlined below. Generally risks are related to the cumulative dose of immunosuppressive drugs. In this trial, the duration of immunosuppression is short (12 months).
    a. Increased risk of new infections: The risk for new infections can be mitigated by participant education on recognising symptoms of typical infections, careful hygiene rules for handling food, interacting with infected persons in the surrounding area, and instructions on how to alert the trial team for early interventions when they have suspected symptoms.
    In view of the present pandemic of COVID-19, chronic immunosuppression will make the recipients enter a higher risk category. However, there are examples of patients with ongoing immunosuppressive treatment infected by COVID-19 in which both the grafted organ and the pneumonia and viral pneumonitis have been successfully managed. Patients with PD are otherwise not at risk for a

severe course of the COVID-19 infection, unless in a very advanced stage of disease which is an exclusion criterion for this trial. Additionally, participants will be required to have received a COVID-19 vaccination in line with local guidelines.

b. Activation of latent infections: The risks for activation of latent infections can be mitigated by pre-screening for potential sites of latent infections including teeth and skin. Participants will also be screened for previous exposure to latent viruses (see section 12.13.2.9), which will guide investigations should any infections appear during treatment. Antibiotic prophylaxis will be given primarily against the pulmonary infection pneumocystis carinii, which is effectively avoided by regular dosages of trimethoprim and sulfamethoxazole every other day for the duration of the immunosuppressive treatment.
c. Malignancies: Chronic immunosuppression increases the risk for a limited number of malignancies, in particular those with a potential viral co-factor, skin and ear, nose and throat tumours, mucosa and haematopoietic system, including squamous skin cancer, Kaposi's sarcoma, and lymphomas whereas the risk for common cancers is not. The risk increases with the presence of certain viruses, the length of treatment and level of immunosuppression. Short term treatment (as will be used in this trial) limits this risk.
d. Metabolic effects: There are drug related side effects that are mainly metabolic and pathophysiological in nature. Corticosteroids increases the risk of osteoporosis, which can be counteracted using prophylactic treatments (see section 10.2.3). Longer term use of corticosteroids also increases the risk for diabetes type II, and this will be evaluated on a regular basis. Tacrolimus also can cause diabetes and so again this will be carefully monitored and treated as required using standard agents and approaches.
e. Risk after cessation of immunosuppressive treatment: Based on experience from animal experiments and previous clinical transplantations the immunosuppression will be tapered and withdrawn after 12 months. This may lead to immunological rejection and loss of the graft and with this any benefits. However, and in contrast to whole organ grafts, the STEM-PD product is a cellular graft that is secondarily vascularised by the host which makes a rejection process less likely. In the event though of a suspected rejection reaction, high dose IV corticosteroids will be given as specified in the STEM-PD Trial Immunosuppressive Manual.

27.1.1.3 *Risks related to the transplantation surgery*

The transplanted participants are exposed to the general risks connected with general anaesthesia and brain surgery.

The long general anaesthesia times, up to 10 hours, being used in this trial increases the risk of low brain perfusion, unless there is adequate blood pressure control during the anaesthesia – however, surgeries of this length are not uncommon in neurosurgery. The pulmonary ventilation may also be suboptimal with an increased risk for pneumonia after the surgery. The agents used for anaesthesia also renders the brain more sensitive to anti-parkinsonian medications with a high risk of confusional and hallucinatory states after long periods of surgery. All these risks can be mitigated by careful clinical handling of the parameters involved, reduction of long acting anti-PD medication ahead of surgery and a tapered re-introduction of the medication in the post-operative phase.

There is a risk of haemorrhage when undergoing intracerebral surgery. Each penetration of the implantation instrument carries a certain risk. By testing participants for factors contributing to higher individual risks (such as clotting abnormalities and the use of certain medications) as well as obtaining brain angiographic characterisations of the blood vessels in the planning phase before surgery, the risk can be minimised. Experience from previous implantation surgeries as well as stereotactic DBS surgeries indicate an expected risk for a serious haemorrhage of 0.5-1.0%.

Implantation of a graft also entails risk of transferring an infectious agent. The cell product has been tested and found to be sterile and the handling the cell material is limited and performed under sterile conditions or in a controlled operation suite with adequate aseptic/clean conditions. Thus, the risk of infection from the grafted material is minimal. The participants will also receive prophylactic antibiotic treatment, which is designed to match the current spectrum of antibiotic resistance pattern at each trial site at the time of surgery and may thus vary over the course of the trial.

Post-operative complications are by convention reported from the time of surgery and for an additional 28 day period and include all types of reactions, including pulmonary embolism, to superficial skin infections as well as serious complications. The local statistics for Lund and the DBS surgery over time and all previous intracerebral transplantations are 3.0% overall complications and for serious complications 0.1%.

27.1.1.4 *Risks of the STEM-PD product*

The cell product to be used in the trial is manufactured from a pluripotent embryonic stem cell line. The manufactured cells are no longer pluripotent but are dopaminergic progenitor cells. However, any residual pluripotent cells contained in the final STEM-PD product could in theory give rise to uncontrolled growth or development of a teratoma formation. The product (as well as earlier batches of the product) has been tested thoroughly for this both *in vitro* and in pre-clinical safety studies and no such abnormal growths or teratomas have ever been observed with the final cell product (for full details, refer to the IMPD, IB and ATIMP Risk Table). The risk of a teratoma in participants is considered to be extremely small. Furthermore, no tumours have been detected in any of the animal *in vivo* studies done to date using various research-grade versions of this stem cell product from several different hESC cell lines (n> 2,000 grafted animals). The frequent MRI scans after surgery aim to detect abnormal growth and formation of mass lesions in the brain at the graft site, both of which could indicate teratoma formation.

Other potential forms of tumorigenicity are somatic cell overgrowth or malignant transformation. The STEM-PD product has been studied for its potential to form tumours due to mutagenesis, and a battery of oncogenes and mutations thereof has been performed and none found, as specified in the IMPD, IB and ATIMP Risk Table.

Should a teratoma or other signs of a malignancy or mass lesion of unknown characterisation occur, further investigations will promptly be made to define the nature of the problem. This will be handled according to standardised clinical procedures for tumour development, including biopsy, multidisciplinary investigations and discussions of relevant clinical treatment options, and cannot be foreseen in detail. In addition, decisions on the continued use of immunosuppressive treatment will be addressed.

The ventral midbrain dopaminergic progenitor cells are the cells of interest in the STEM-PD product for the treatment of PD. However, the STEM-PD product does contain other cell types. One particular neural cell type which should ideally be avoided are large numbers of serotonergic neurons (5-HT+), as it is thought that the ratio between

serotonergic and dopaminergic neurons (unrelated to the cell dose) may potentially give rise to GIDs. No serotonergic neurons have been detected in the non-clinical studies using the STEM-PD GMP batch #3 product (see IB).

GIDs have been observed in a limited number of patients in previous foetal transplant studies. Aside from the serotonergic neurons, various theories on the cause of these dyskinesias exists, and number of surviving cell numbers have been suggested to play a role. There are however no good arguments based on clinical evidence and animal experiments trying to elucidate the critical factors for GID development that neither too low numbers, nor too high numbers of surviving grafted cells contribute to the development of GIDs. Other factors thought to contribute to GIDs is the degree of integration with synaptic connectivity and circuitry reformation, which both are less dependent on cell numbers rather than maturation and a normal course of development.

It is also worth noting that the doses being used in this trial are not estimated to carry any risk of overdosing, which has also never been seen in any clinical trial using foetal VM tissue. The two doses are below or in the range of the number of DA neurons that normally innervate the putamen in healthy humans, as fully outlined in section 6.4.2 of the IB.

In case GIDs do develop, medication will be adjusted according to clinical practice. If severe troublesome GIDs persist in spite of careful clinical management, and also in a prolonged OFF medication state, DBS treatment will be considered as has been done previously with some patients in receipt of human foetal VM transplants and as is standard for disabling uncontrolled LIDs in non-grafted PD patients.

Grafted cells could theoretically migrate to other sites and disrupt the function of neural circuits important for normal behaviour and movements. However, no sign of migration of the cells to other sites has ever been observed in the non-clinical Good Laboratory Practice (GLP) biodistribution study, as fully outlined in the IB.

Grafted cells could also be affected by the underlying disease of the host. Post-mortem analyses of several grafts of human foetal VM cells have indeed shown that grafted dopaminergic foetal neurons can acquire Lewy body pathology in form of α-synuclein inclusions, the pathological hallmark of Parkinson pathology (Li et al., 2008; Li et al., 2010; Li et al., 2016). However, the frequency of Lewy body pathology in grafted cells was reported to be very low (2% of DA neurons in a 12-year-old graft, 5% of DA neurons in a 16-year-old graft, and 12% in a 24-year-old graft) (Li et al., 2010; Li et al., 2016). In addition, the majority of the grafted cells seemed still functionally unimpaired several years after grafting, and patients with foetal VM graft have been reported to have long-term benefits (Kefalopoulou et al., 2014; Li et al., 2016). In the patient with the 24-year-old graft, extensive pathology was seen in all brain regions, which was probably the reason of the gradual loss of the earlier transplantation benefits (Li et al., 2016).

27.1.1.5 *Risks for the participants to remain on their regular anti-Parkinson treatment*

The participant population in this trial are similar to the patient population that could be expected to receive any of the advanced treatment options. The best equivalent treatment option is DBS according to the "Early Stim" study (Schuepbach et al., 2013), i.e. a large French-German study for which there are published long term follow-up.

In two studies (Schuepbach et al., 2013; Schupbach et al., 2007), about 270 patients with PD with a mean disease duration of 7.5 years and a mean age of 52 years, were randomised to best medical treatment or DBS of the subthalamic nucleus. 18% of the DBS operated patients had a complication relating to the surgery and the stimulation programming process and at 24 months 54% of the operated patients and 44% in the best medical treatment group had experienced a serious adverse event. Despite this, the primary end-point (PDQ-39 total score) showed improvement in the DBS group by 7.8 points and decreased by 0.2 points in the best medical treatment group, and the functional assessment showed similar differences.

Thus, there are risks in patients that remain on their current treatment and opt for an established more advanced treatment.

27.1.1.6 *Risks of the administration device*

The device used in the earlier trial with foetal tissue in Lund, Sweden has developed over time. The first generation of device was used for the first 2 patients (Lindvall et al., 1989) and a second generation of the implantation device was used in all subsequent implantations. The R-L device is described in detail in (Lindvall et al., 1989; Rehncrona, 1988). The R-L device has an outer diameter of 0.98 mm and the first-generation device had an outer diameter of 2.5 mm. A smaller instrument is thought to improve survival rate of the implanted cell, by reducing local brain trauma and improving the tissue environment where the cells are grafted. More than 200 individual implants have been in made using the R-L device in more than 20 patients, without device failure. There is evidence from autopsy data in patient that died of causes unrelated to the implantation procedure, several years after grafting, demonstrating that the device delivered large numbers of viable DA precursor cells (Li et al., 2008; Li et al., 2016).

27.1.1.7 *Risks of travelling post-surgery*

The Cambridge participants will travel back to the UK approximately 5 days post-surgery, assuming they are clinically fit to do so. Before travelling back, the participants will be assessed by the clinical team at RS-SUS to ensure there are no clinical concerns for the participants to travel. Additionally, at least one clinical member of the trial team from Cambridge will travel with the participants.

27.1.2 Benefits of participating in the trial

The STEM-PD product is specifically developed to replace the DA producing cells in the brain that disappear with PD. This is the first time this cell product will be given to patients meaning that it is unknown if the cell product has a beneficial effect in humans.

Previous studies with foetal transplants have shown some clinically beneficial effects on Parkinsonian symptoms, but also had limitations as explained in section 6.1. The preclinical studies with the STEM-PD product have shown that it is able to counteract the effects of lost DA neurons in experimental PD models, with a restoration of local DA innervation and function. This means that the product is a potential valuable clinical candidate for replacement of DA producing cells in patients with PD.

After transplantation, the grafted cells differentiate further and should integrate into the host brain. The time course of any clinically significant beneficial anti-parkinsonian symptomatic effects are unknown. The full development of symptomatic effects of transplanted foetal dopaminergic precursor cells took 2-3 years to develop after implantation, but the first effects were seen within 5-6 months of grafting. If the cells show a clinical effect, participants will be able to adjust and reduce their anti-PD

medication. Thus, any clinical benefits of the cell grafts will be to allow the participant to reduce or omit the need for advanced treatments of PD (as described in section 6.1).

27.1.3 Overall risk/benefit balance

Our overall risk – benefit evaluation is that this trial has a positive risk/benefit balance for participants.

27.2 Appendix II: Safety assessment flow chart

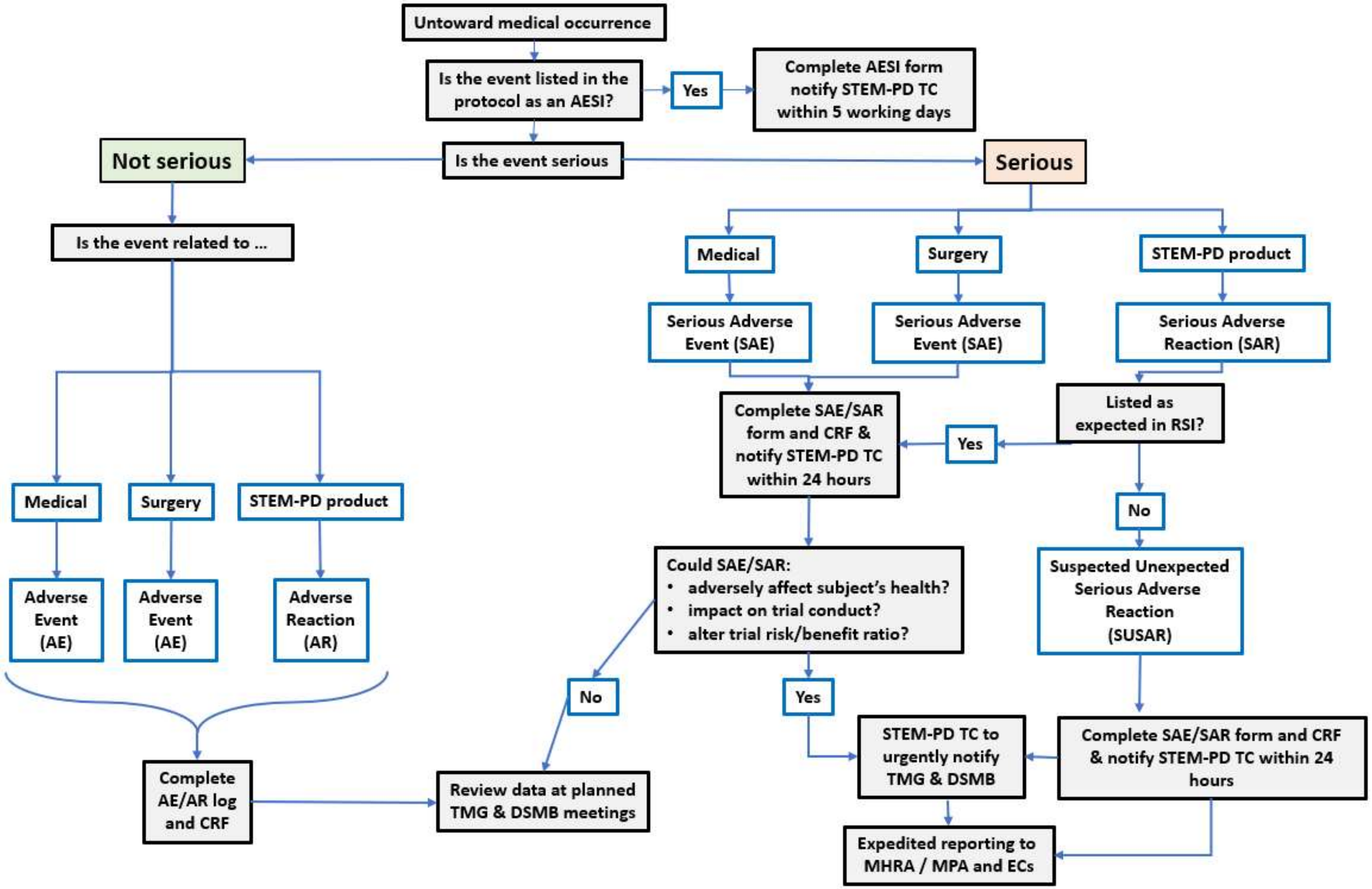

AESI = adverse event of special interest; **CRF** = case report form; **DSMB** = data safety monitoring board; **EC** = ethical committees (i.e. research ethics committee in the UK and the Swedish Ethical Review Authority in Sweden); **MHRA** = Medicines and Healthcare Products Regulatory Agency (UK); **MPA** = Swedish Medical Products Agency; **RSI =** reference safety information; **STEM-PD TC** = STEM-PD trial coordinator; **TMG** = trial management group

27.3 Appendix III: Planned staggered dosing schedule

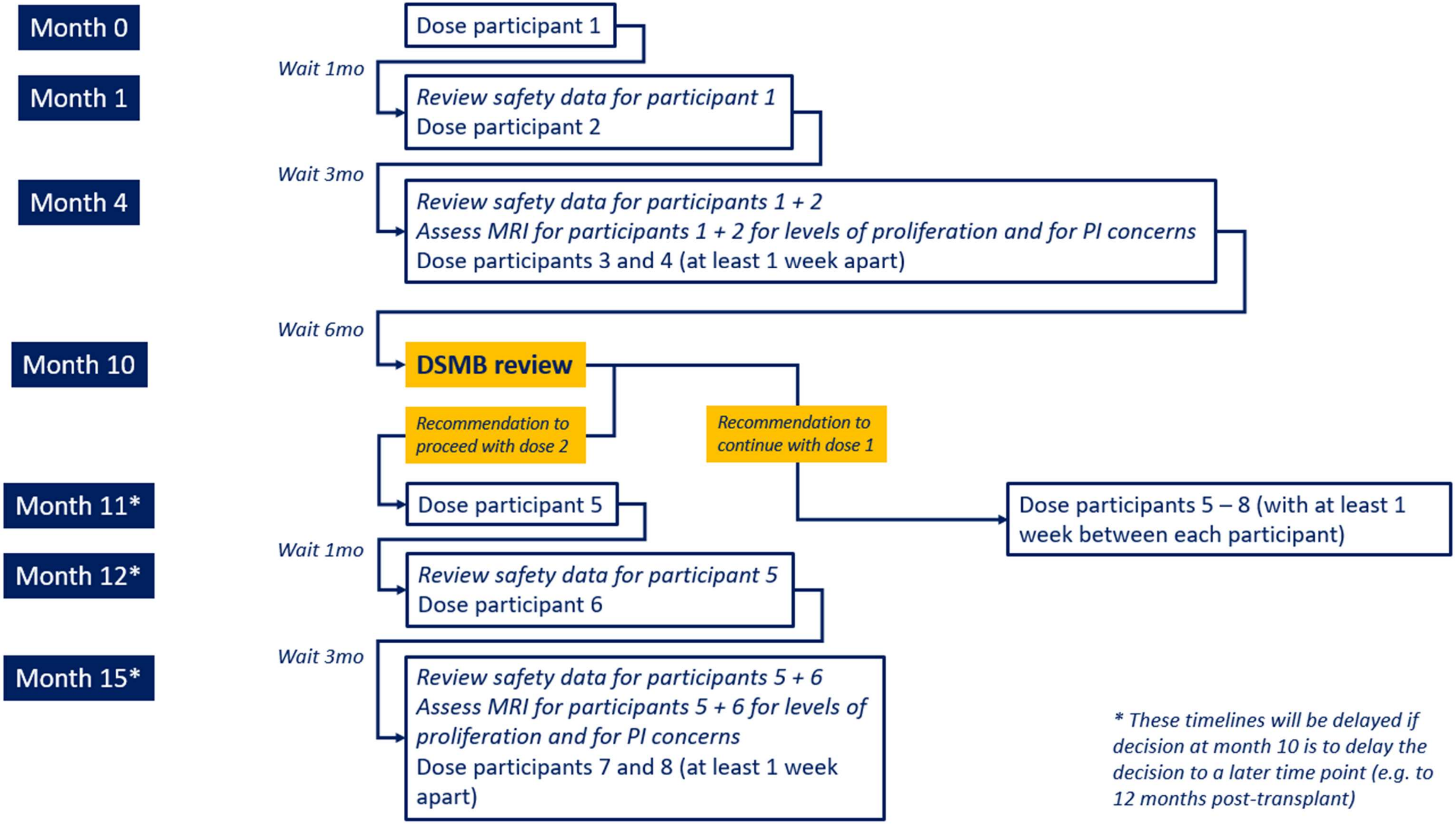

27.4 Appendix IV: Diagram of Rehncrona-Legradi device

**R-L injection device**<br>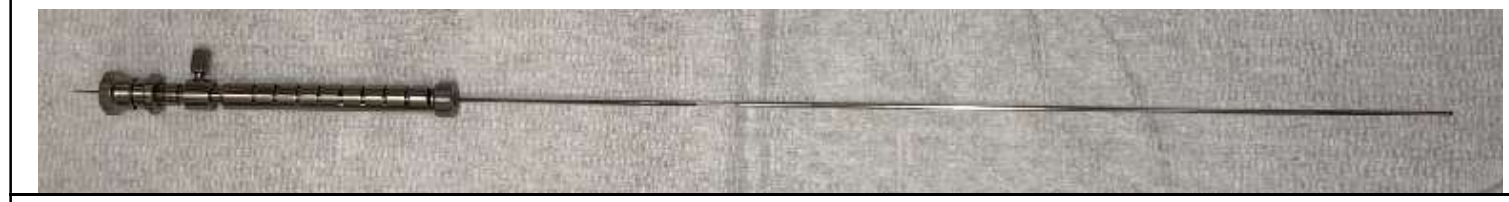	Device for delivery of cell suspension
**Guide cannula (bottom) and stylet (top)**<br>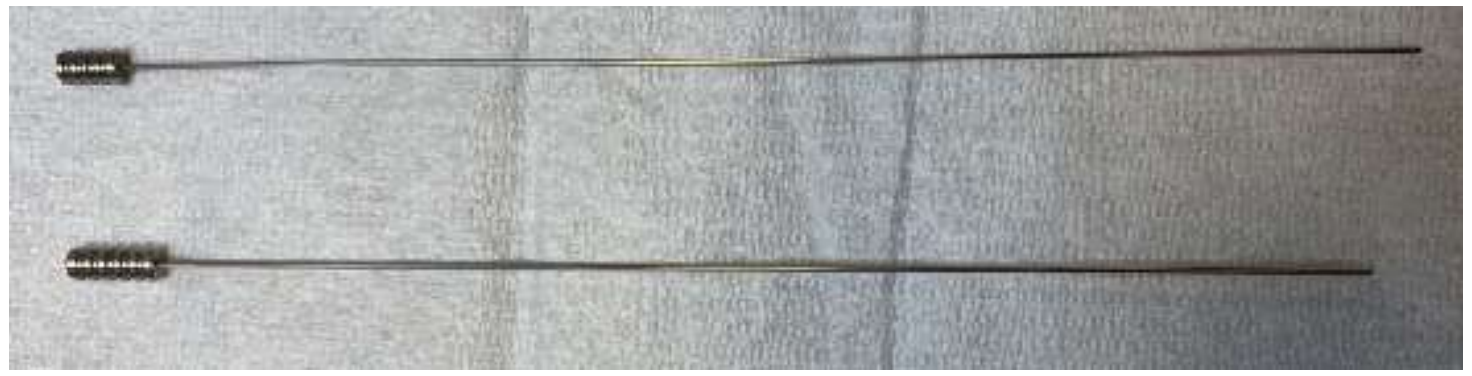	Guide cannula acts as pathway from outside brain to putamen<br>Stylet sits inside guide cannula upon insertion into brain
**Loading station (assembled)**<br>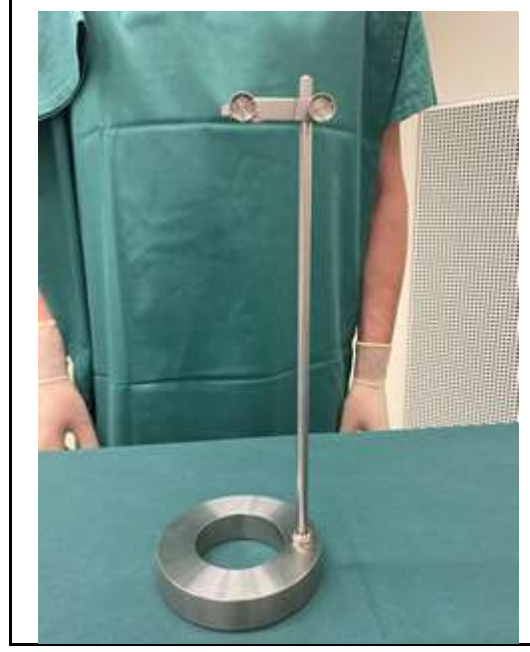	Loading station supports the sterile and precise loading of cell suspensions into R-L injection device

Continuous and ordinal variables will be presented using number of non-missing observations, number of missing observations, median, minimum (min) and maximum (max). The frequency (N) and percentages (%) (based on the non-missing sample size) of observed levels will be reported for all categorical variables.

9.1 Demographic and Baseline Variables

Gender, Age, LEDD, UPDRS-III OFF, UPDRS-III ON, disease duration, HY stage, time in OFF.

10 Analyses of primary outcomes

10.1 Adverse events (AEs) and serious adverse events (SAEs)

The AEs and SAEs within the first 12 months following transplantation will be presented as numbers and percentages. Primarily they will be categorized by medDRA system Organ Class (SOC) but in addition all related till NIMPs will be presented separately. Adverse events of special interest will be presented separately.

10.2 Absence of space occupying masses on cranial MRI

Absence of space occupying masses on cranial MRI in the first 12 months following transplantation will be presented as numbers and percentages.

11 Analyses of secondary outcomes

Descriptive statistics for measurements at baseline and at 36 months as well as absolute and relative differences will be presented as described in Section 6.

UPDRSIII in on and off state, AIMS sum score and PDG-39 will also be described using line charts for all available time points.

12 Analyses of exploratory outcomes

The analyses of the exploratory outcomes are not included in this statistical analysis plan and will be planned at a later stage.

13 Reporting Conventions

Minimum and maximum will be presented using the same number of decimal places as the original data. Median will be presented using one more decimal place than the original data. All percentages will be presented without decimals.
