# Supplementary material for: Human embryonic stem cell-derived dopaminergic cells for Parkinson’s disease: a phase 1/2 open-label trial
Source: Nat Med. 2026 Jul 9;32(9):3449–57. doi: 10.1038/s41591-026-04525-0 (PMC13577911; doi:10.1038/s41591-026-04525-0)
Supplement: Supplementary file 2 [file 41591_2026_4525_MOESM2_ESM.pdf]

nature portfolio

Corresponding author(s): Gesine Paul

Last updated by author(s): Jun 15, 2026

Reporting Summary

Nature Portfolio wishes to improve the reproducibility of the work that we publish. This form provides structure for consistency and transparency in reporting. For further information on Nature Portfolio policies, see our Editorial Policies and the Editorial Policy Checklist.

Statistics

For all statistical analyses, confirm that the following items are present in the figure legend, table legend, main text, or Methods section.

n/a	Confirmed	
☐	☒	The exact sample size (*n*) for each experimental group/condition, given as a discrete number and unit of measurement
☒	☐	A statement on whether measurements were taken from distinct samples or whether the same sample was measured repeatedly
☒	☐	The statistical test(s) used AND whether they are one- or two-sided<br>*Only common tests should be described solely by name; describe more complex techniques in the Methods section.*
☒	☐	A description of all covariates tested
☒	☐	A description of any assumptions or corrections, such as tests of normality and adjustment for multiple comparisons
☐	☒	A full description of the statistical parameters including central tendency (e.g. means) or other basic estimates (e.g. regression coefficient) AND variation (e.g. standard deviation) or associated estimates of uncertainty (e.g. confidence intervals)
☒	☐	For null hypothesis testing, the test statistic (e.g. F, t, r) with confidence intervals, effect sizes, degrees of freedom and P value noted<br>*Give P values as exact values whenever suitable.*
☒	☐	For Bayesian analysis, information on the choice of priors and Markov chain Monte Carlo settings
☒	☐	For hierarchical and complex designs, identification of the appropriate level for tests and full reporting of outcomes
☒	☐	Estimates of effect sizes (e.g. Cohen's *d*, Pearson's *r*), indicating how they were calculated

Our web collection on statistics for biologists contains articles on many of the points above.

Software and code

Policy information about availability of computer code

Data collection	eCRF data in RedCap
Data analysis	GraphPad Prism v 8.0 ; R version 4.2.3

For manuscripts utilizing custom algorithms or software that are central to the research but not yet described in published literature, software must be made available to editors and reviewers. We strongly encourage code deposition in a community repository (e.g. GitHub). See the Nature Portfolio guidelines for submitting code & software for further information.

Data

Policy information about availability of data

All manuscripts must include a data availability statement. This statement should provide the following information, where applicable:

- Accession codes, unique identifiers, or web links for publicly available datasets
- A description of any restrictions on data availability
- For clinical datasets or third party data, please ensure that the statement adheres to our policy

The study protocol and statistical analysis plan are included in Supplementary Note 1. The data sharing plan for this study is outlined at Clinicaltrials.gov and under Data availability Statement in the text. Source data if applicable are provided with this paper.

Research involving human participants, their data, or biological material

Policy information about studies with human participants or human data. See also policy information about sex, gender (identity/presentation), and sexual orientation and race, ethnicity and racism.

Reporting on sex and gender	1. The findings of this study apply to patients of all sexes; therefore, no restriction to a single sex or gender is applicable.<br>2. This study involved human participants. Sex was recorded for all participants based on self-reporting; however, sex and/or gender were not considered in the study design, and no stratification or analyses based on sex or gender were performed. Participants were included based on the study's clinical selection criteria, independent of sex or gender.<br>3. Sex-disaggregated data are available and can be provided. However, sex- and gender-disaggregated analyses are not presented, as sex and gender were not included as variables in the study design. Also, owing to the very small cohort size (n=8), sex-disaggregated analyses are not presented in the manuscript, as these would not be statistically informative and could compromise participant confidentiality.
Reporting on race, ethnicity, or other socially relevant groupings	Ethnicity data are not included.
Population characteristics	Demographic data is provided in the manuscript and detailed in table 1. Individuals between 50-75 were included.
Recruitment	All patients were recruited from an observational cohort TransEuro. There is likely a bias in that the patients are self-selected and this is an unblinded small study. The impact of this is unclear, but the small number of participants limits the generalisibility of the results. Larger cohorts are needed.
Ethics oversight	The study received ethical approval from the Swedish Ethical Review Authority (EPM Dnr 2021-06945-01) and the South Central – Oxford A Research Ethics Committee (Ref: 23/SC/0243).

Note that full information on the approval of the study protocol must also be provided in the manuscript.

Field-specific reporting

Please select the one below that is the best fit for your research. If you are not sure, read the appropriate sections before making your selection.

☒ Life sciences ☐ Behavioural & social sciences ☐ Ecological, evolutionary & environmental sciences

For a reference copy of the document with all sections, see nature.com/documents/nr-reporting-summary-flat.pdf

Life sciences study design

All studies must disclose on these points even when the disclosure is negative.

Sample size	This is a phase I study. Predetermined sample size was n=8 (n=4 per dose group), no sample size calculation was performed.
Data exclusions	No data were excluded
Replication	This is a human study and each particpant an n
Randomization	This study was not randomised. There was a sequential enrollment into the study.
Blinding	This was an unblinded (open label) study.

Reporting for specific materials, systems and methods

We require information from authors about some types of materials, experimental systems and methods used in many studies. Here, indicate whether each material, system or method listed is relevant to your study. If you are not sure if a list item applies to your research, read the appropriate section before selecting a response.

Materials & experimental systems

n/a	Involved in the study
☒	☐ Antibodies
☒	☐ Eukaryotic cell lines
☒	☐ Palaeontology and archaeology
☒	☐ Animals and other organisms
☐	☒ Clinical data
☒	☐ Dual use research of concern
☒	☐ Plants

Methods

n/a	Involved in the study
☒	☐ ChIP-seq
☒	☐ Flow cytometry
☐	☒ MRI-based neuroimaging

Clinical data

Policy information about clinical studies

All manuscripts should comply with the ICMJE guidelines for publication of clinical research and a completed CONSORT checklist must be included with all submissions.

Clinical trial registration

EudraCT 2021-001366-38; EU Trial Number 2024-511237-35-00; ClinicalTrials.gov: NCT05635409.

Study protocol

The study protocol is available as supplementary information and also published.

Data collection

Participants were recruited from two sites: Skåne University Hospital (Lund, Sweden) and Cambridge University Hospital (UK), with all surgical procedures performed at Skåne University Hospital. Recruitment occurred through a long-term observational cohort study (Transeuro observational study) .The first participant was enrolled on January 3rd, 2023 and the last on September 13th, 2024. Data was collected at predefined intervals (see study protocol) and is ongoing.

Outcomes

Primary endpoint: Safety, tolerability and feasibility of intraputamenal transplantation of the STEM-PD product in patients with moderate PD, measured by The number and nature of AEs and SAEs and absence of space occupying masses on cranial MRI in the first 12 months efter transplantation

Secondary endpoint: Course and efficacy on the clinical features and survival of dopamine cells following transplantation of the STEM-PD (e.g. changes in MDS-UPDRS motor score in OFF, LEDD, NMS, changes in 18F-Dopa PET) at 36 month

Plants

Seed stocks

NA

Novel plant genotypes

NA

Authentication

NA

Magnetic resonance imaging

Experimental design

Design type

Standard clinical MRI.Cranial MRI scans were acquired on a 3-T MAGNETOM Prisma scanner (Siemens Healthineers)

Design specifications

Safety MRIs were performed 2–3 days after transplantation and at months 1, 2, 3, 6, 9 and 12, and will also be performed at months 24 and 36

Behavioral performance measures

NA

Acquisition

Imaging type(s): Structural

Field strength: 3T

Sequence & imaging parameters: T1-weighted MPRAGE (axial), T2-weighted FLAIR (axial), T2-weighted TSE (coronal), diffusion-weighted imaging (axial), and susceptibility-weighted imaging (axial).

Area of acquisition: whole brain

Diffusion MRI: ☒ Used ☐ Not used

Parameters: axial, as part of safety assessment

Preprocessing

Preprocessing software: NA

Normalization: NA

Normalization template: NA

Noise and artifact removal: NA

Volume censoring: NA

Statistical modeling & inference

Model type and settings: NA

Effect(s) tested: *Define precise effect in terms of the task or stimulus conditions instead of psychological concepts and indicate whether ANOVA or factorial designs were used.*

Specify type of analysis: ☒ Whole brain ☐ ROI-based ☐ Both

Statistic type for inference (See Eklund et al. 2016): NA

Correction: NA

Models & analysis

n/a	Involved in the study
☒	☐ Functional and/or effective connectivity
☒	☐ Graph analysis
☒	☐ Multivariate modeling or predictive analysis
